# Persistent loss of intrahepatic IFN-γ in HBV is linked to selective impairment of liver-resident CXCR6+NK cells despite long-term NUC therapy

**DOI:** 10.1016/j.jhepr.2026.101865

**Published:** 2026-04-18

**Authors:** Boris J.B. Beudeker, Diren Arda Karaoglu, Shirin Nkongolo, Gertine W. van Oord, Zwier M.A. Groothuismink, Karishma A. Lila, Adam J. Gehring, Thierry van den Bosch, Robert J. de Knegt, Harmen J.G. van de Werken, Andre Boonstra

**Affiliations:** 1 Department of Gastroenterology and Hepatology, Erasmus Medical Center, Rotterdam, the Netherlands; 2 Internal Medicine IV (Gastroenterology, Hepatology, Infectious Diseases), University Hospital Heidelberg, Germany; 3 Molecular Virology, Center for Infectious Diseases, University Hospital Heidelberg, Germany; 4 Department of Pathology, Section Clinical Bioinformatics, Erasmus Medical Center, Rotterdam, The Netherlands; 5 Toronto Centre for Liver Disease, University Health Network, Toronto, Ontario, Canada; 6 Department of Pathology, Section Ophthalmic Pathology, Erasmus MC Cancer Institute, University Medical Center, Rotterdam, the Netherlands; 7 Department of Immunology, Erasmus University Medical Center, Rotterdam, the Netherlands

**Keywords:** Chronic hepatitis B, CXCR6 NK cells, NUC therapy, Liver immune cells, scRNAseq, TGF-β, Cytokine production, Viral suppression, Hepatitis B treatment

## Abstract

**Background & Aims:**

Chronic HBV infection remains incurable in most patients despite long-term nucleos(t)ide analog (NUC) therapy. Interferon-γ (IFN-γ) is a cornerstone of antiviral immunity, but its *in situ* source and status in the human liver remain unknown. We aimed to define the primary source of IFN-γ in healthy liver and determine how this axis is altered in stably suppressed NUC-HBV.

**Methods:**

Multiplex immunofluorescence for CD3, CD56, CXCR6, and IFN-γ was performed on liver biopsies from patients with NUC-HBV (n = 9) and healthy donors (n = 7), with whole-slide scanning and AI-based segmentation for unbiased *in situ* cell quantification. Single-cell RNA sequencing (scRNAseq) was conducted on paired blood and liver fine-needle aspirates (FNAs) from NUC-HBV (liver n = 9, blood n = 18) and integrated with control datasets (liver n = 5, blood n = 9). Differential gene expression was used for transcriptional readout, and cell–cell interaction analysis mapped altered signaling networks.

**Results:**

In healthy liver, CXCR6-positive natural killer (CXCR6+NK) cells were the dominant IFN-γ producers (24.2%; IQR 14.1–67.2%). In NUC-HBV, these cells were reduced (675 *vs.* 1,918; *p* = 0.012) and rarely expressed IFN-γ (0.2%; IQR 0.0–1.4%), despite normal alanine aminotransferase and absence of fibrosis. To validate transcriptionally, we performed scRNAseq on FNA and paired blood. A single, liver-restricted CXCR6+NK cluster was identified, and showed selective downregulation of *IFNG* and chemokines (*XCL1*, *CCL3*, *CCL4*), while cytotoxic genes (*GZMB*, *PRF1*) were preserved. Interaction analysis revealed reduced pro-inflammatory signaling and enrichment of transforming growth factor-beta–associated pathways. CXCR6+NK frequency correlated with serum HBsAg (*p* = 0.037) but was unchanged after 24 weeks of NUC in a longitudinal dataset.

**Conclusions:**

Long-term NUC therapy does not restore intrahepatic IFN-γ. Loss and transcriptional reprogramming of CXCR6+NK may contribute to a stable, altered immune state, representing a target for immune-based HBV cure strategies.

**Impact and implications:**

Effective antiviral therapy for chronic HBV suppresses viral replication but does not provide cure, indicating persistent defects in intrahepatic antiviral immunity. By combining protein-level analysis of liver tissue from healthy living donors and NUC-treated HBV liver biopsies with single-cell RNA-seq of fine-needle aspiration–derived immune cells from clinically stable, long-term NUC-treated patients, we directly examined IFN-γ regulation in the human liver—an immune compartment that is largely inaccessible in this patient group. We demonstrate that IFN-γ is produced *in situ* predominantly by CXCR6+NK cells in healthy liver and is selectively reduced in the liver of NUC-treated patients, despite normal alanine aminotransferase levels, absence of fibrosis, and lack of inflammatory or exhaustion signatures, while no corresponding defect is observed in blood. These findings identify a liver-restricted, non–exhaustion-driven impairment of NK-cell function and suggest that future therapeutic strategies should focus on restoring intrahepatic immunity—potentially via modulation of TGF-β–associated pathways or CXCR6+NK cell IFN-γ production.

## Highlights

• Multiplex immunofluorescence identifies CXCR6+NK cells as the dominant *in situ* source of IFN-γ in healthy human liver.
• In NUC-treated HBV, intrahepatic CXCR6+NK cells show near-complete loss of IFN-γ protein expression.
• scRNAseq of liver fine-needle aspirates shows transcriptional downregulation of IFNG but not cytotoxicity programs in CXCR6+NK cells.
• The IFN-γ defect is liver-restricted and not observed in blood, consistent with the tissue-restricted nature of CXCR6+NK cells.
• Cell–cell interaction analysis reveals reduced pro-inflammatory signaling and enrichment of TGF-β–associated pathways in NUC-HBV liver.

## Graphical abstract

**Figure:**
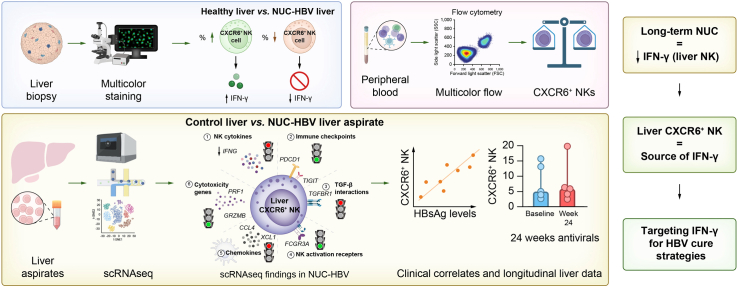

## Introduction

Chronic HBV infection affects over 250 million people globally and remains a leading cause of cirrhosis and hepatocellular carcinoma (HCC).^1^ While nucleos(t)ide analogs (NUCs) achieve durable viral suppression, they rarely result in functional cure because of the persistence of covalently closed circular DNA (cccDNA). As a result, long-term therapy is required, and new curative strategies are urgently needed.^2^^,^^3^

Interferon-gamma (IFN-γ) plays a central role in the control of HBV by enhancing antigen presentation, activating antiviral gene expression, and contributing to non-cytolytic suppression of viral replication and cccDNA activity, as shown in both human and experimental models.[4], [5], [6], [7], [8], [9], [10] In the liver, IFN-γ can be produced by multiple innate and adaptive immune subsets, including natural killer (NK) cells, which are highly enriched in hepatic tissue.^11^

A longstanding hypothesis in the HBV field suggests that intrahepatic IFN-γ production is diminished in chronic infection, particularly within NK cells.[12], [13], [14] However, direct evidence from human liver tissue is limited, and it remains unclear whether IFN-γ expression is restored under long-term NUC-mediated viral suppression.

To address this, we analyzed well-preserved liver tissue from organ donors and NUC-treated patients with HBV using multiplex immunofluorescence on core needle biopsies. This *in situ* approach allowed us to characterize immune cell phenotypes and cytokine expression in intact tissue sections, without the confounding effects of *ex vivo* stimulation. To validate and extend these observations, we performed single-cell RNA sequencing (scRNAseq) on unstimulated liver fine-needle aspirates (FNAs) and matched peripheral blood samples from a second HBV cohort, integrating the data with publicly available control liver datasets.

We observed consistently low IFN-γ expression in NUC-treated HBV livers, particularly within liver-resident CXCR6-positive natural killer (CXCR6+NK) cells. This intrahepatic finding was not mirrored in the circulation and appeared independent of NUC treatment, suggesting a persistent and liver-associated defect. These insights highlight intrahepatic IFN-γ loss as a stable feature of chronic HBV under NUC therapy and position liver-resident NK cells as a potential therapeutic target to restore antiviral immunity and advance curative strategies.

## Patients and methods

A detailed description of the methods is available in the supplementary materials. In brief, patients with chronic HBV from Erasmus MC, Rotterdam, The Netherlands were enrolled; they were adults who were HBeAg-negative with stable viral suppression (HBV DNA <80 IU/ml) and on NUC therapy for >3 years. Exclusion criteria were advanced fibrosis (elastography >7.0 kPa), hepatic decompensation, HCC history, coinfections, autoimmune or metabolic liver diseases, malignancies, or recent pregnancy. Formalin-fixed paraffin-embedded liver biopsies from patients with HBV and healthy controls were obtained through our pathology biobank; these samples were not stimulated to preserve their *in situ* immune state. Sections were stained for CXCR6, IFN-γ, CD3, and CD56 using the Ventana Discovery ULTRA system (Roche Ventana Medical Systems, Tucson, AZ, USA) , and whole-slide imaging was performed on a Zeiss Axioscan 7 (Carl Zeiss AG, Oberkochen, Germany). Quantification was conducted using Visiopharm® software (Visiopharm A/S, Hørsholm, Denmark) with AI-based segmentation.

Peripheral blood mononuclear cells (PBMCs) were isolated from heparinized blood using Ficoll separation. Intrahepatic leukocytes were obtained via FNAs and processed for scRNAseq within 1 h to maintain the unstimulated *in situ* transcriptional profile. scRNAseq was performed using the 10 × Genomics Single Cell 3′ and 5′ kits and sequenced on the NovaSeq6000 platform (Illumina, San Diego, CA, USA). In addition to our own data, we incorporated publicly available human liver and PBMC scRNAseq datasets (GSE136103; GSE155698; and GSE157789).[15], [16], [17] Quality control, normalization, batch integration, and clustering were performed using scanpy and scvi tools. PBMCs were additionally stained with antibodies targeting NK cell markers, including CXCR6 and CD56, and analyzed on a BD Symphony A3 cytometer (Becton, Dickinson and Company, Franklin Lakes, NJ, USA).

### Ethics

This study was conducted according to the guidelines of the Declaration of Helsinki and the principles of Good Clinical Practice. The ethical review board of the Erasmus MC approved the study, registered as MEC-2008-146 and MEC-2010-039.

## Results

### In healthy liver, CXCR6+NK cells are the dominant source of IFN-γ protein under steady-state conditions

**Fig. 1:**
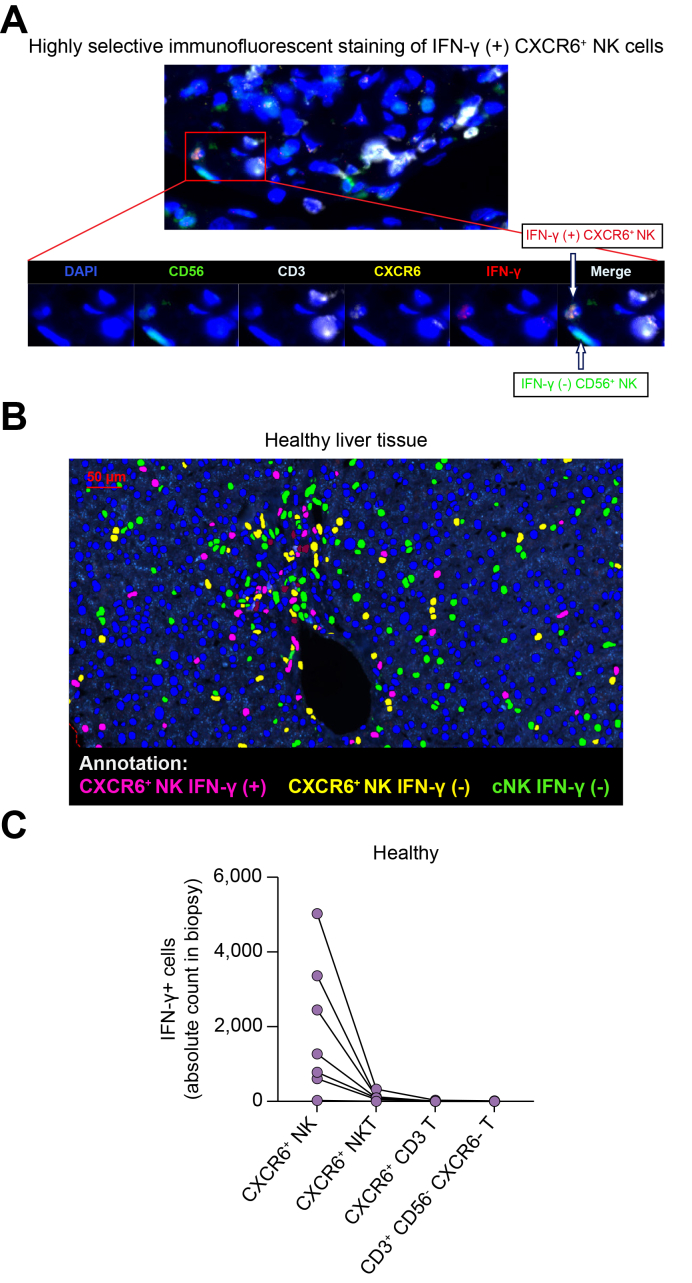
CXCR6+NK cells are the dominant source of in situ IFN‑γ in healthy human liver (A) Immunofluorescent staining to illustrate staining of IFN-γ+ CXCR6+NK cells in a healthy liver, using antibodies against CD56, CD3, CXCR6, IFN-γ, and nuclear staining with DAPI. Individual channels show DAPI (blue), CD56 (green), CD3 (white), CXCR6 (yellow), and IFN-γ (red). The merged image includes close-up views of a CD56 monostain, an IFN-γ+ CXCR6+NK cell, a CXCR6+ CD3+ cell, and a CD3 T cell. (B) Algorithmic phenotyping of immune cells in healthy liver tissue. Image analysis was performed using pathologist-supervised machine learning for unbiased identification and classification of immune cells. Mean fluorescence intensity (MFI) thresholds for DAPI, CD3, CD56, CXCR6, and IFN-γ were determined per slide and corrected for background. Every nucleated cell in the stained tissue was evaluated against these thresholds, and positive cells were phenotyped accordingly. For clarity, only CD56+ cells are color-coded in the algorithmic overlay. The control liver sample shows widespread parenchymal IFN-γ+ CXCR6+NK cells (pink), CXCR6+ IFN-γ- NK cells (yellow), CD56dim/bright NK cells (green), and CXCR6+ IFN-γ+ CD3+ NKT cells (bordeaux). (C) Comparison of absolute counts within each individual tissue section of IFN-γ+ cells per biopsy, in healthy livers, shown in a paired analysis with connecting lines representing matched samples. The highest number of *in situ* IFN-γ–producing cells was observed among CXCR6+NK cells, followed by CXCR6+NKT cells, CXCR6+ CD3+ T cells, and, lastly, CXCR6- CD3+ T cells. CXCR6+NK cells, CXCR6-positive natural killer cells; HBV, hepatitis B virus; IFN-γ, interferon-gamma; NUC, nucleos(t)ide analog; MFI, mean fluorescence intensity;.

To investigate whether lymphocytes produce IFN-γ under steady-state conditions in the human liver, we first examined the Human Protein Atlas,^18^ which shows IFN-γ expression in healthy liver tissue but does not define the cellular source at the protein level. We therefore performed multiplex immunofluorescence on core needle biopsies from healthy donors (n = 7; Table S1), using diagnostic pathology-grade antibody panels to detect IFN-γ protein, CD3, CD56, CXCR6, and nuclear DAPI staining. An illustrative example is shown in Fig. 1A; additional illustrative overlays highlighting IFN-γ protein expression in CD56+ CXCR6+ CD3- NK cells are provided in Fig. S1C. Detailed staining overlays and machine learning-based cell selection workflow, including cell count validation, are provided in Figs. S1 and S2. This approach enabled spatial visualization of cytokine expression and cell phenotype within intact liver architecture, without the confounding effects of *ex vivo* stimulation. IFN-γ–producing lymphocytes were detected in both parenchymal and portal regions (Fig. 1B) and were uniformly CD3^-^, CD56+, and CXCR6+, consistent with liver-resident NK cells.^11^ For clarity, CD56^-^ cells were not annotated in Fig. 1B, as they did not express IFN-γ above background, allowing a clearer view of the spatial distribution of IFN-γ–producing cells. Quantitative image analysis using AI-based tools, with mean fluorescence intensity (MFI) thresholds determined individually for each marker (see Supplementary Methods), confirmed that CXCR6+NK cells were the dominant *in situ* IFN-γ producers in healthy liver, with a median of 24.2% (IQR: 14.1–67.2%) expressing detectable protein. Comparison of absolute counts within each individual tissue section confirmed these results (Fig. 1C): CXCR6+NK cells were the most abundant IFN-γ–producing population (median 1,269 cells; IQR: 696–2,904), followed by CXCR6+NKT cells (median 94; IQR: 72–116.5), CXCR6+ CD3+ T cells (median 3; IQR: 1.5–4.5), and CD3+CD56^-^CXCR6^-^ T cells (median 2; IQR: 1–2.5). CD56+CXCR6^-^ NK cells did not express IFN-γ above the background threshold. These data identify CXCR6+NK cells as the principal source of *in situ* IFN-γ in the healthy human liver.

### Liver IFN-γ protein expression is not restored in NUC-treated HBV

**Fig. 2:**
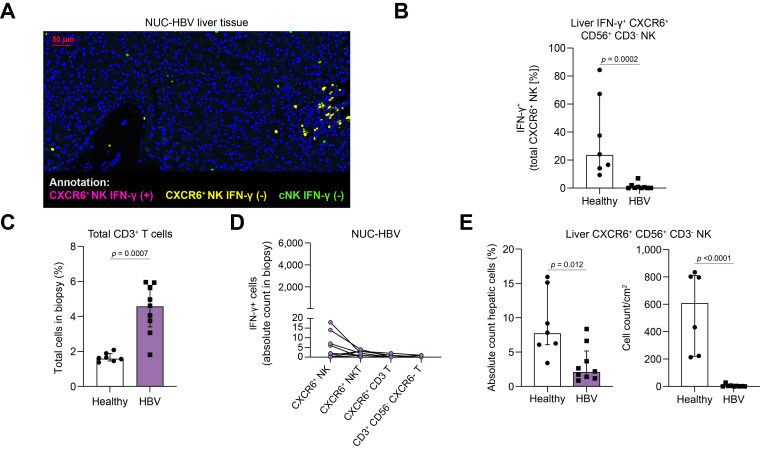
Intrahepatic IFN‑γ production by CXCR6+NK cells is lost in NUC‑HBV (A) Algorithmic phenotyping of immune cells in NUC-HBV liver tissue, staining of IFN-γ+ CXCR6+NK cells in a healthy liver sample, using antibodies against CD56, CD3, CXCR6, IFN-γ, and nuclear staining with DAPI. (B) Frequency (%) of IFN-γ+ CXCR6+ CD56+ CD3- NK cells as a proportion of total CXCR6+NK cells in healthy and NUC-HBV liver biopsies. (C) Total CD3+ T-cell counts per biopsy in healthy controls and NUC-treated patients with HBV. (D) Absolute number of IFN-γ+ cells per biopsy, in NUC-HBV livers, shown in a paired analysis with connecting lines representing matched samples. (E) Frequency (%) of total detected cells and absolute density (cells per cm^2^ of tissue) of CXCR6+ CD56+CD3- NK cells, compared between patients with NUC-HBV and healthy controls. Data are shown as median with interquartile range. Comparisons between healthy and NUC-HBV samples were performed using a two-tailed Mann--Whitney *U* test; *p* <0.05 was considered significant. Abbreviations: CXCR6+NK cells, CXCR6-positive natural killer cells; HBV, hepatitis B virus; IFN-γ, interferon-gamma; NUCs, nucleos(t)ide analogs.

To test the longstanding hypothesis that intrahepatic IFN-γ production is impaired in chronic HBV, we focused on patients receiving long-term NUC therapy. This clinical context minimizes inflammation-related immune activation and offers the closest approximation to healthy liver among HBV-infected individuals. It therefore provides a stable background to assess IFN-γ production without confounding interference from ongoing liver injury or viral replication. We analyzed liver biopsies from NUC-treated patients with HBV (n = 9), all with normal alanine aminotransferase (ALT) and no histological fibrosis (Table S1). Detailed staining images and absolute immune cell counts are provided in Fig. S2 and Fig. S1B, respectively. Multiplex immunofluorescence showed that CXCR6+NK cells in NUC-HBV samples rarely expressed IFN-γ protein (Fig. 2A; Fig. S2), with only 0.2% (IQR: 0.0–1.4%) staining positive (Fig. 2B). Despite a significant increase in CD3+ T-cell frequency in NUC-HBV livers (median 5.6%; IQR: 3.4–5.8) compared with healthy controls (median 1.6%; IQR: 1.5–1.9; *p* = 0.0007; Fig. 2C), these cells did not compensate for the loss of IFN-γ production. No other CD3+ or CD56+ subset contributed meaningfully to *in situ* IFN-γ expression (Fig. 2D).

The total number of CXCR6+NK cells was also reduced in NUC-HBV livers (median 675 cells; IQR: 704.5) compared with healthy controls (median 1,918; IQR: 5,751), with their frequency among total liver cells declining from 7.8% to 2.2% (*p* = 0.012) (Fig. 2E).

These data show that the reduced intrahepatic IFN-γ signal in NUC-HBV is caused by both a lower proportion of IFN-γ+ CXCR6+NK cells and a numerical loss of this population. This supports the hypothesis that IFN-γ production is impaired in chronic HBV, even under conditions of effective viral suppression and absent inflammation.

### Liver scRNAseq confirms depletion and loss of *IFNG* and cytokine gene expression in CXCR6+NK cells in NUC-HBV

**Fig. 3:**
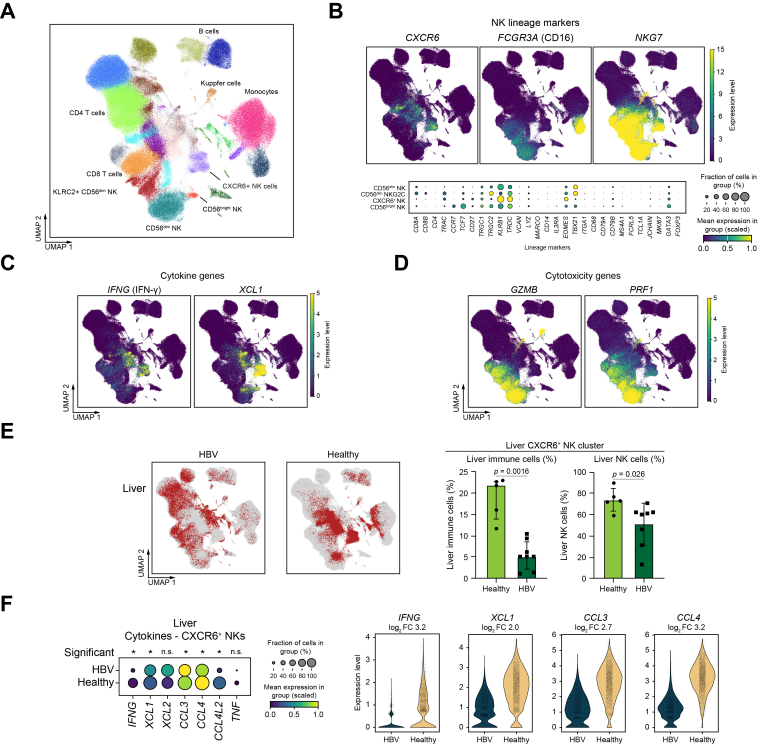
scRNA‑seq identifies a liver‑restricted CXCR6+NK cell cluster with selective loss of cytokine gene expression in NUC‑HBV (A) Uniform Manifold Approximation and Projection of immune subsets in liver and blood, with NK cell clusters highlighted. (B) Feature plots showing NK markers (*CXCR6, FCGR3A, NKG7*). (C, D) Cytokine (*IFNG, XCL1*) and cytotoxicity (*GZMB, PRF1*) genes in liver NK cells. (E) Left: UMAP plot depicting single-cell distributions from HBV and healthy liver tissues. Cells from HBV or healthy samples are highlighted in red, with all other cells shown in grey. The black circle outlines the cluster corresponding to CXCR6+NK cells; dots within this circle represent cells belonging specifically to this cluster. Right: Frequency of liver CXCR6+NK cells (cluster 13) in healthy and NUC-HBV, represented as a percentage of total liver immune cells and as total liver NK cells. (F) Left: Dotplot showing expression of differentially expressed cytokine genes in liver CXCR6+NK cells, scaled by mean expression for each group. Right: Violin plots highlighting the top significant cytokines, such as *IFNG* and *XCL1*. Frequencies are shown as median with interquartile range. Statistical comparisons between healthy and NUC-HBV liver CXCR6+NK cell frequency were performed using a two-tailed Mann-Whitney *U* test; *p* <0.05 was considered significant. Genes shown were identified as differentially expressed using a two-tailed Wilcoxon rank-sum test with Benjamini–Hochberg correction for multiple comparisons. Adjusted *p* <0.05 was considered significant. Abbreviations: CXCR6+NK cells, CXCR6‑positive natural killer cells; HBV, hepatitis B virus; IFNG, interferon‑gamma gene; XCL1, X‑C motif chemokine ligand 1; GZMB, granzyme B; PRF1, perforin 1; NUC, nucleos(t)ide analog.

To gain deeper insight into the transcriptional programs underlying the loss of IFN-γ in NUC-treated HBV liver, we performed scRNA-seq on liver FNA and blood samples from 18 patients who were HBeAg-negative with chronic HBV on long-term NUC therapy (median duration: 7 years), all with normal ALT and minimal fibrosis (F0–F1). Fig. S3 shows longitudinal HBV DNA and ALT measurements over up to 10 years, confirming durable viral suppression. For scRNAseq analyses, nine patients contributed paired liver and blood samples, with one liver sample excluded as a result of quality control. Publicly available scRNA-seq datasets from control livers (n = 5) and healthy PBMCs (n = 9) were integrated for comparison. Table S2 provides detailed information on sample origin (liver/PBMC), clinical data, and Gene Expression Omnibus accession numbers. After quality control and batch correction, 221,383 immune cells were retained for analysis (Fig. S4 and Supplementary Methods). Unsupervised clustering identified major innate and adaptive immune populations, including four distinct NK cell clusters. Fig. S4 provides detailed QC metrics, data integration performance, and cluster-level annotations. Among these, a single CXCR6+NK cell cluster was identified, exclusive to liver samples, and representing the dominant hepatic NK-cell population. This cluster was transcriptionally defined by high expression of cytokine-related genes including *IFNG*, *TNF*, *XCL1*, C–C motif chemokine ligand 3 (*CCL3*), and C–C motif chemokine ligand 4 (*CCL4*), and relatively lower levels of cytotoxic effectors such as *GZMB* and *PRF1*, distinguishing it from circulating CD56dim and CD56bright NK cells (Fig. 3A–D).

Consistent with our *in situ* data, CXCR6+NK cells were less frequent in NUC-HBV livers than in control liver. Although numerical comparisons should be interpreted with caution given potential differences in tissue sampling (FNAs *vs.* surgical resections), the observed reduction was evident both as a fraction of total immune cells (median 5% *vs.* 22%, *p* = 0.002) and within the NK compartment (61% *vs.* 73%, *p* = 0.026) (Fig. 3E).

Differential gene expression analysis, correcting for potential numerical and batch differences between datasets, showed that this liver-restricted CXCR6+NK cell cluster exhibited marked downregulation of *IFNG*, directly confirming the loss of IFN-γ observed at the protein level (Fig. 3F). Other effector genes linked to dendritic cell recruitment—including *XCL1*, *CCL3*, *CCL4*, and *CCL4L2*—were also reduced, indicating a broader impairment in immunoregulatory output. In contrast, expression of key inflammatory cytokines such as *TNF* and chemokines including *CCL5* and *XCL2* remained intact (Fig. 3F). Together, these findings demonstrate that in NUC-HBV liver, CXCR6+NK cells undergo a selective and specific downregulation of cytokine programs, particularly *IFNG* and chemokine genes.

### Liver CXCR6+NK cells retain cytotoxicity gene expression despite cytokine suppression

**Fig. 4:**
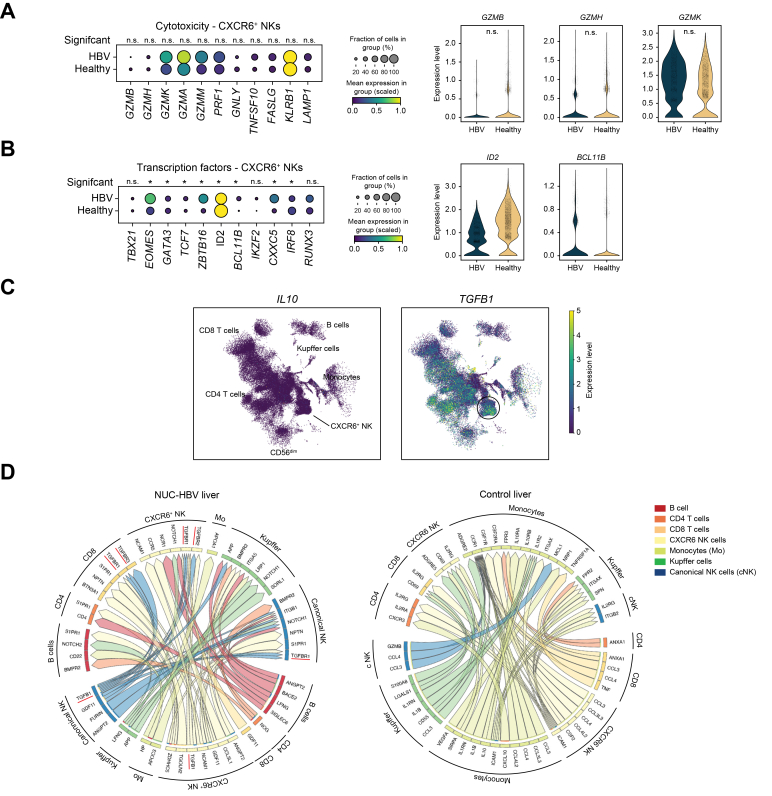
Liver CXCR6+NK cells in NUC‑HBV retain cytotoxic programs but show altered transcription factor and TGF‑β–associated signaling (A) Left: Dotplot showing comparable expression of cytotoxicity-related genes (*e.g. GZMB*, *PRF1*) in CXCR6+NK cells across groups. Right: Violin plots displaying expression of key granzyme genes. (B) Left: Dotplot illustrating transcription factor expression (e.g., *ZBTB16, BCL11B*) in CXCR6+NK cells. Right: Violin plots showing significant transcription factors in HBV-affected NK cells. (C) Feature plots from liver *IL10* and *TGFB1* expression. (D) Top 100 receptor-ligand interactions within healthy and NUC-HBV liver with *TGFB1* underlined in red. Genes shown in panels (A and B) were identified as differentially expressed using a two-tailed Wilcoxon rank-sum test with Benjamini–Hochberg correction for multiple comparisons. Adjusted *p* <0.05 was considered significant. Color scale: yellow = increased expression; purple/blue = decreased expression.Abbreviations: CXCR6⁺NK cells, CXCR6‑positive natural killer cells; HBV, hepatitis B virus; NUC, nucleos(t)ide analog; GZMB, granzyme B; PRF1, perforin 1; ZBTB16, zinc finger and BTB domain‑containing protein 16; BCL11B, B‑cell leukemia/lymphoma 11B; IL10, interleukin‑10; TGFB1, transforming growth factor beta 1.

Given the profound cytokine suppression at the transcriptional level, we next examined whether cytotoxicity-associated genes were similarly affected. Expression of *GZMB*, *GZMK*, *FCGR3A*, *FASLG*, and *TNFSF10* remained unchanged in CXCR6+NK cells (Fig. 4A), indicating that the transcriptional changes are selective for *IFNG* and chemokines and do not extend to cytotoxicity-related genes. In contrast, several differentially expressed genes in NUC-HBV CXCR6+NK cells were transcription factors known to regulate NK cell development and effector function. *ID2* was markedly downregulated, whereas *BCL11B* and *ZBTB16* were upregulated in NUC-HBV (Fig. 4B). Given the significant regulatory impact of transcription factors, we also assessed subtle expression changes (fold change [FC] <2). This revealed downregulation of *GATA3* (FC −1.40) and upregulation of *TCF7* (FC 1.29) and *EOMES* (FC 1.61). Upregulation of TCF7, alongside downregulation of ID2, reflects a transcriptional program indicative of less mature NK cells with reduced capacity for IFN-γ production, whereas the upregulation of BCL11B and ZBTB16 is consistent with preserved cytotoxic potential.[19], [20], [21], [22], [23]

### Liver immune signaling shifts toward TGF-β–associated pathways in NUC-treated HBV

Next, we asked whether the observed transcriptional cytokine defect in CXCR6+NK cells reflects broader regulatory shifts in the intrahepatic immune environment. We first examined expression of key immunoregulatory genes and found that *TGFB1* was broadly expressed across multiple immune clusters, including NK cells, whereas *IL10* expression was negligible (Fig. 4C). To systematically explore intercellular communication in the scRNAseq data (Table S2), we performed cell–cell interaction analysis using major intrahepatic immune populations. Compared with control liver, NUC-HBV samples showed a marked reduction in predicted pro-inflammatory cytokine interactions (Fig. 4D). Among the top 100 predicted ligand–receptor pairs, only one involved inflammatory signaling (*CCL3L1–CCR5*). In contrast, TGF-β–associated signaling emerged as a dominant feature in NUC-HBV liver. *TGFB1* was expressed by multiple immune subsets—including NK cells—and predicted to signal through *TGFBR1* and *TGFBR2* on both CD8 T cells and NK cells. Control liver samples exhibited more extensive pro-inflammatory crosstalk, largely driven by CXCR6+NK cell–derived chemokines such as *CCL3* and *CCL4*. In contrast, TGF-β–related interactions were not predicted in control liver. Together, these findings suggest that the immune environment in NUC-HBV liver is characterized by reduced cytokine network activity and a shift toward regulatory signaling, particularly involving TGF-β. This may contribute to the selective suppression of IFN-γ production in liver-resident NK cells.

### Liver-restricted loss of IFN-γ–producing CXCR6+NK cells is not observed in blood

To determine whether the reduction in CXCR6+NK cells and *IFNG* expression extends beyond the liver, we analyzed the scRNAseq dataset (Table S2) and an additional PBMC cohort (Table S3). In the scRNAseq dataset, no CXCR6+ liver-resident NK cell cluster was detected in blood (Fig. S5A). Circulating NK populations, including CD56dim and CD56bright clusters, showed no reduction in *IFNG* expression or consistent changes in cytokine gene expression between NUC-HBV (n = 18) and control blood (n = 9). Flow cytometry on the PBMC cohort (NUC-HBV, n = 44; healthy controls, n = 10; Table S3) confirmed that CXCR6+ cells were rare within the CD56brightNK compartment in blood and present at similar frequencies in both groups (Fig. S5B and C). These data indicate that the loss of IFN-γ–producing CXCR6+NK cells is confined to the liver and is not a systemic feature of NUC-treated chronic HBV.

### Liver CXCR6+NK cell loss correlates with HBsAg levels and persists despite long-term NUC therapy

**Fig. 5:**
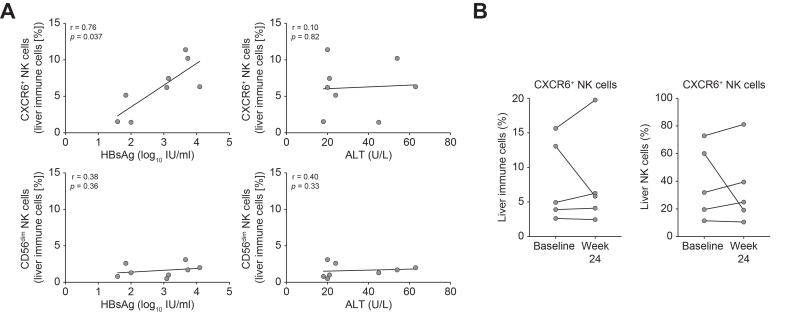
Loss of CXCR6+NK cells correlates with HBsAg levels and is not restored during NUC therapy (A) Correlation plots showing the relationship between serum HBsAg levels (log_10_ IU/ml), ALT (U/L), and the frequency of CXCR6+NK cells (cluster 13) and CD56dim NK cells (cluster 4) as a percentage of total liver immune cells in FNAs from patients on long-term NUC therapy (n = 8). A full correlation matrix is provided in Table S4. Cohort details: Table S2. Spearman’s rank correlation was used for correlation analysis, which does not assume normal distribution. Spearman R and two-tailed *p* values are reported; *p* <0.05 was considered significant. (B) Frequency of CXCR6+NK cells in serial liver FNAs during 24 weeks of NUC therapy, displayed both as percentage of total liver immune cells and percentage of total liver NK cells (cohort details: Nkongolo *et al.*^24^). Statistical comparisons between baseline and week 24 were performed using a two-tailed Wilcoxon signed-rank test; *p* <0.05 was considered significant. ALT, alanine aminotransferase; CXCR6+NK cells, CXCR6-positive natural killer cells; FNA, fine-needle aspiration; NUC, nucleos(t)ide analog.

To explore potential clinical factors associated with reduced CXCR6+NK cell frequency, we analyzed the correlation between the NK cell clusters and clinical parameters within the scRNAseq NUC-HBV cohort (Table S2). No associations were observed with age, ALT, or treatment duration (Table S4). However, CXCR6+NK cell cluster frequency strongly correlated with serum HBsAg levels (Spearman R = 0.76, *p* = 0.037; Fig. 5A), suggesting a link between persistent HBsAg exposure and the intrahepatic loss of these cells.

Finally, we addressed whether the observed reduction in CXCR6+NK cells and their loss of *IFNG* expression reflects a general feature of chronic HBV infection or a specific consequence of NUC therapy. We reanalyzed a published longitudinal scRNAseq dataset of patients with HBV sampled before and after NUC initiation.^24^ At baseline, these patients exhibited active liver disease with high HBV DNA and elevated ALT, both of which decreased upon treatment, while HBsAg levels remained stable. As shown in Fig. 5B, the single CXCR6+NK cell cluster present at baseline remained low in both frequency and IFNG expression over 24 weeks of NUC therapy. Fig. S6 provides detailed clustering, liver NK cell frequencies, and expression levels of key NK-cell cytokines and cytotoxic factors. Transcriptomic analysis confirmed stable expression of cytotoxicity genes and key transcription factors, with no recovery in cytokine genes such as *IFNG*, *XCL1*, *CCL3*, or *CCL4*. These findings support that the impaired cytokine profile of CXCR6+NK cells is not reversed by 24 weeks NUC therapy and may represent a feature of chronic HBV infection.

## Discussion

Chronic HBV infection under NUC therapy is widely regarded as a state of sustained virological and biochemical control, yet our findings reveal a persistent deficit in intrahepatic immunity: the near-complete absence of *in situ* IFN-γ production by liver-resident CXCR6+NK cells obtained from patients with NUC-HBV. Using immunofluorescence on well-characterized archived liver biopsies, we identified CXCR6+NKs as the dominant source of IFN-γ in healthy liver. In contrast, NUC-treated HBV livers lacked not only IFN-γ–producing CXCR6+NK cells, but also any compensatory production by CD3+ T cells or other NK immune subsets. This establishes CXCR6+NK cells as a critical, non-redundant source of intrahepatic IFN-γ.

ScRNAseq confirmed and extended these findings. CXCR6+NK cells, exclusive to the liver, displayed the highest cytokine gene expression in healthy controls, yet were numerically and transcriptionally suppressed in NUC-HBV livers. Although cytotoxicity-related genes were preserved, the hallmark effector cytokines *IFNG* and chemokines (*e.g. XCL1* and *CCL4*) were profoundly downregulated. No similar changes were observed in circulating NK cells, underscoring that this cytokine defect is liver-restricted. In contrast to CD8+ T cells, which have been reported to show dynamic restoration of function during viral suppression,^24^ CXCR6+NK cells remain in a low IFN-γ–producing state. Analysis of untreated patients with HBV before and after NUC initiation revealed no recovery in CXCR6+NK cell frequency or cytokine expression, suggesting that this pattern is established early in infection and persists regardless of virological control or inflammation. These findings raise questions about the mechanisms sustaining this loss of cytokine production. A notable correlation between CXCR6+NK cell frequency and HBsAg levels, but not ALT or treatment duration, hints at persistent antigenemia (*e.g.* HBsAg) as a potential driver of *in situ* loss of cytokine production. Cell–cell interaction analyses further to a regulatory intrahepatic immune landscape in NUC-HBV, with loss of pro-inflammatory cytokine networks and TGF-β signaling—a known suppressor of NK cell function.^25^

Together, these data suggest that the loss of specific CXCR6+NK cell cytokines contributes to a broader attenuated immune state in HBV infection, and that the loss of cytokine producing CXCR6+NK cells may represent a bottleneck to immune restoration. Whether this reflects an irreversible epigenetic imprint, antigen-driven selection, or autocrine TGF-β feedback remains unclear. It is important to note that exhaustion-related gene programs (*e.g. PDCD1*, *HAVCR2*, *TOX*, *TOX2*, *CD274,* and *LAG3*), which have been suggested to be modulated during chronic viral stimulation^26^ and shown to influence *IFNG* expression, were expressed at levels comparable with those in control liver (Fig. S7). Notably, murine models show that NK cells can release TGF-β1 in response to apoptotic cells, and that autocrine TGF-β is required for NK cell maintenance.^25^^,^^27^ Similar pathways may operate in HBV-infected liver, but require further investigation. Our data align with recent observations in HBV-specific CD8+ T cells, where TGF-β dampens antiviral responses without classical exhaustion.^28^ IFN-γ secretion by NK cells relies on cytokine priming and dendritic cell (DC) interactions, particularly through IL-12, IL-15, and IL-18, whereas cytotoxic function is maintained.^29^ The observed loss of DC-attracting cytokines from CXCR6+NK cells suggests that their decline may further limit the recruitment and activation of DCs, potentially leading to a self-reinforcing loop of reduced immune activation in the HBV-infected liver. Restoring NK cell–derived IFN-γ may thus be a key strategy to reactivate intrahepatic immunity. Therapeutic avenues could include TLR8 agonists, IFN-α–based therapies, or novel vaccination platforms aimed at restoring IFN-γ cytokine production.

Our study has limitations. We performed FNA on patients with NUC-HBV, but comparison with healthy liver counterparts is notoriously difficult for obvious ethical reasons. Therefore, we first performed multiplex immunofluorescence on archived liver biopsies from healthy donors to establish baseline *in situ* IFN-γ expression, and then confirmed these findings using scRNAseq datasets generated from FNAs and biopsies. Although the control liver scRNAseq dataset we used is widely accepted as representing healthy liver, it was derived from patients undergoing resection for colorectal liver metastases.^15^ Moreover, integrating datasets is essential to address the major challenge of obtaining truly healthy liver scRNAseq data, yet differences in sample origin, processing protocols, and 10 × Genomics versions may influence the expression of sensitive transcripts such as cytokines. To address this, we performed rigorous batch correction and confirmed robust dataset integration (Fig. S2). Nevertheless, we cannot fully exclude that these experimental differences have influenced the transcriptional profiles. Importantly, the scRNAseq data showed a selective loss of *IFNG* with preserved expression of other effector genes such as *TNF* and *XCL2*. This concordance across independent modalities strongly supports the validity of our conclusions.

In conclusion, our data support the longstanding hypothesis that intrahepatic IFN-γ production is impaired in NUC-treated chronic HBV infection. This deficit is specifically localized to CXCR6+ liver-resident NK cells, which are reduced in number and show consistently low protein and gene expression of IFN-γ, despite long-term viral suppression. Unlike CD8+ T cells, which may recover function during NUC, CXCR6+NK cells exhibit a stable reduction in IFN-γ and chemokine gene associated activity, even in the absence of inflammation. This intrahepatic loss of IFN-γ is not observed in peripheral NK cells and coincides with reduced pro-inflammatory cytokine interactions and increased TGF-β–associated signaling. These findings suggest a tissue-specific immune adaptation, potentially shaped by chronic antigen exposure (*e.g.* HBsAg) and local regulatory signals, that limits *in situ* IFN-γ production in CXCR6+NK cells. Targeting this bottleneck by restoring CXCR6+NK cell function or compensating for their reduced IFN-γ output, may be essential to achieving immune control and advancing HBV cure strategies.

## Abbreviations

ALT, alanine aminotransferase; cccDNA, covalently closed circular DNA; CCL3, C–C motif chemokine ligand 3; CCL4, C–C motif chemokine ligand 4; CXCR6+NK cells, CXCR6-positive natural killer cells; FASLG, Fas ligand gene; FNA, fine-needle aspiration; GZMB, granzyme B; HCC, hepatocellular carcinoma; IFN-γ, interferon-gamma; *IFNG*, interferon-gamma gene; ISGs, interferon-stimulated genes; NK, natural killer; NUCs, nucleos(t)ide analogs; PBMCs, peripheral blood mononuclear cells; PLZF, promyelocytic leukemia zinc finger; PRF1, perforin 1 gene; scRNAseq, single-cell RNA sequencing; TBX21, T-box transcription factor 21; TGF-β, transforming growth factor-beta; TNFSF10, tumor necrosis factor superfamily member 10 gene; XCL1, chemokine (C-X-C motif) ligand 1.

## Authors’ contributions

Conceptualized the study, wrote and performed analysis: BJBB. Performed bioinformatic analysis: DAK. Supervised the project: AB. Provided data: GWvO, ZMAG, KAL, AJG, TvdB, SN. Supervised bioinformatic analysis: HJGvdW. Clinical supervisor: RJdK.

## Data availability

Data are publicly available and has been deposited in Gene Expression Omnibus (https://www.ncbi.nlm.nih.gov/geo/) with accession number: GSE247322. Multiplex immunofluorescence data are available from the corresponding author for academic, non-commercial purposes aimed at supporting transparency, reproducibility, or further research. Data will be shared in accordance with ethical approvals and institutional guidelines.

## Financial support

10.13039/501100015383The Foundation for Liver and Gastrointestinal Research (SLO) sponsored the study. The funding source did not influence the study design, data collection, analysis and interpretation of the data, writing of the report, or the decision to submit for publication.

## Conflicts of interest

The authors declare no conflicts of interest.

Please refer to the accompanying ICMJE disclosure forms for further details.
