# Supplementary material for: Persistent loss of intrahepatic IFN-γ in HBV is linked to selective impairment of liver-resident CXCR6+NK cells despite long-term NUC therapy
Source: JHEP Rep. 2026 Apr 18;8(7):101865. doi: 10.1016/j.jhepr.2026.101865 (PMC13264066; doi:10.1016/j.jhepr.2026.101865)
Supplement: Multimedia component 1 [file mmc1.pdf]

Persistent loss of intrahepatic IFN-γ in HBV is linked to selective impairment of liver-resident CXCR6+NK cells despite long-term NUC therapy

Boris J B Beudeker, Diren Arda Karaoglu, Shirin Nkongolo, Gertine W van Oord, Zwier MA Groothuismink, Karishma A Lila, Adam J. Gehring, Thierry van den Bosch, Robert J de Knegt, Harmen JG van de Werken, Andre Boonstra

Table of contents

Supplementary methods..2

Supplementary figures..10

Supplementary tables..19

Supplementary methods

Study population

Chronic HBV patients visiting the outpatient clinic of Erasmus MC (Rotterdam, the Netherlands) were enrolled in this study. Included were HBeAg-negative adults (≥18 years) who had achieved stable viral control with entecavir or tenofovir (HBV DNA <80 IU/mL) for >3 years. Exclusion criteria were liver fibrosis (elastography >7.0 kPa or Metavir >F1), history of hepatic decompensation or hepatocellular carcinoma, co-infection with HCV, HDV, HEV, or HIV, autoimmune liver disease, severe steatosis or steatohepatitis, hemochromatosis, Wilson's disease, documented alcohol abuse, malignancy, treatment with vitamin K antagonists, or recent pregnancy. Clinical data were obtained from electronic medical records. Fibrosis was assessed by Fibroscan® or liver histology. Serum HBsAg levels (IU/mL) were measured with the Lumipulse G HBsAg assay (Fujirebio Europe) on a LUMIPULSE G1200 analyzer (Fujirebio Inc.).

Liver control samples were obtained from Ramachandran et al. (GSE136103), consisting of non-lesional tissue resected during surgery for solitary colorectal metastases. These individuals had no liver disease, normal biochemistry, and had not received neoadjuvant chemotherapy. Histological review confirmed absence of fibrosis or pathological alterations, making these samples widely accepted references for control liver tissue.

All participants provided written informed consent. The study was approved by the Erasmus MC Medical Ethics Committee.

Human peripheral blood mononuclear cells and fine-needle aspirates of the liver

At the outpatient clinic, heparinized peripheral blood and ultrasound-guided liver fine-needle aspirates (FNAs) were collected. PBMCs were isolated by Ficoll-Paque™ Plus (GE Healthcare Bio-Sciences AB), cryopreserved in FCS with 10% DMSO, and stored in vapor-phase liquid nitrogen at –150°C.

Intrahepatic leukocytes were obtained by FNA, a minimally invasive approach that has only been applied in a few single-cell studies to date. To minimize artifacts such as blood carryover or ischemia-related transcriptional changes, we implemented a validated rapid pipeline (reference 5) with immediate transport on ice and processing to single-cell droplet encapsulation in <1 hour. Compared with approaches that enrich CD45+ cells or use larger 20-gauge needles, our protocol

employs a finer 25-gauge Spinocan® needle, which yields cleaner aspirates enriched for intrahepatic leukocytes and minimizes red blood cell contamination.

For scRNA-seq, four passes were obtained per patient and collected in 500 µL colorless RPMI. Sample quality was assessed by optical density at 415–595 nm, with OD ≥0.19 indicating contamination by intrahepatic blood; such samples were excluded. Passes passing QC were pooled. Residual RBCs were depleted by incubation with Red Blood Cell Removal Solution (Stemcell) for 5 minutes, followed by two washes. Cells were counted, adjusted to 700–1200 cells/µL in RPMI + 5% FCS, and subjected immediately to single-cell encapsulation.

This validated workflow (5) ensures rapid and reproducible sampling of intrahepatic immune cells, providing high-quality material distinct from datasets generated by CD45+ sorting or large-bore biopsy approaches.

FFPE biopsies

Archived FFPE core liver biopsies archived at Erasmus MC were selected from HBV patients who had achieved successful long-term viral suppression on NUC therapy. These included biopsies obtained as study endpoints for NUC-related trials or at the initiation of NUC cessation. For comparison, liver biopsies from eligible altruistic healthy liver donors, collected to assess their eligibility as donors, were included. All tissue was processed in the same pathology facility to ensure consistent handling.

Phenotyping by flowcytometry

PBMC were thawed and washed with RPMI 1640 supplemented with 10% fetal calf serum (Lonza,). For flowcytometry, 250 000 viable PBMC were stained with antibodies against PD1-PerCP-eFluor710 (eBioJ105; eBioscience); TIM-3-PerCP (344823; R&D); CD56-APC vio770 (REA196; Miltenyi); TRAIL-Alexa Fluor488 (75402; R&D); CXCR6-APC (K041E5; Biolegend); TIGIT-BV786 (741182; BD Bioscience); KLRG1-PE (SA231A2; Biolegend); CD3-PEeFluor610 (UCHT1; eBioscience); HLA-DR-PE-Cy5 (LN3; eBioscience), CD38-PeCy7 (HB7; eBioscience), and viability-BV570 (Miltenyi); for 20 minutes at 4°C in the dark with the desired mixture of antibodies. Data were analyzed using FlowJo version 10.1 (Tree Star Inc.).

Sample preparation and scRNAseq on the 10x Genomics platform

Samples were prepared according to the 10x Genomics Single Cell 3' and 5' Reagent Kit user guide. Briefly, the maximum volume was loaded on a 10x Genomics Chromium to target capturing a maximum of 10,000 cells. After droplet generation, samples were transferred into a pre-chilled tube strip and cDNA was generated. The next day, cDNA was recovered using Recovery Agent provided by 10x Genomics and subsequently purified using a Silane DynaBead mix (Thermo Fisher) as outlined by the user guide. Purified cDNA was amplified for 13 cycles before being purified again using SPRIselect beads (Beckman Coulter). cDNA concentrations of the samples were determined on a Bioanalyzer (Agilent Technologies). Libraries were prepared as outlined by the 10x Genomics' Single Cell 3' and 5' Reagent Kit user guide, and sequenced (28-8-0-91 cycles) on a Novaseq6000 platform (Illumina).

Analysis of scRNAseq data

scRNAseq data from PBMC and liver samples were obtained as described above or obtained from publicly available datasets. For these public datasets, raw FASTQ files were used when available; otherwise, preprocessed H5AD files were utilized. For Steele et al. (1), data were retrieved from GEO (GSE157789), and metadata were obtained from the supplementary files of the manuscript. Similarly, for Sinha et al. (2), data were downloaded from GEO (GSE155698), with metadata sourced from the supplementary materials. For Ramachandran et al. (3), GEO (GSE136103) provided the raw data, while supplementary files from the manuscript and personal inquiry were used for metadata.

Raw FASTQ files were processed into count matrices using 10x Genomics Cell Ranger (v6.1.2) (4) with the human reference genome GRCh38 and default settings. To ensure a high-quality dataset, comprehensive quality control (QC) measures were applied. Cells were flagged as outliers if they deviated by more than 5 median absolute deviations (MADs) from the median for log-transformed total counts, log-transformed number of genes detected, or the percentage of counts in the top 20 most highly expressed genes. Cells with mitochondrial gene expression levels exceeding 3 MADs from the median or greater than 20% were also removed. These QC steps were performed following single-cell best practices (5). Ambient RNA contamination was corrected using SoupX (6). Cells expressing fewer than 200 genes and genes detected in fewer than three cells were filtered out using scanpy's filter_cells and filter_genes functions, respectively (7). After QC filtering, samples were integrated into a single dataset. Doublets were identified and flagged

using scDblFinder (8), ensuring a dataset with single-cell resolution. Out of 19 blood samples and 9 FNAs, one liver sample did not pass quality control and was excluded due to low-quality cells as described by the criteria above. Single-cell clustering was performed on 19 chronic hepatitis B infection PBMC samples, 9 healthy PBMC controls, 8 chronic hepatitis B infection fine-needle liver aspirates, and 5 healthy liver resections were included, retaining 221,383 cells for downstream analyses. Table S2 shows individual characteristics of our scRNAseq cohort.

Normalization was performed using scran (9), and highly variable genes were selected for subsequent steps. Dimensionality reduction using UMAP and batch effect correction were conducted with scVI (10). Clustering was performed using the Louvain algorithm at a resolution of 1.0, which identified 29 distinct clusters (Supplementary Data Table 2, Fig. S4A & H). This resolution was chosen to balance granularity and interpretability, allowing for the detection of biologically relevant immune subpopulations, including smaller subsets such as FOXP3+ CD4+ T cells and distinct NK cell subsets, without over-fragmenting known immune cell lineages (Fig. S4C).

To ensure robust immune cell classification, we performed reclustering (Fig. S4F), which did not result in the identification of additional NK cell subpopulations beyond those initially detected. Furthermore, we observed that all liver immune cell populations were present in both healthy and NUC-treated HBV samples, confirming a shared immune landscape across conditions (Fig. S4 D & H).

Marker genes for each cluster were determined using scanpy's rank_genes_groups function with Benjamini-Hochberg correction applied for multiple comparisons (gene list for annotation and cell counts in Fig. S4C and supplementary data table 1). Marker genes for each cluster were determined using scanpy's rank_genes_groups function with Benjamini-Hochberg correction applied for multiple comparisons.

Natural killer (NK) cells were identified based on essential markers and absence of T cell genes such as *CD3D*, *CD3G*, *CD4*, and *TRA*C, they included: CD56dim (Cluster 4): expressing transcription factor *TBX21* and chemokine receptor *CX3CR1+,* alongside ADCC and cytolytic factors (*FCGR3A+, GZMB+, PRF1+, GNLY, GZMA*), inhibitory markers (*HAVCR2* [TIM-3], KIRs). KLRC2+ CD56dim (Cluster 6): sharing largely overlapping differentially expressed genes with Cluster 4, but also expressing KLRC2, *TIGIT, KLRG1, LAG3,* and T-cell receptor-related genes (*TRGC2, CD3E* without other *CD3 genes*). CXCR6+ (Cluster 13): defined by the chemokine

receptor *CXCR6+,* activation marker *CD69,* transcription factor *EOMES,* abundant cytokine gene expression (*IFNG, TNF, XCL1, XCL2, CCL3, CCL4, CCL4L2, CCL5*), cytotoxic factors (*GZMK, GZMA, TNFSF10* [TRAIL]), and inhibitory factors (*KLRC1, TOX2, TIGIT*). CD56bright (Cluster 22): characterized by *NCAM1+, EOMES+, GZMK+, IL2RB+, KLRC1+, CD44+, SELL+*(Fig. S4, supplementary data table 1). This workflow provided a high-quality, well-annotated dataset for downstream analysis of. Differential gene expression analysis was performed to identify transcriptional shifts in NK clusters (Supplementary Data Table 2), focusing on biologically relevant changes (FC >2, adj. p <0.05). In liver, CXCR6+NK cells (Cluster 13) showed 83 DEGs, including downregulation of *IFNG*, *XCL1*, *CCL3*, *CCL4*, and upregulation of transcription factors (*TCF7, BCL11B, ZBTB16*). CD56dim NK cells (Cluster 4) displayed 74 DEGs, with overlap in *ID2* and cytotoxic genes (*GZMH, GZMK*), but not cytokines. In blood NK clusters, only minor shifts were observed (<20 DEGs per subset), with no loss of *IFNG*. Thus, major transcriptional alterations were liver-restricted. Cell-cell interaction analysis was conducted using MultiNicheNetR v2.0 (11). This type of analysis (Multinichenet) considers receptor-ligand pairs that are more strongly expressed in the condition of interest (HBV vs healthy), are cell-type specific, are present in most samples of the condition of interest, and for which predicted target genes are enriched in the receiver cell type. For the top 100 receptor-ligand interactions, cluster grouping was performed for greater statistical certainty and power. Groups included canonical NK cells (CD56dim and CD56bright NK cells), CXCR6+NK cells, monocytes, B cells, CD8+T cells, CD4+T cells, and Kuppfer cells.

Multiplex immunofluorescence

Multiplex immunofluorescence staining was performed on 4 μm FFPE liver biopsies from NUC-HBV patients and healthy controls in the ISO-certified pathology laboratory of Erasmus MC, ensuring diagnostic-grade reproducibility and quality control. Sections were processed on the automated Ventana Benchmark Discovery ULTRA system (Ventana Medical Systems Inc.). Following deparaffinization, antigen retrieval was performed with CC1 solution (Ventana, #950-224) for 32 minutes. To enable multiplex staining without cross-reactivity, sequential primary antibody incubations were interleaved with denaturation steps using CC2 solution (Ventana, #950-123) at 100 °C for 20 minutes.

The following antibodies were included in the multiplex panel: CXCR6 (rabbit polyclonal, Invitrogen, 1:100 dilution, 60 min at 37 °C), IFN-γ (rabbit polyclonal, Abcam, 1:100 dilution, 60 min at 37 °C), CD3 (rabbit monoclonal, clone 2GV6, Ventana, ready-to-use, 60 min at 37 °C), and CD56 (rabbit monoclonal, clone MRQ-42, Ventana, ready-to-use, 60 min at 37 °C). Detection was achieved with Omnimap anti-rabbit HRP (Ventana, #760-4311) in combination with appropriate chromogens: DCC (#760-244, Ventana) for CXCR6, Red610 (#760-245, Ventana) for IFN-γ, Cy5 (#760-238, Ventana) for CD3, and FAM (#760-243, Ventana) for CD56. All slides were counterstained with DAPI (Vector Laboratories) and mounted in Vectashield medium to preserve fluorescence.

Whole-slide imaging was performed using a Zeiss Axioscan 7 at high resolution, capturing both portal tracts and parenchymal regions in their entirety to avoid sampling bias. For quantitative analysis, images were processed using Visiopharm VIS (v2024.07.1.16912x64). Nuclear segmentation was carried out with a U-net deep learning model trained on hepatocyte and lymphocyte nuclei. Segmented nuclei were expanded radially to reconstruct cytoplasmic compartments, ensuring accurate classification of both membrane-associated and intracellular markers. This approach allowed unbiased quantification of up to 88,000 cells per slide, greatly exceeding the depth achievable by manual counting and reducing inter-operator variability.

To validate specificity, antibody performance was confirmed using diagnostic pathology–grade reference tissues (tonsil, placenta, lung), which were included on each staining run as internal positive and negative controls. In addition, hepatocytes within each liver biopsy served as an internal negative control for lymphocyte markers (CD3, CD56, CXCR6, IFN-γ), providing baseline autofluorescence thresholds. Positivity cut-offs were defined accordingly, ensuring that signals reflected true biological expression rather than background fluorescence.

In this analysis we focused specifically on T cells and NK cells, as they represent the principal lymphocyte sources of IFN-γ in the human liver. Within this compartment, the combination of CD56 and CXCR6 expression is uniquely restricted to NK cells and is not observed in other lymphocyte subsets. This biological constraint allowed us to confidently attribute IFN-γ signal to bona fide liver-resident NK cells, while signals from CD3+ cells were interpreted as T cell–

derived. By structuring the analysis in this way, we avoided misclassification of rare or noncanonical cell types and ensured that cytokine production was correctly assigned to the two dominant IFN-γ–producing lymphocyte lineages. The integration of automated high-quality staining, whole-slide scanning, and AI-based segmentation then enabled reproducible and unbiased quantification of IFN-γ production in situ, allowing direct comparison of NK- and T cell contributions between NUC-HBV and healthy liver tissue.

Statistical analyses

Analyses were performed with GraphPad Prism v8 and Python v3.11.3. Comparisons between groups used unpaired two-tailed Student's t-tests or nonparametric equivalents as appropriate. For multiple testing, Benjamini–Hochberg correction was applied. $P < 0.05$ was considered significant.

Reference:

1) https://pubmed.ncbi.nlm.nih.gov/34296197/
2) https://pubmed.ncbi.nlm.nih.gov/34782790/
3) https://pubmed.ncbi.nlm.nih.gov/31597160/
4) https://www.nature.com/articles/ncomms14049
5) https://www.nature.com/articles/s41576-023-00586-w
6) https://academic.oup.com/gigascience/article/9/12/giaa151/6049831?login=false
7) https://genomebiology.biomedcentral.com/articles/10.1186/s13059-017-1382-0
8) https://f1000research.com/articles/10-979/v2
9) https://f1000research.com/articles/5-2122/v2
10) https://www.nature.com/articles/s41587-021-01206-w
11) https://www.biorxiv.org/content/10.1101/2023.06.13.544751v1

Table with antibodies for IF

Antibody	Dilution	Species	Company	Clone	Ab incubation time at 37˚C
**CXCR6**	1:100	rabbit	Invitrogen	Polyclonal	60 minutes
**IFN-y**	1:100	rabbit	Abcam	Polyclonal	60 minutes
**CD3**	ready to use	rabbit	Ventana	2GV6	60 minutes
**CD56**	ready to use	rabbit	Ventana	MRQ-42	60 minutes

Supplementary figures

Fig. S1

A

Immunofluorescent staing with annotation of CD56+ cells in liver in healthy liver (HC IF 6)

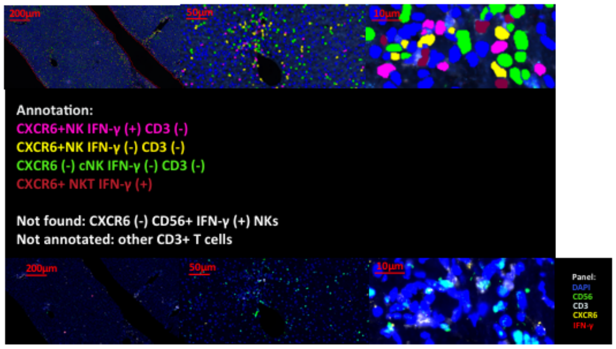

B

Absolute counts of CD56+ NK cells identified by immunofluorescent staining in liver tissue from healthy controls and NUC-treated HBV patients.

Group	cm2	CD56+ CD3- CXCR6- IFNy- cells	CD56+ CXCR6+ IFNy+/- CD3-	CD56+CXCR6+IFNy+ CD3-	CD3- CD56- CXCR6- IFNy-	Total cells	CXCR6NK/cm2
		CD56 cells	CXCR6+NK cells	IFNy+ CXCR6+NK cells	Negative cells	Total cells	
NUC-HBV 1	2.38	6297	458	1	32290	38957	192.22
NUC-HBV 2	1.71	555	765	6	27633	28929	448.14
NUC-HBV 4	0.81	311	1233	2	13217	14741	1518.47
NUC-HBV 5	1.76	269	234	0	27129	27626	133.22
NUC-HBV 6	0.96	181	999	0	13950	15102	1041.85
NUC-HBV 7	3.01	497	652	0	45726	46837	216.30
NUC-HBV 8	2.31	4302	689	14	34839	39773	297.06
NUC-HBV 9	0.65	319	258	18	11227	11795	395.45
NUC-HBV 10	2.07	2565	1114	7	26639	30276	537.14
HC IF 5	3.07	829	3641	2447	42063	46568	1184.23
HC IF 9	2.74	2981	2529	611	34949	40396	921.34
HC IF 6	6.26	9772	13374	5026	65538	88467	2134.79
HC IF 4	2.94	6215	7641	1269	34277	47926	2603.18
HC IF 3	3.67	12153	5514	781	42689	60131	1502.71
HC IF 7	4.03	1714	3982	3361	59795	65500	987.74

Supplementary Figure 1A & B. (A) Machine learning-based cell identification and color-coded annotation of IFN-γ+ immune cells in a healthy liver biopsy from the same donor shown in Figure 1. CD56+CXCR6+CD3− NK cells are consistently identified as the dominant IFN-γ–producing population. No IFN-γ+ CD56+ NK cells lacking CXCR6 expression (likely CD56dim and CD56bright subsets) were detected, (B) Table of CD56+ NK cell counts per sample, including absolute numbers in healthy and NUC-HBV liver samples
Abbreviations: HC IF; healthy control biospy for immunofluorescence, NUC-HBV; NUC-treated chronic HBV patient

Figure panel with individual markers in healthy liver tissue:
Multiplex immunofluorescence of DAPI, CD3, CD56, CXCR6, and IFN-γ, with merged image (lower panel) revealing CD56+ CXCR6+ IFN-γ+ CD3- NK cells in situ

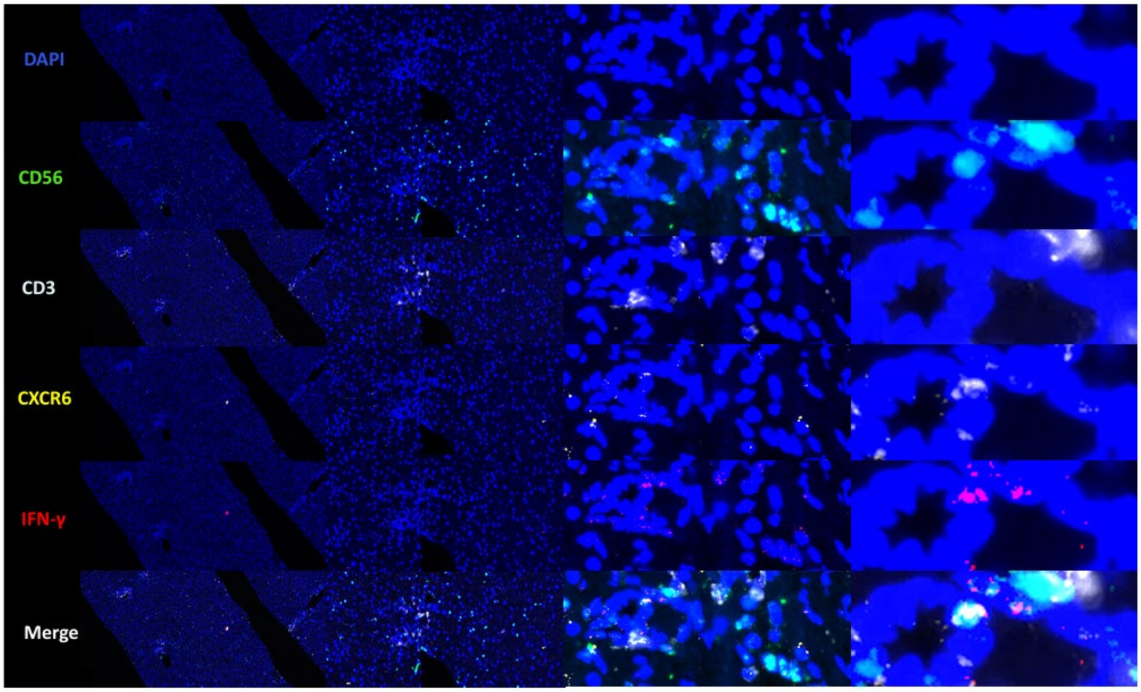

Supplementary figure 1C. Tissue section from the same healthy liver (Healthy 6 (HC IF 6)) donor shown in Figure 1. Each row represents a single stain: DAPI (nuclear), CD56 (NK cell marker), CD3 (T cell marker), CXCR6 (tissue residency), IFN-γ, and a final row showing the merged image. At higher magnification, IFN-γ signal is restricted to CD56+CXCR6+ cells, while neighboring CD56+CXCR6 (-) and CD56+ CXCR6 (+) NK cells remain negative, confirming high specificity of cytokine staining. This panel highlights the spatial colocalization of IFN-γ protein exclusively within CXCR6[illegible] NK cells in healthy liver.

Fig. S2

Figure panel with individual markers in NUC HBV liver tissue:
Multiplex immunofluorescence of DAPI, CD3, CD56, CXCR6, and IFN-γ, with merged image (lower panel) revealing primarily CD3+ T cells in liver

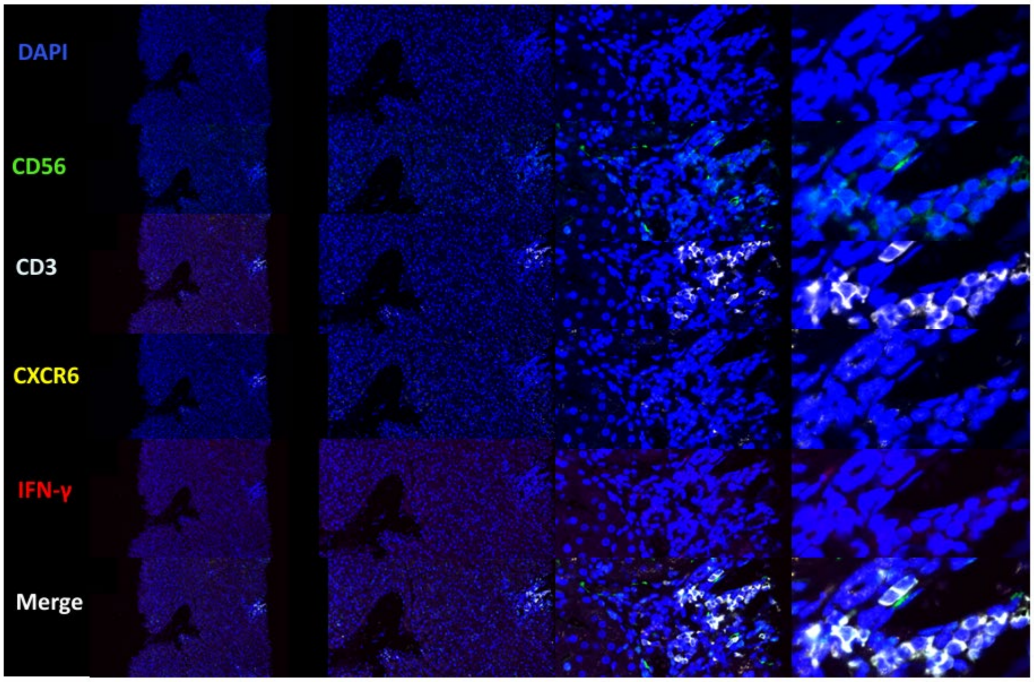

Supplementary figure 2. Tissue section from the same NUC-HBV liver sample shown in Figure 2. Each row displays a single-channel stain: DAPI (nuclear), CD56 (NK cell marker), CD3 (T cell marker), CXCR6 (tissue residency marker), IFN-γ, and a final row showing the merged image. At higher magnification, IFN-γ signal is nearly absent, and no CD56+CXCR6+ cells express IFN-γ. In contrast, a prominent accumulation of CD3+T cells is observed, particularly in periportal regions. This panel illustrates the striking loss of IFN-γ-producing CXCR6+NK cells in NUC-HBV liver, despite increased T cell presence.

Fig. S3

A

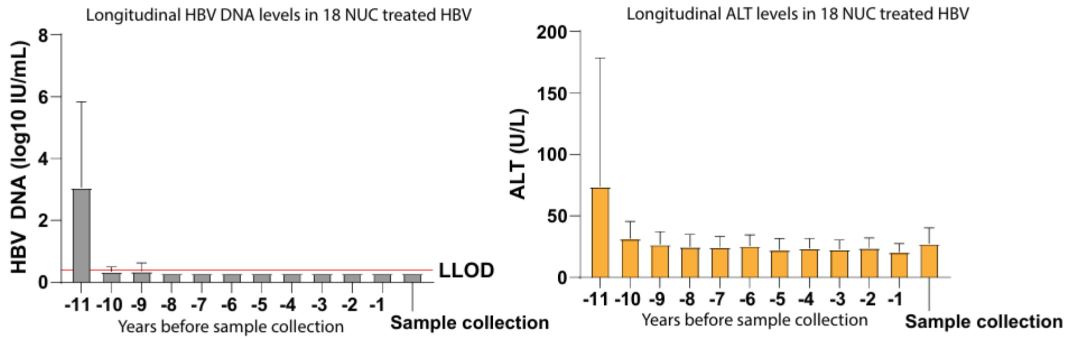

B

Sample ID	HBeAg (IU/ml)	Fibrosis score	Age	Sex	Type of antiviral	ALT (U/L)	HBsAg levels (IU/mL)	Genotype
**HBV1**	negative	F0-F1	36	male	Tenofovir	54	5385	ND
**HBV3**	negative	F0-F1	58	male	Tenofovir	26	74	C
**HBV4**	negative	F0-F1	43	male	Entecavir	63	12447	C
**HBV5**	negative	F0-F1	61	male	Tenofovir	20	4600	ND
**HBV6**	negative	F0-F1	52	male	Tenofovir	18	39	A
**HBV7**	negative	F0-F1	50	male	Tenofovir	45	100	ND
**HBV8**	negative	F0-F1	31	male	Tenofovir	21	1400	ND
**HBV9**	negative	F0-F1	67	male	Entecavir	24	70	B
**HBV10**	negative	F0-F1	59	male	Entecavir	20	1233	F
**HBV12**	negative	F0-F1	69	male	Tenofovir	9	4600	ND
**HBV13**	negative	F0-F1	62	male	Entecavir	36	68	B
**HBV14**	negative	F0-F1	47	male	Tenofovir	15	7195	A
**HBV15**	negative	F0-F1	58	male	Entecavir	34	2	C
**HBV16**	negative	F0-F1	48	male	Entecavir	28	79	B
**HBV17**	negative	F0-F1	59	male	Tenofovir	54	7573	A
**HBV18**	negative	F0-F1	48	male	Entecavir	21	920	ND
**HBV19**	negative	F0-F1	43	female	Tenofovir	24	1	C
**HBV20**	negative	F0-F1	44	male	Entecavir	23	99	C

Supplementary figure 3
(A)Trends in HBV DNA and ALT levels for 18 HBeAg-negative patients showing significant declines after entecavir or tenofovir treatment. (B)The lower panel shows clinical data for each patient.

Fig. S4

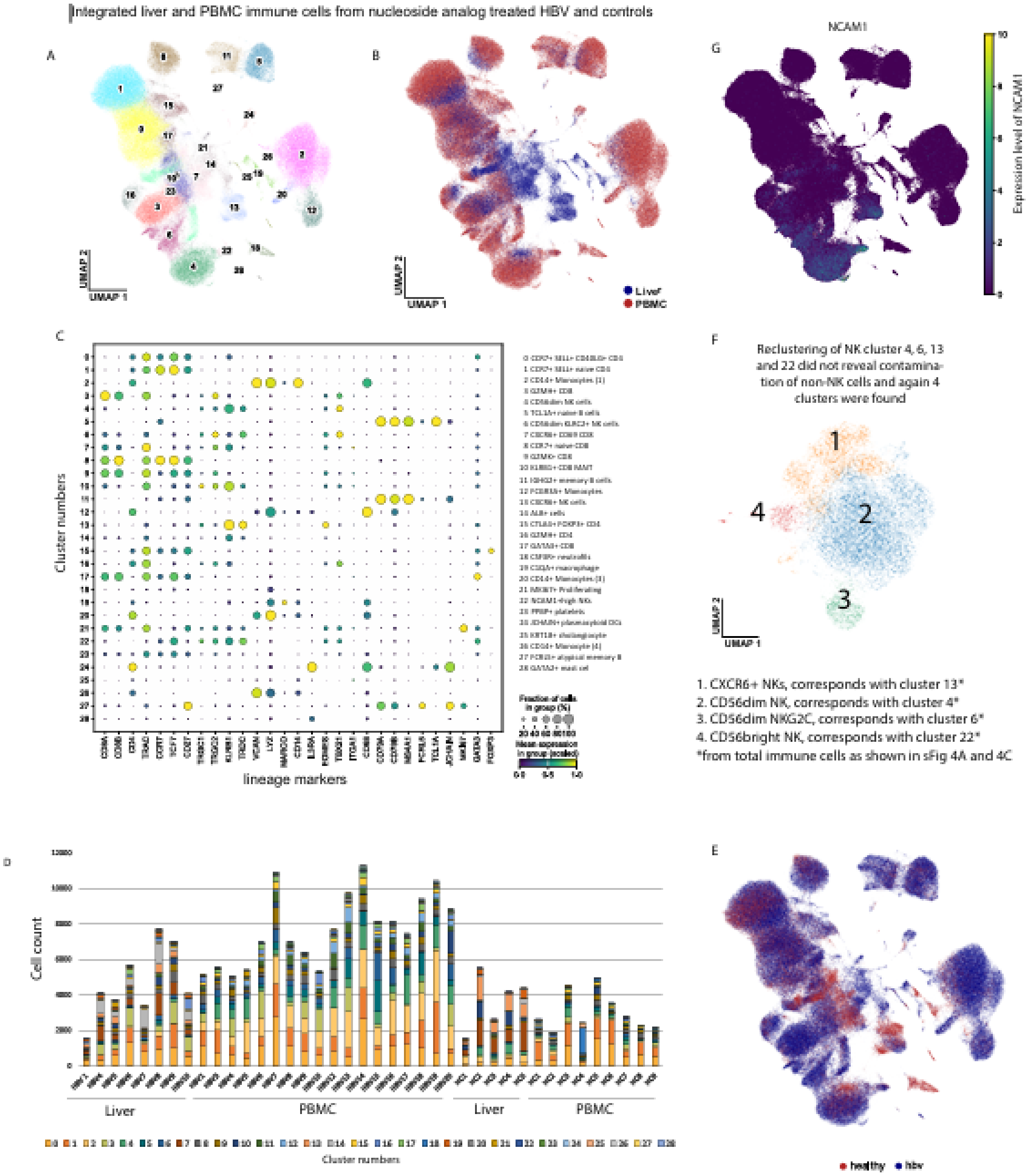

Supplementary Figure 4 A-G

Integration and Clustering of Stably Suppressed HBV Patients with Healthy Control Datasets

The effect of stably suppressed, NUC-treated HBV infection on liver NK cells remains unclear. To address this knowledge gap, we performed single-cell RNA sequencing (scRNAseq) to analyze the transcriptional profiles of liver immune cell populations in an integrated dataset. This dataset included ex vivo liver immune cells, ex vivo PBMCs, and liver and PBMC samples from healthy controls.

Our study compared paired PBMC and liver immune cells from HBV-infected individuals (N=9) and an additional 9 PBMC samples from NUC-treated HBV patients with those from healthy control liver (N=5) and PBMC samples, allowing us to elucidate HBV-specific liver changes. The dataset comprised samples from NUC-treated HBV subjects with stable suppression (median ALT: 26) (Supplementary Table 1), and high-quality healthy controls from Ramachandran et al. 2019 (GSE136103), Steele et al. 2020 (GSE155698), and Sinha et al. 2022 (GSE157789), all published by Nature.

Supplementary Figure 1 illustrates the successful integration of the PBMCs and liver immune cells from NUC-treated HBV and healthy control datasets and clustering of the immune populations from blood and liver (A and B). In the scRNA-seq data unsupervised clustering (Louvain set at 1.0) identified 28 distinct cell clusters representing both circulating and liver immune cells (C). These clusters included natural killer (NK) cells (NKG7, GNLY, SPON2, NCAM1 (G)), T cells (CD3D, TRAC), B cells (MS4A1, CD79A), plasma cells (IGHG1, IGHA1, JCHAIN), monocytes (CD14, FCN1, MNDA), and proliferating cells (MKI67, BIRC5). Importantly, our analysis showed that the integrated PBMC data captured the full immunological diversity of both circulating and liver immune populations in all patients (D), demonstrating the robustness of our approach. We focused on clusters 4, 6, 13, and 22 for further analysis, specifically targeting NK cells. Clusters 4, 6, and 22 were found in both liver and blood (B) and were classified as conventional NK cells, while cluster 13 was only found in the liver and represented liver-resident CXCR6+ NK cells (A-C). These populations were selected for further NK cell analysis and underwent reclustering to obtain more detailed data on subtypes. However, reclustering did not reveal any unique NK cell phenotypes other than CD56dim, CD56bright, CD56dim NKG2C, or liver CXCR6 NK cells (F).

Batch correction figure

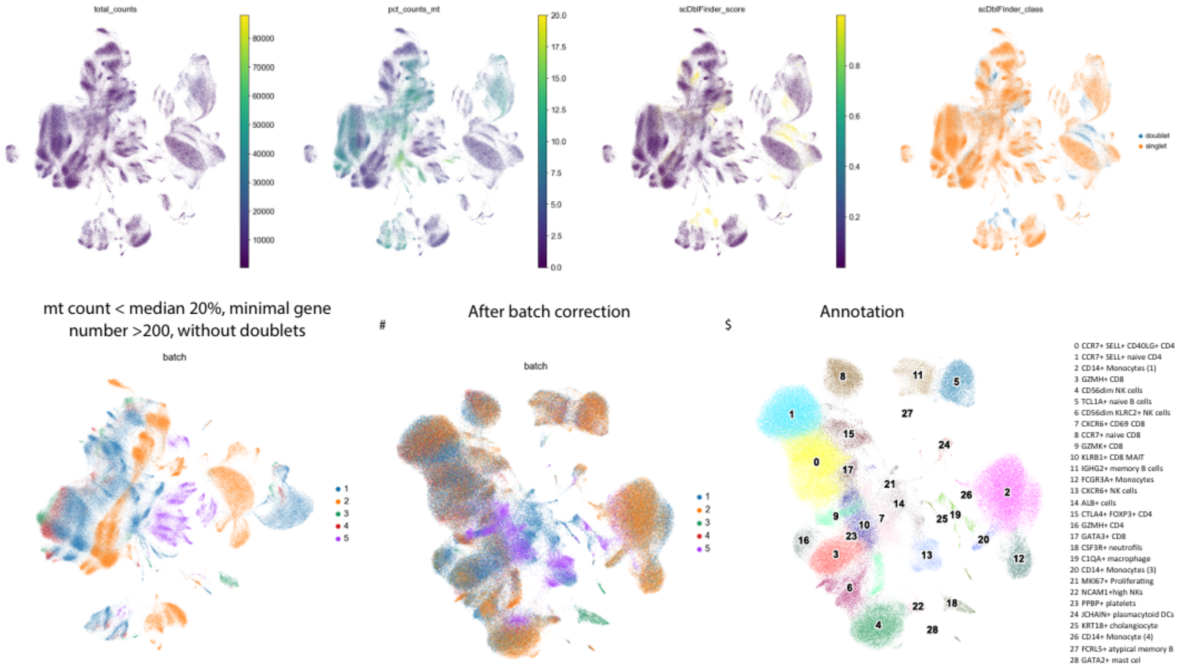

Batch number

1. HBV PBMC + Liver	(3' chemistry v3)	
2. HBV PBMC	(5' chemistry v2)	
3. Healthy PBMC	(3' chemistry v3)	(GSE155698)
4. Healthy PBMC	(3' chemistry v2)	(GSE157789)
5. Healthy liver	(3' chemistry v2)	(GSE136103)

Supplementary figure 4H. Batch correction and integration of scRNA-seq data from five distinct batches. Top panel: Overview of total counts (left), percentage of mitochondrial counts (middle), and scDbiFinder score and doublets (right) for all datasets. Bottom panel: (*) Before batch correction, showing distinct batch effects across the five datasets. (#) After successful integration of clusters, no chemistry-related batch effects are observed. Only cluster 8 (neutrophils) are specific to a PBMC dataset (3). Neutrophil capture are harder to detect with 10 × 3'v2 data because of their low RNA and high nuclease content. ($) Full annotation of clusters post-integration

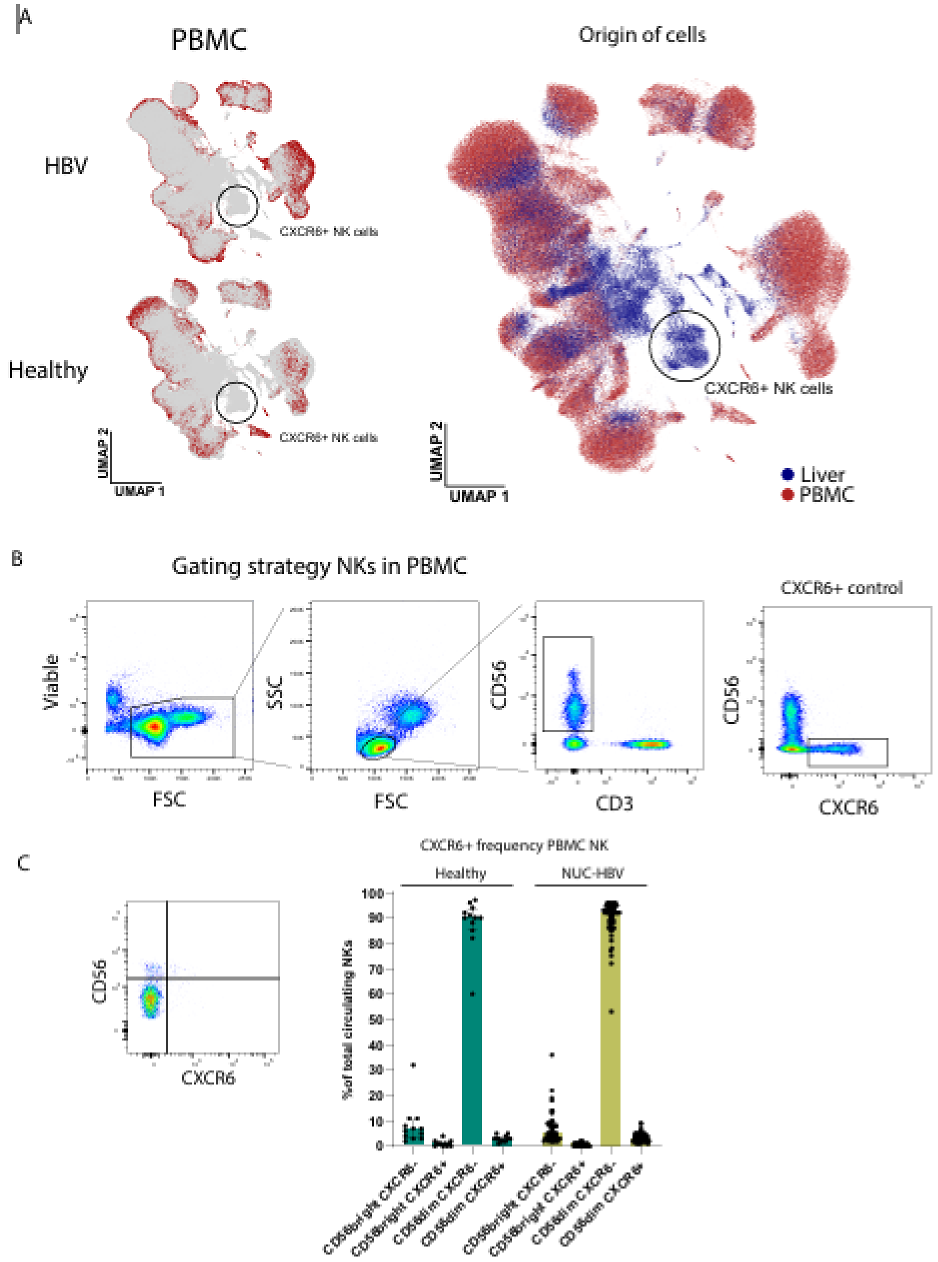

Supplementary Figure 5.
(A)Feature plot showing single-cell distribution in PBMCs from healthy and NUC-HBV patients, where red dots represent cells from the respective group, and grey dots indicate other cells. Right panel shows origin of cells, blue is liver and red is PBMC.
(B) Flow cytometry gating strategy for identifying circulating NK cells in 54 fresh frozen samples. The gating process includes the selection of viable lymphocytes, followed by the identification of CD56right and CD56dim CD3- NK cells. A sample with CXCR6high CD3+ cells is included as a positive control for the CXCR6 gate.
(C) CXCR6 expression is very low on circulating NK cells in both healthy individuals and HBV patients undergoing long-term NUC treatment. CXCR6+ CD56bright NK cells are rarely observed in blood, which is consistent with scRNAseq data indicating a distinct liver-specific cluster of CXCR6+ cells that is not present in PBMCs. Additionally, while low CXCR6 gene expression is observed in CD56dim NK cells in blood (flowcytometry and scRNAseq), these cells are distinct from the CXCR6+ EOMES+ IFNG+ liver NK cells (data not shown).

Fig. S5

Fig. S6

Post hoc analysis: 24-week NUC initiation in active HBV

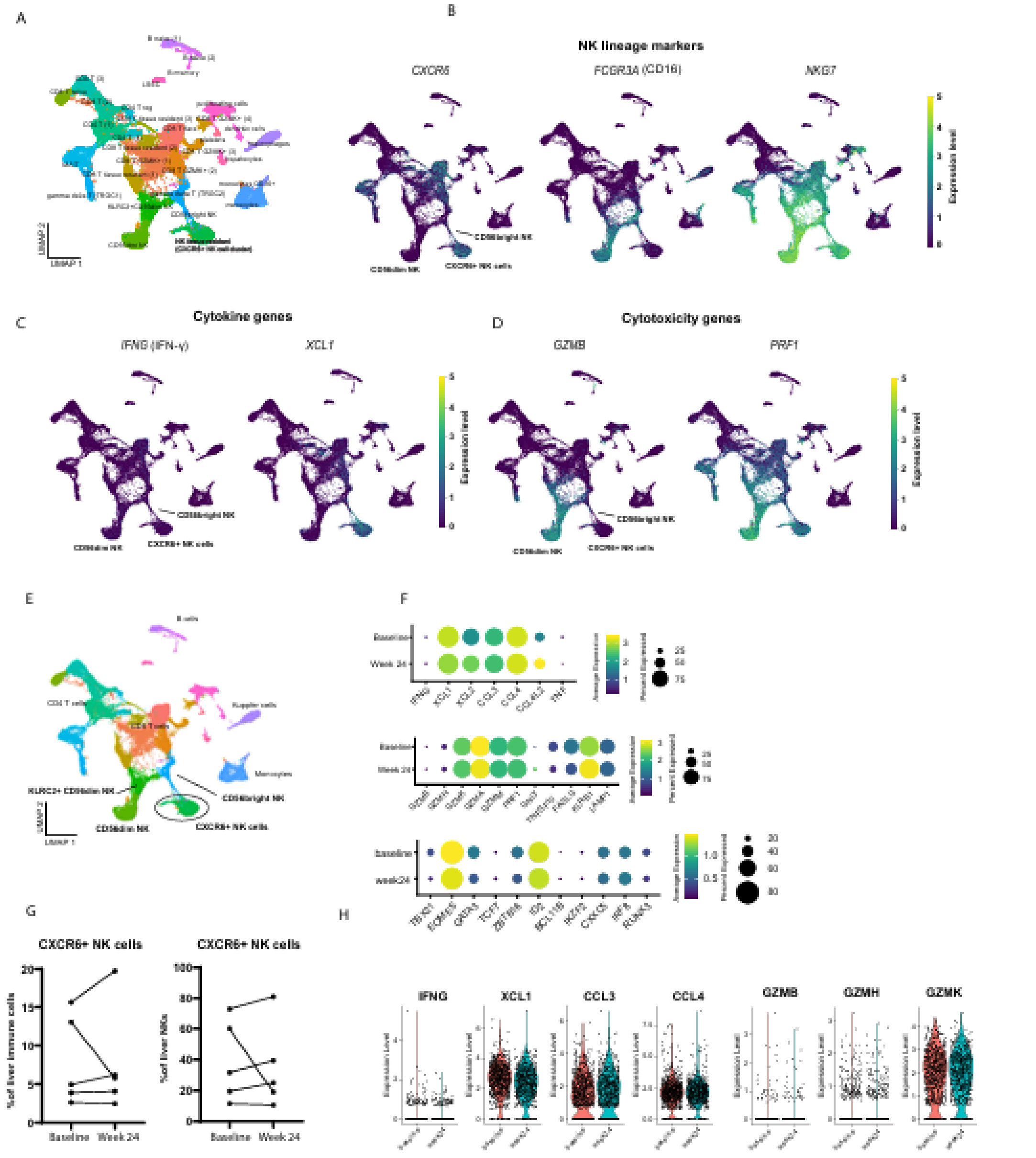

Supplementary figure 6
CXCR6+ NK cell annotation and cytokine profile in an independent scRNA-seq dataset
UMAP projections of liver immune cells from five chronic HBV patients sampled before and after NUC therapy (Nkongolo et al., J Clin Invest 2023;133(1):e158903) are shown, with annotation of major intrahepatic immune cell populations and NK cell subsets (A, E). CXCR6+ NK cells formed a distinct cluster alongside CD56dim NK cells, KLRC2+ CD56dim NK cells, and CD56bright NK cells—matching the subset distribution in our dataset.

Feature plots demonstrate comparable expression of NK lineage markers (B) and selected cytokine genes (C), with CXCR6+ NK cells showing low IFNG expression but preserved XCL1 expression (C, F, H), consistent with our findings. Cytotoxicity genes (PRF1, GZMB) were also low in CXCR6+ NK cells (D, F).

Panel F summarizes cytokine-, cytotoxicity-, and transcription factor–associated gene expression in dot plot format. Panel G shows stable CXCR6+ NK cluster frequencies over 24 weeks of NUC therapy. Panel H displays violin plots of selected gene expression, highlighting the absence of significant changes between baseline and week 24.

Fig. S7

A

Data origin: Liver (supplementary table 2 [scRNAseq of Liver FNA and control])

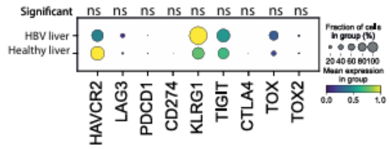

B

Data origin: Liver (Nkongolo et al., J Clin Invest 2023;133(1):e158903)

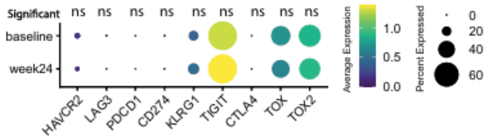

Supplementary Figure 7. Immune checkpoint markers in Liver and circulating NK cells
(A) Integrated PBMC scRNA-seq dataset (Supplementary Table 2) demonstrates comparable expression of immune checkpoint markers between NUC-HBV and controls in CXCR6+NK cells (clsuter 13). Color scale: Yellow indicates higher gene expression; blue indicates lower gene expression.
(B) Longitudinal PBMC scRNA-seq analysis during 24 weeks of NUC therapy (Nkongolo et al., J Clin Invest 2023) shows stable expression of immune checkpoint genes with no recovery or further decline over time.

Supplementary tables

Table S1

Group	ALT (U/L)	Ethnicity	Gender	Age	Metavir	Therapy
NUC-HBV 1	22	Caucasian	Male	39	F0-F1	Lamivudine
NUC-HBV 2	34	Asian	Male	40	F0-F1	Lamivudine
NUC-HBV 3	45	Asian	Male	50	F0-F1	Tenofovir
NUC-HBV 4	46	Caucasian	Male	38	F0-F1	Lamivudine
NUC-HBV 5	33	Caucasian	Male	46	F0-F1	Lamivudine
NUC-HBV 6	36	Asian	Male	28	F0-F1	Lamivudine
NUC-HBV 7	75	Caucasian	Male	35	F0-F1	Entecavir
NUC-HBV 8	29	Caucasian	Male	37	F0-F1	Adefovir
NUC-HBV 9	23	Asian	Female	45	F0-F1	Lamivudine
NUC-HBV 10	18	Caucasian	Male	48	F0-F1	Lamivudine
HC IF 1	NA	Asian	Male	38	F0	
HC IF 2	NA	Caucasian	Male	59	F0	
HC IF 3	21	Caucasian	Male	51	F0	
HC IF 4	61	Caucasian	Male	27	F0	
HC IF 5	24	Caucasian	Female	52	F0	
HC IF 6	28	Caucasian	Female	55	F0	
HC IF 7	NA	Caucasian	Female	63	F0	
HC IF 8	NA	Asian	Female	28	F0	
HC IF 9	24	Caucasian	Female	57	F0	

Supplementary table 1. Cohort of altruistic healthy liver donors (screening biopsies) and NUC-HBV patient biospies, collected as part of end of treatment biopsy or as screening for NUC-cessation. NA; not available, IF; immunofluorescence, HC; healthy control

Table S2

Sample ID	Type of patient	Database	Type of samples	Location of study	Sex	Type of antiviral	Age	ALT (U/L)	HBsAg levels (IU/mL)	Genotype
HBV1	NUC HBV	tbd	blood/liver	Rotterdam, Netherlands	male	TDF	36	54	5385	ND
HBV3	NUC HBV	tbd	blood	Rotterdam, Netherlands	male	TDF	58	26	74	C
HBV4	NUC HBV	tbd	blood/liver	Rotterdam, Netherlands	male	ETV	43	63	12447	C
HBV5	NUC HBV	tbd	blood/liver	Rotterdam, Netherlands	male	TDF	61	20	4600	ND
HBV6	NUC HBV	tbd	blood/liver	Rotterdam, Netherlands	male	TDF	52	18	39	A
HBV7	NUC HBV	tbd	blood/liver	Rotterdam, Netherlands	male	TDF	50	45	100	ND
HBV8	NUC HBV	tbd	blood/liver	Rotterdam, Netherlands	male	TDF	31	21	1400	ND
HBV9	NUC HBV	tbd	blood/liver	Rotterdam, Netherlands	male	ETV	67	24	70	B
HBV10	NUC HBV	tbd	blood/liver	Rotterdam, Netherlands	male	ETV	59	20	1233	F
HBV12	NUC HBV	tbd	blood	Rotterdam, Netherlands	male	TDF	69	9	4600	ND
HBV13	NUC HBV	tbd	blood	Rotterdam, Netherlands	male	ETV	62	36	68	B
HBV14	NUC HBV	tbd	blood	Rotterdam, Netherlands	male	TDF	47	15	7195	A
HBV15	NUC HBV	tbd	blood	Rotterdam, Netherlands	male	ETV	58	34	2	C
HBV16	NUC HBV	tbd	blood	Rotterdam, Netherlands	male	ETV	48	28	79	B
HBV17	NUC HBV	tbd	blood	Rotterdam, Netherlands	male	TDF	59	54	7573	A
HBV18	NUC HBV	tbd	blood	Rotterdam, Netherlands	male	ETV	48	21	920	ND
HBV19	NUC HBV	tbd	blood	Rotterdam, Netherlands	female	TDF	43	24	1	C
HBV20	NUC HBV	tbd	blood	Rotterdam, Netherlands	male	ETV	44	23	99	C
HC1	Healthy	GSE157789	blood	Calgary, Alberta, Canada	female		37			
HC2	Healthy	GSE157789	blood	Calgary, Alberta, Canada	male		64			
HC3	Healthy	GSE157789	blood	Calgary, Alberta, Canada	male		44			
HC4	Healthy	GSE157789	blood	Calgary, Alberta, Canada	male		44			
HC5	Healthy	GSE157789	blood	Calgary, Alberta, Canada	female		46			
HC1	Healthy	GSE136103	liver	Edinburgh, UK	male		59			
HC2	Healthy	GSE136103	liver	Edinburgh, UK	male		52			
HC3	Healthy	GSE136103	liver	Edinburgh, UK	male		70			
HC4	Healthy	GSE136103	liver	Edinburgh, UK	female		56			
HC5	Healthy	GSE136103	liver	Edinburgh, UK	male		50			
HC6	Healthy	GSE155698	blood	Michigan, USA	female		70			
HC7	Healthy	GSE155698	blood	Michigan, USA	male		71			
HC8	Healthy	GSE155698	blood	Michigan, USA	male		63			
HC9	Healthy	GSE155698	blood	Michigan, USA	male		60			

Supplementary Table 2: Clinical data of lon-term nucleos(t)ide analog-treated HBeAg-negative HBV subjects with scRNA-seq data from PBMCs and fine needle aspirate liver biopsies, including healthy subjects and their respective GSE accession numbers. Abbreviations: ALT (U/L) – Alanine Aminotransferase; HBsAg – Hepatitis B Surface Antigen; HBeAg – Hepatitis B e Antigen; TDF – Tenofovir; ETV – Entecavir

Table S3

		NUC-treatment	Healthy control	P-value
**N=**		**44**	**10**	
**Age (years)**	Median (IQR)	**47 (41-50)**	**49 (43-62)**	**0.2**
**Ethnicity**				**0.216**
Caucasian		**8 (19%)**	**5 (50%)**	
Asian		**13 (30%)**	**2 (20%)**	
Black		**7 (16%)**	**1 (10%)**	
Other		**16 (36%)**	**2 (20%)**	
**Sex**	M/F	**31/13**	**8/2**	**0.17**
**NA regime (ETV/TDF)**		**25/19**	**-**	**-**
**NA treatment duration (weeks)**	Mean (SD)	**384 (227)**	**-**	**-**
**HBeAg-negative**		**44 (100%)**	**10 (100%)**	**-**
**HBsAg baseline (IU/mL)**	Median (IQR)	**1450 (307-6050)**	**-**	
**HBV DNA (IU/mL)**	Median (IQR)	**Undetectable**	**-**	**-**
**ALT baseline (U/L)**	Median (IQR)	**23 (16-29)**	**30 (18-33)**	**0.44**
**Fibrosis F0-F1**		**44 (100%)**	**-**	**-**

Supplementary Table 3: Characteristics of the flow cytometry study cohort from Erasmus MC, Rotterdam, the Netherlands. Abbreviations: ALT (U/L) – Alanine Aminotransferase; HBsAg – Hepatitis B Surface Antigen; HBeAg – Hepatitis B e Antigen; TDF – Tenofovir; ETV – Entecavir

Table S4

		1	2	3	4	5	6	7	8
1	r=	—							
	p-value	—							
2	r=	-0.48	—						
	p-value	0.24	—						
3	r=	-0.44	0.60	—					
	p-value	0.27	0.12	—					
4	r=	0.57	-0.31	-0.29	—				
	p-value	0.15	0.46	0.49	—				
5	r=	0.31	0.38	0.40	0.45	—			
	p-value	0.46	0.36	0.33	0.27	—			
6	r=	-0.29	-0.14	0.24	-0.02	0.10	—		
	p-value	0.50	0.75	0.57	0.98	0.84	—		
7	r=	-0.21	**0.76**	0.10	-0.19	0.43	-0.07	—	
	p-value	0.62	**0.04**	0.82	0.66	0.30	0.88	—	
8	r=	0.05	-0.45	-0.42	0.02	0.07	0.38	0.07	—
	p-value	0.93	0.27	0.30	0.98	0.88	0.36	0.88	—

1. **Age**
2. **HBsAg** (IU/mL)
3. **ALT** (U/L)
4. **Treatment duration** (years)
5. **CD56dim NK cells (cluster 4)**
6. **CD56dim NKG2C^{+} NK cells (cluster 6)**
7. **CXCR6+ NK cells (cluster 13)**
8. **CD56bright NK cells (cluster 22)**

Supplementary table 4.
Correlation matrix of clinical parameters (age (years), serum HBsAg (IU/mL), ALT (U/L), and NUC treatment duration in months) with liver NK cell subset frequencies (CXCR6+ NK cells [cluster 13], CD56dim NK cells [cluster 4], CD56dim KLRC2+ NK cells, and CD56bright NK cells) in long-term NUC-treated HBV patients (cohort details supplementary table 2).
