# Supplementary material for: Persistent loss of intrahepatic IFN-γ in HBV is linked to selective impairment of liver-resident CXCR6+NK cells despite long-term NUC therapy
Source: JHEP Rep. 2026 Apr 18;8(7):101865. doi: 10.1016/j.jhepr.2026.101865 (PMC13264066; doi:10.1016/j.jhepr.2026.101865)
Supplement: Multimedia component 2 [file mmc2.pdf]

JHEP Reports

CTAT methods

Tables for a "Complete, Transparent, Accurate and Timely account" (CTAT) are now mandatory for all revised submissions. The aim is to enhance the reproducibility of methods.

- Only include the parts relevant to your study
- Refer to the CTAT in the main text as 'Supplementary CTAT Table'
- Do not add subheadings
- Add as many rows as needed to include all information
- Only include one item per row

If the CTAT form is not relevant to your study, please outline the reasons why:

1.1 Antibodies

Name	Citation	Supplier	Cat no.	Clone no.
PD1 PerCP-eFluor710	Invitrogen, Thermo Fisher Scientific	eBioscience	61-9981-42	eBioJ105
TIM-3-PerCP	R&D Systems	R&D	FAB2365C	344823
CD56-APC vio770	Miltenyi Biotec	Miltenyi	130-114-548	REA196
TRAIL-Alexa Fluor488	R&D Systems	R&D	FAB687G	75402
CXCR6-APC	BioLegend	Biolegend	367711	K041E5
TIGIT-BV786	BD Biosciences	BD Bioscience	741182	741182
KLRG1-PE	BioLegend	Biolegend	367710	SA231A2
CD3-PEeFluor610	Thermo Fisher Scientific	eBioscience	61-0038-42	UCHT1
HLA-DR-PE-Cy5	Thermo Fisher Scientific	eBioscience	15-9956-42	LN3
CD38-PeCy7	Invitrogen, Thermo Fisher Scientific	eBioscience	25-038-842	HB7
viability-BV570	Miltenyi Biotec	Miltenyi	130-114-548	-
IFN-γ-PE-Cy7	Thermo Fisher Scientific	eBioscience	61-7319-42	4S.B3
CD107a-BV605	BioLegend	Biolegend	328633	LAMP1
anti-rabbit HRP	Roche Diagnostics	Ventana	760-4311	
anti-IFNγ	Abcam	Abcam	ab9657	Polyclonal
anti-CD3	Roche Diagnostics	Ventana	790-4341	2GV6
anti-CD56	Roche Diagnostics	Ventana	790-4341	MRQ-42
anti- CXCR6	Invitrogen, Thermo Fisher Scientific	Invitrogen	61-9981-42	Polyclonal

1.2 Cell lines

Name	Citation	Supplier	Cat no.	Passage no.	Authentication test method
na					

1.3 Organisms

Name	Citation	Supplier	Strain	Sex	Age	Overall n number
na						

1.4 Sequence based reagents

Name	Sequence	Supplier
na		

1.5 Biological samples

Description	Source	Identifier
na		

1.6 Deposited data

Name of repository	Identifier	Link
Data is publically available and has been deposited in GEO	GSE247322.	https://www.ncbi.nlm.nih.gov/geo/) with accession number

1.7 Software

Software name	Manufacturer	Version
FlowJo version 10.1	(Tree Star Inc.).	10.1
VIS 2024.07.1.16912x64	Visiopharm®	2024.07.1
GraphPad Prism	Prism	8
Python	Python	3.11.3

1.8 Other (*e.g.* drugs, proteins, vectors etc.)

IL-12	Milteny	130-096-704
IL-18	MBL	B001-5

1.9 Please provide the details of the corresponding methods author for the manuscript:

Andre Boonstra

Department of Gastroenterology and Hepatology, Erasmus Medical Center, Rotterdam, the Netherlands.

Email: p.a.boonstra@erasmusmc.nl

2.0 Please confirm for randomised controlled trials all versions of the clinical protocol are included in the submission. These will be published online as supplementary information.

na
