# Supplementary material for: Persistent loss of intrahepatic IFN-γ in HBV is linked to selective impairment of liver-resident CXCR6+NK cells despite long-term NUC therapy
Source: JHEP Rep. 2026 Apr 18;8(7):101865. doi: 10.1016/j.jhepr.2026.101865 (PMC13264066; doi:10.1016/j.jhepr.2026.101865)
Supplement: Multimedia component 4 [file mmc4.pdf]

Research article

JHEP|Reports

Persistent loss of intrahepatic IFN-γ in HBV is linked to selective impairment of liver-resident CXCR6+NK cells despite long-term NUC therapy

Authors

Boris J.B. Beudeker, Diren Arda Karaoglu, Shirin Nkongolo, ..., Robert J. de Knegt, Harmen J.G. van de Werken, Andre Boonstra

Correspondence

p.a.boonstra@erasmusmc.nl (A. Boonstra).

Graphical abstract

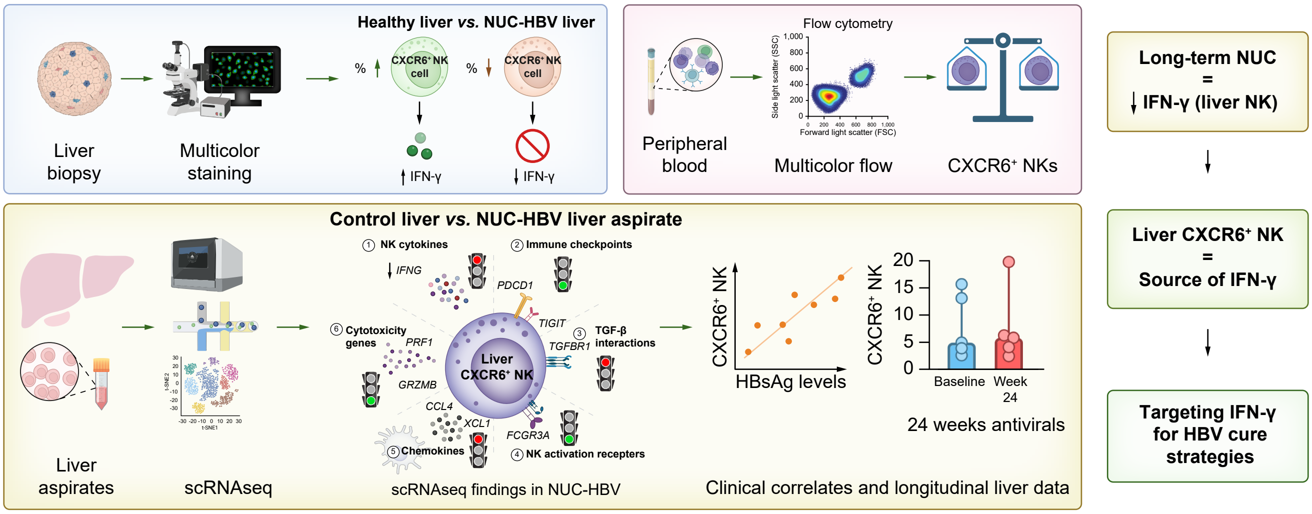

Highlights:

- Multiplex immunofluorescence identifies CXCR6+NK cells as the dominant *in situ* source of IFN-γ in healthy human liver.
- In NUC-treated HBV, intrahepatic CXCR6+NK cells show near-complete loss of IFN-γ protein expression.
- scRNAseq of liver fine-needle aspirates shows transcriptional downregulation of IFNG but not cytotoxicity programs in CXCR6+NK cells.
- The IFN-γ defect is liver-restricted and not observed in blood, consistent with the tissue-restricted nature of CXCR6+NK cells.
- Cell–cell interaction analysis reveals reduced pro-inflammatory signaling and enrichment of TGF-β–associated pathways in NUC-HBV liver.

Impact and implications:

Effective antiviral therapy for chronic HBV suppresses viral replication but does not provide cure, indicating persistent defects in intrahepatic antiviral immunity. By combining protein-level analysis of liver tissue from healthy living donors and NUC-treated HBV liver biopsies with single-cell RNA-seq of fine-needle aspiration–derived immune cells from clinically stable, long-term NUC-treated patients, we directly examined IFN-γ regulation in the human liver—an immune compartment that is largely inaccessible in this patient group. We demonstrate that IFN-γ is produced *in situ* predominantly by CXCR6+NK cells in healthy liver and is selectively reduced in the liver of NUC-treated patients, despite normal alanine aminotransferase levels, absence of fibrosis, and lack of inflammatory or exhaustion signatures, while no corresponding defect is observed in blood. These findings identify a liver-restricted, non–exhaustion-driven impairment of NK-cell function and suggest that future therapeutic strategies should focus on restoring intrahepatic immunity—potentially via modulation of TGF-β–associated pathways or CXCR6+NK cell IFN-γ production.

https://doi.org/10.1016/j.jhepr.2026.101865
© 2026 The Author(s). Published by Elsevier B.V. on behalf of European Association for the Study of the Liver (EASL). This is an open access article under the CC BY license (http://creativecommons.org/licenses/by/4.0/). JHEP Reports, 2026, 8, 1–9

JHEP|Reports

Persistent loss of intrahepatic IFN-γ in HBV is linked to selective impairment of liver-resident CXCR6+NK cells despite long-term NUC therapy

Boris J.B. Beudeker[1,†], **Diren Arda Karaoglu**[1,†], Shirin Nkongolo[2,3], Gertine W. van Oord[1], Zwier M.A. Groothuismink[1], Karishma A. Lila[4], Adam J. Gehring[5], Thierry van den Bosch[4,6], Robert J. de Knegt[1], Harmen J.G. van de Werken[7], Andre Boonstra[1,*]

JHEP Reports **2026**. vol. 8 | 1–9

Check for updates

Background & Aims: Chronic HBV infection remains incurable in most patients despite long-term nucleos(t)ide analog (NUC) therapy. Interferon-γ (IFN-γ) is a cornerstone of antiviral immunity, but its *in situ* source and status in the human liver remain unknown. We aimed to define the primary source of IFN-γ in healthy liver and determine how this axis is altered in stably suppressed NUC-HBV.

Methods: Multiplex immunofluorescence for CD3, CD56, CXCR6, and IFN-γ was performed on liver biopsies from patients with NUC-HBV (n = 9) and healthy donors (n = 7), with whole-slide scanning and AI-based segmentation for unbiased *in situ* cell quantification. Single-cell RNA sequencing (scRNAseq) was conducted on paired blood and liver fine-needle aspirates (FNAs) from NUC-HBV (liver n = 9, blood n = 18) and integrated with control datasets (liver n = 5, blood n = 9). Differential gene expression was used for transcriptional readout, and cell–cell interaction analysis mapped altered signaling networks.

Results: In healthy liver, CXCR6-positive natural killer (CXCR6+NK) cells were the dominant IFN-γ producers (24.2%; IQR 14.1–67.2%). In NUC-HBV, these cells were reduced (675 *vs.* 1,918; $p = 0.012$) and rarely expressed IFN-γ (0.2%; IQR 0.0–1.4%), despite normal alanine aminotransferase and absence of fibrosis. To validate transcriptionally, we performed scRNAseq on FNA and paired blood. A single, liver-restricted CXCR6+NK cluster was identified, and showed selective downregulation of *IFNG* and chemokines (*XCL1*, *CCL3*, *CCL4*), while cytotoxic genes (*GZMB*, *PRF1*) were preserved. Interaction analysis revealed reduced pro-inflammatory signaling and enrichment of transforming growth factor-beta–associated pathways. CXCR6+NK frequency correlated with serum HBsAg ($p = 0.037$) but was unchanged after 24 weeks of NUC in a longitudinal dataset.

Conclusions: Long-term NUC therapy does not restore intrahepatic IFN-γ. Loss and transcriptional reprogramming of CXCR6+NK may contribute to a stable, altered immune state, representing a target for immune-based HBV cure strategies.

© 2026 The Author(s). Published by Elsevier B.V. on behalf of European Association for the Study of the Liver (EASL). This is an open access article under the CC BY license (http://creativecommons.org/licenses/by/4.0/).

Introduction

Chronic HBV infection affects over 250 million people globally and remains a leading cause of cirrhosis and hepatocellular carcinoma (HCC).[1] While nucleos(t)ide analogs (NUCs) achieve durable viral suppression, they rarely result in functional cure because of the persistence of covalently closed circular DNA (cccDNA). As a result, long-term therapy is required, and new curative strategies are urgently needed.[2,3]

Interferon-gamma (IFN-γ) plays a central role in the control of HBV by enhancing antigen presentation, activating antiviral gene expression, and contributing to non-cytolytic suppression of viral replication and cccDNA activity, as shown in both human and experimental models.[4–10] In the liver, IFN-γ can be produced by multiple innate and adaptive immune subsets, including natural killer (NK) cells, which are highly enriched in hepatic tissue.[11]

A longstanding hypothesis in the HBV field suggests that intrahepatic IFN-γ production is diminished in chronic infection, particularly within NK cells.[12–14] However, direct evidence from human liver tissue is limited, and it remains unclear whether IFN-γ expression is restored under long-term NUC-mediated viral suppression.

To address this, we analyzed well-preserved liver tissue from organ donors and NUC-treated patients with HBV using multiplex immunofluorescence on core needle biopsies. This *in situ* approach allowed us to characterize immune cell phenotypes and cytokine expression in intact tissue sections, without the confounding effects of *ex vivo* stimulation. To validate and extend these observations, we performed single-cell RNA sequencing (scRNAseq) on unstimulated liver fine-needle aspirates (FNAs) and matched peripheral blood samples from a second HBV cohort, integrating the data with publicly available control liver datasets.

* Corresponding author. Address: Wytemaweg 80, 3015 CN Rotterdam, Post Office 2040, 3000 CA Rotterdam, the Netherlands.
E-mail address: p.a.boonstra@erasmusmc.nl (A. Boonstra).
† These authors share first authorship.
https://doi.org/10.1016/j.jhepr.2026.101865

We observed consistently low IFN-γ expression in NUC-treated HBV livers, particularly within liver-resident CXCR6-positive natural killer (CXCR6+NK) cells. This intrahepatic finding was not mirrored in the circulation and appeared independent of NUC treatment, suggesting a persistent and liver-associated defect. These insights highlight intrahepatic IFN-γ loss as a stable feature of chronic HBV under NUC therapy and position liver-resident NK cells as a potential therapeutic target to restore antiviral immunity and advance curative strategies.

Patients and methods

A detailed description of the methods is available in the supplementary materials. In brief, patients with chronic HBV from Erasmus MC, Rotterdam, The Netherlands were enrolled; they were adults who were HBeAg-negative with stable viral suppression (HBV DNA <80 IU/ml) and on NUC therapy for >3 years. Exclusion criteria were advanced fibrosis (elastography >7.0 kPa), hepatic decompensation, HCC history, coinfections, autoimmune or metabolic liver diseases, malignancies, or recent pregnancy. Formalin-fixed paraffin-embedded liver biopsies from patients with HBV and healthy controls were obtained through our pathology biobank; these samples were not stimulated to preserve their *in situ* immune state. Sections were stained for CXCR6, IFN-γ, CD3, and CD56 using the Ventana Discovery ULTRA system (Roche Ventana Medical Systems, Tucson, AZ, USA) , and whole-slide imaging was performed on a Zeiss Axioscan 7 (Carl Zeiss AG, Oberkochen, Germany). Quantification was conducted using Visiopharm® software (Visiopharm A/S, Hørsholm, Denmark) with AI-based segmentation.

Peripheral blood mononuclear cells (PBMCs) were isolated from heparinized blood using Ficoll separation. Intrahepatic leukocytes were obtained via FNAs and processed for scRNAseq within 1 h to maintain the unstimulated *in situ* transcriptional profile. scRNAseq was performed using the 10 × Genomics Single Cell 3′ and 5′ kits and sequenced on the NovaSeq6000 platform (Illumina, San Diego, CA, USA). In addition to our own data, we incorporated publicly available human liver and PBMC scRNAseq datasets (GSE136103; GSE155698; and GSE157789).[15–17] Quality control, normalization, batch integration, and clustering were performed using scanpy and scvi tools. PBMCs were additionally stained with antibodies targeting NK cell markers, including CXCR6 and CD56, and analyzed on a BD Symphony A3 cytometer (Becton, Dickinson and Company, Franklin Lakes, NJ, USA).

Ethics

This study was conducted according to the guidelines of the Declaration of Helsinki and the principles of Good Clinical Practice. The ethical review board of the Erasmus MC approved the study, registered as MEC-2008-146 and MEC-2010-039.

Results

In healthy liver, CXCR6+NK cells are the dominant source of IFN-γ protein under steady-state conditions

To investigate whether lymphocytes produce IFN-γ under steady-state conditions in the human liver, we first examined the Human Protein Atlas,[18] which shows IFN-γ expression in healthy liver tissue but does not define the cellular source at the protein level. We therefore performed multiplex immunofluorescence on core needle biopsies from healthy donors (n = 7; Table S1), using diagnostic pathology-grade antibody panels to detect IFN-γ protein, CD3, CD56, CXCR6, and nuclear DAPI staining. An illustrative example is shown in Fig. 1A; additional illustrative overlays highlighting IFN-γ protein expression in CD56+ CXCR6+ CD3- NK cells are provided in Fig. S1C. Detailed staining overlays and machine learning-based cell selection workflow, including cell count validation, are provided in Figs. S1 and S2. This approach enabled spatial visualization of cytokine expression and cell phenotype within intact liver architecture, without the confounding effects of *ex vivo* stimulation. IFN-γ–producing lymphocytes were detected in both parenchymal and portal regions (Fig. 1B) and were uniformly CD3$^-$, CD56+, and CXCR6+, consistent with liver-resident NK cells.[11] For clarity, CD56$^-$ cells were not annotated in Fig. 1B, as they did not express IFN-γ above background, allowing a clearer view of the spatial distribution of IFN-γ–producing cells. Quantitative image analysis using AI-based tools, with mean fluorescence intensity (MFI) thresholds determined individually for each marker (see Supplementary Methods), confirmed that CXCR6+NK cells were the dominant *in situ* IFN-γ producers in healthy liver, with a median of 24.2% (IQR: 14.1–67.2%) expressing detectable protein. Comparison of absolute counts within each individual tissue section confirmed these results (Fig. 1C): CXCR6+NK cells were the most abundant IFN-γ–producing population (median 1,269 cells; IQR: 696–2,904), followed by CXCR6+NKT cells (median 94; IQR: 72–116.5), CXCR6+ CD3+ T cells (median 3; IQR: 1.5–4.5), and CD3+CD56$^-$CXCR6$^-$ T cells (median 2; IQR: 1–2.5). CD56+CXCR6$^-$ NK cells did not express IFN-γ above the background threshold. These data identify CXCR6+NK cells as the principal source of *in situ* IFN-γ in the healthy human liver.

Liver IFN-γ protein expression is not restored in NUC-treated HBV

To test the longstanding hypothesis that intrahepatic IFN-γ production is impaired in chronic HBV, we focused on patients receiving long-term NUC therapy. This clinical context minimizes inflammation-related immune activation and offers the closest approximation to healthy liver among HBV-infected individuals. It therefore provides a stable background to assess IFN-γ production without confounding interference from ongoing liver injury or viral replication. We analyzed liver biopsies from NUC-treated patients with HBV (n = 9), all with normal alanine aminotransferase (ALT) and no histological fibrosis (Table S1). Detailed staining images and absolute immune cell counts are provided in Fig. S2 and Fig. S1B, respectively. Multiplex immunofluorescence showed that CXCR6+NK cells in NUC-HBV samples rarely expressed IFN-γ protein (Fig. 2A; Fig. S2), with only 0.2% (IQR: 0.0–1.4%) staining positive (Fig. 2B). Despite a significant increase in CD3+ T-cell frequency in NUC-HBV livers (median 5.6%; IQR: 3.4–5.8) compared with healthy controls (median 1.6%; IQR: 1.5–1.9; p = 0.0007; Fig. 2C), these cells did not compensate for the loss of IFN-γ production. No other CD3+ or CD56+ subset contributed meaningfully to *in situ* IFN-γ expression (Fig. 2D).

The total number of CXCR6+NK cells was also reduced in NUC-HBV livers (median 675 cells; IQR: 704.5) compared with

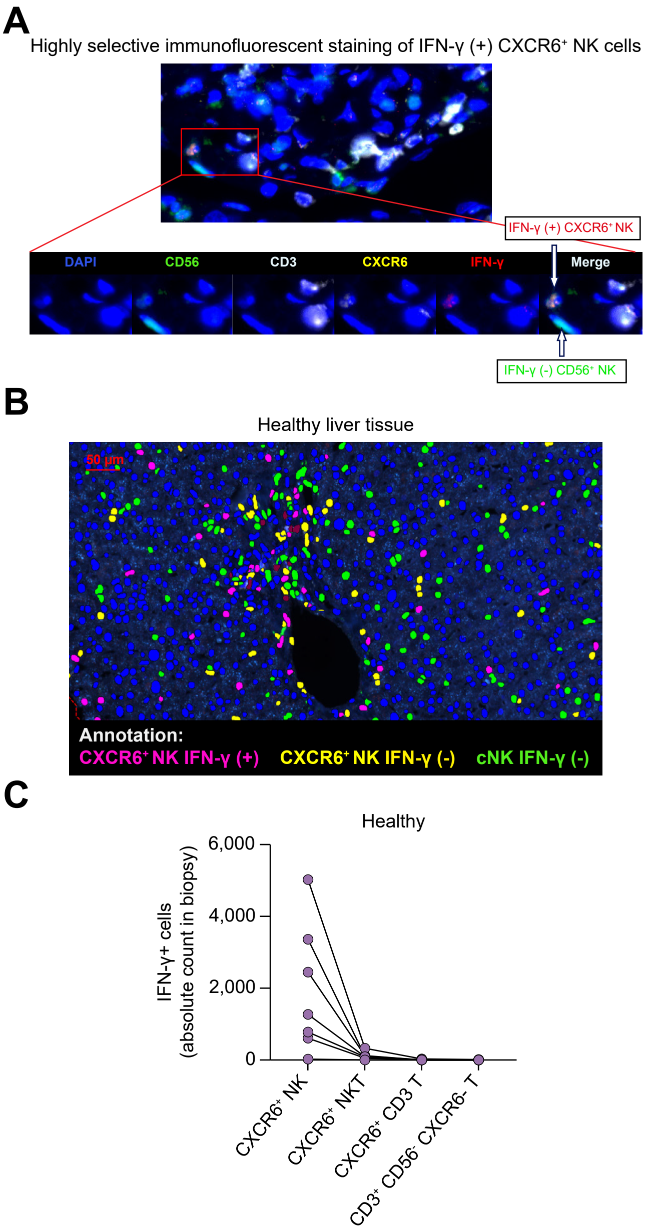

Fig. 1. CXCR6+NK cells are the dominant source of in situ IFN-γ in healthy human liver (A) Immunofluorescent staining to illustrate staining of IFN-γ+ CXCR6+NK cells in a healthy liver, using antibodies against CD56, CD3, CXCR6, IFN-γ, and nuclear staining with DAPI. Individual channels show DAPI (blue), CD56 (green), CD3 (white), CXCR6 (yellow), and IFN-γ (red). The merged image includes close-up views of a CD56 monostain, an IFN-γ+ CXCR6+NK cell, a CXCR6+ CD3+ cell, and a CD3 T cell. (B) Algorithmic phenotyping of immune cells in healthy liver tissue. Image analysis was performed using pathologist-supervised machine learning for unbiased identification and classification of immune cells. Mean fluorescence intensity (MFI) thresholds for DAPI, CD3, CD56, CXCR6, and IFN-γ were determined per slide and corrected for background. Every nucleated cell in the stained tissue was evaluated against these thresholds, and positive cells were phenotyped accordingly. For clarity, only CD56+ cells are color-coded in the algorithmic overlay. The control liver sample shows widespread parenchymal IFN-γ+ CXCR6+NK cells (pink), CXCR6+ IFN-γ- NK cells (yellow), CD56dim/bright NK cells (green), and CXCR6+ IFN-γ+ CD3+ NKT cells (bordeaux). (C) Comparison of absolute counts within each individual tissue section of IFN-γ+ cells per biopsy, in healthy livers, shown in a paired analysis with connecting lines representing matched samples. The highest number of *in situ* IFN-γ–producing cells was observed among CXCR6+NK cells, followed by CXCR6+NKT cells, CXCR6+ CD3+ T cells, and, lastly, CXCR6- CD3+ T cells. CXCR6+NK cells, CXCR6-positive natural killer cells; HBV, hepatitis B virus; IFN-γ, interferon-gamma; NUC, nucleos(t)ide analog; MFI, mean fluorescence intensity;.

healthy controls (median 1,918; IQR: 5,751), with their frequency among total liver cells declining from 7.8% to 2.2% (p = 0.012) (Fig. 2E).

These data show that the reduced intrahepatic IFN-γ signal in NUC-HBV is caused by both a lower proportion of IFN-γ+ CXCR6+NK cells and a numerical loss of this population. This supports the hypothesis that IFN-γ production is impaired in chronic HBV, even under conditions of effective viral suppression and absent inflammation.

Liver scRNAseq confirms depletion and loss of *IFNG* and cytokine gene expression in CXCR6+NK cells in NUC-HBV

To gain deeper insight into the transcriptional programs underlying the loss of IFN-γ in NUC-treated HBV liver, we performed scRNA-seq on liver FNA and blood samples from 18 patients who were HBeAg-negative with chronic HBV on long-term NUC therapy (median duration: 7 years), all with normal ALT and minimal fibrosis (F0–F1). Fig. S3 shows longitudinal HBV DNA and ALT measurements over up to 10 years, confirming durable viral suppression. For scRNAseq analyses, nine patients contributed paired liver and blood samples, with one liver sample excluded as a result of quality control. Publicly available scRNA-seq datasets from control livers (n = 5) and healthy PBMCs (n = 9) were integrated for comparison. Table S2 provides detailed information on sample origin (liver/PBMC), clinical data, and Gene Expression Omnibus accession numbers. After quality control and batch correction, 221,383 immune cells were retained for analysis (Fig. S4 and Supplementary Methods). Unsupervised clustering identified major innate and adaptive immune populations, including four distinct NK cell clusters. Fig. S4 provides detailed QC metrics, data integration performance, and cluster-level annotations. Among these, a single CXCR6+NK cell cluster was identified, exclusive to liver samples, and representing the dominant hepatic NK-cell population. This cluster was transcriptionally defined by high expression of cytokine-related genes including *IFNG*, *TNF*, *XCL1*, C–C motif chemokine ligand 3 (*CCL3*), and C–C motif chemokine ligand 4 (*CCL4*), and relatively lower levels of cytotoxic effectors such as *GZMB* and *PRF1*, distinguishing it from circulating CD56dim and CD56bright NK cells (Fig. 3A–D).

Consistent with our *in situ* data, CXCR6+NK cells were less frequent in NUC-HBV livers than in control liver. Although numerical comparisons should be interpreted with caution given potential differences in tissue sampling (FNAs *vs.* surgical resections), the observed reduction was evident both as a fraction of total immune cells (median 5% *vs.* 22%, p = 0.002) and within the NK compartment (61% *vs.* 73%, p = 0.026) (Fig. 3E).

Differential gene expression analysis, correcting for potential numerical and batch differences between datasets, showed that this liver-restricted CXCR6+NK cell cluster exhibited marked downregulation of *IFNG*, directly confirming the loss of IFN-γ observed at the protein level (Fig. 3F). Other effector genes linked to dendritic cell recruitment—including *XCL1*, *CCL3*, *CCL4*, and *CCL4L2*—were also reduced,

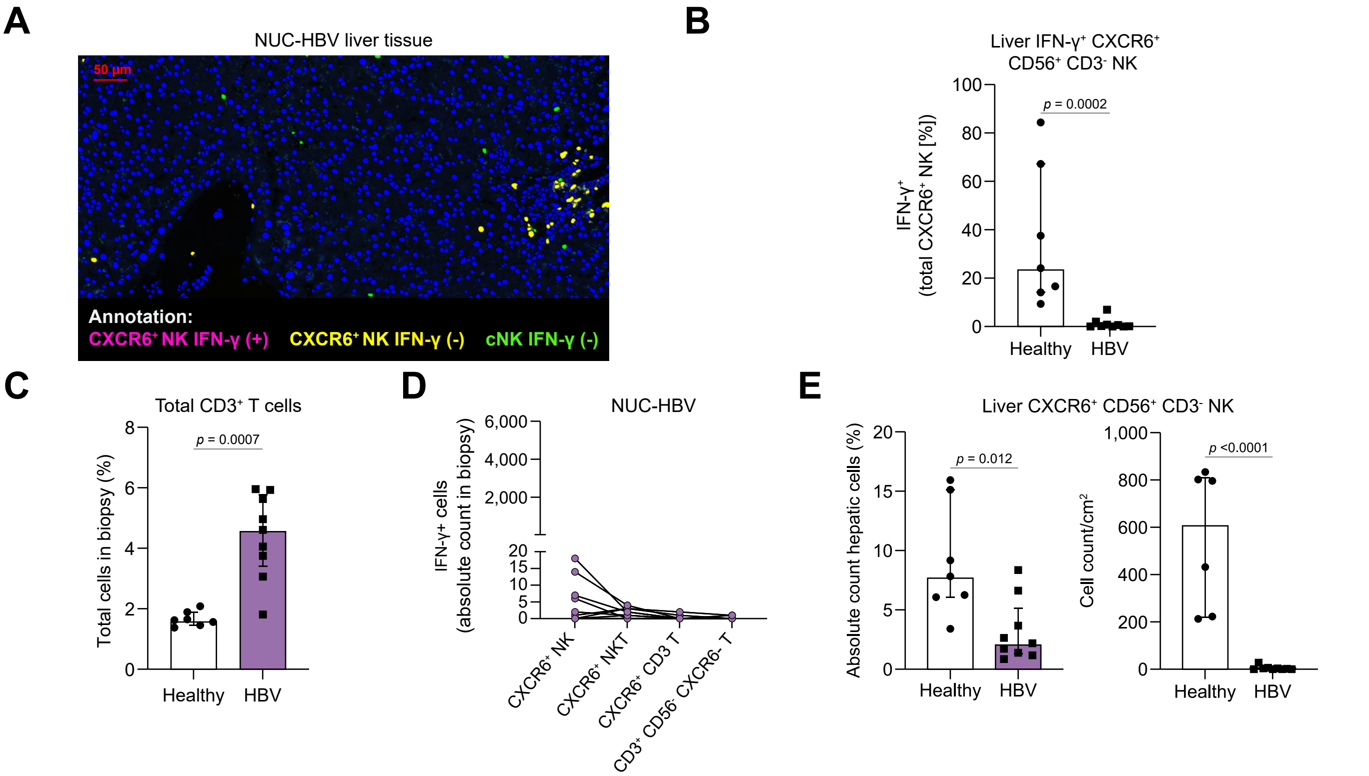

Fig. 2. Intrahepatic IFN-γ production by CXCR6+NK cells is lost in NUC-HBV (A) Algorithmic phenotyping of immune cells in NUC-HBV liver tissue, staining of IFN-γ+ CXCR6+NK cells in a healthy liver sample, using antibodies against CD56, CD3, CXCR6, IFN-γ, and nuclear staining with DAPI. (B) Frequency (%) of IFN-γ+ CXCR6+ CD56+ CD3- NK cells as a proportion of total CXCR6+NK cells in healthy and NUC-HBV liver biopsies. (C) Total CD3+ T-cell counts per biopsy in healthy controls and NUC-treated patients with HBV. (D) Absolute number of IFN-γ+ cells per biopsy, in NUC-HBV livers, shown in a paired analysis with connecting lines representing matched samples. (E) Frequency (%) of total detected cells and absolute density (cells per cm^2 of tissue) of CXCR6+ CD56+CD3- NK cells, compared between patients with NUC-HBV and healthy controls. Data are shown as median with interquartile range. Comparisons between healthy and NUC-HBV samples were performed using a two-tailed Mann–Whitney *U* test; $p < 0.05$ was considered significant. Abbreviations: CXCR6+NK cells, CXCR6-positive natural killer cells; HBV, hepatitis B virus; IFN-γ, interferon-gamma; NUCs, nucleos(t)ide analogs.

indicating a broader impairment in immunoregulatory output. In contrast, expression of key inflammatory cytokines such as *TNF* and chemokines including *CCL5* and *XCL2* remained intact (Fig. 3F). Together, these findings demonstrate that in NUC-HBV liver, CXCR6+NK cells undergo a selective and specific downregulation of cytokine programs, particularly *IFNG* and chemokine genes.

Liver CXCR6+NK cells retain cytotoxicity gene expression despite cytokine suppression

Given the profound cytokine suppression at the transcriptional level, we next examined whether cytotoxicity-associated genes were similarly affected. Expression of *GZMB*, *GZMK*, *FCGR3A*, *FASLG*, and *TNFSF10* remained unchanged in CXCR6+NK cells (Fig. 4A), indicating that the transcriptional changes are selective for *IFNG* and chemokines and do not extend to cytotoxicity-related genes. In contrast, several differentially expressed genes in NUC-HBV CXCR6+NK cells were transcription factors known to regulate NK cell development and effector function. *ID2* was markedly downregulated, whereas *BCL11B* and *ZBTB16* were upregulated in NUC-HBV (Fig. 4B). Given the significant regulatory impact of transcription factors, we also assessed subtle expression changes (fold change [FC] <2). This revealed downregulation of *GATA3* (FC −1.40) and upregulation of *TCF7* (FC 1.29) and *EOMES* (FC 1.61). Upregulation of TCF7, alongside downregulation of ID2, reflects a transcriptional program indicative of less mature NK cells with reduced capacity for IFN-γ production, whereas the upregulation of BCL11B and ZBTB16 is consistent with preserved cytotoxic potential.[19–23]

Liver immune signaling shifts toward TGF-β–associated pathways in NUC-treated HBV

Next, we asked whether the observed transcriptional cytokine defect in CXCR6+NK cells reflects broader regulatory shifts in the intrahepatic immune environment. We first examined expression of key immunoregulatory genes and found that *TGFB1* was broadly expressed across multiple immune clusters, including NK cells, whereas *IL10* expression was negligible (Fig. 4C). To systematically explore intercellular communication in the scRNAseq data (Table S2), we performed cell–cell interaction analysis using major intrahepatic immune populations. Compared with control liver, NUC-HBV samples showed a marked reduction in predicted pro-inflammatory cytokine interactions (Fig. 4D). Among the top 100 predicted ligand–receptor pairs, only one involved inflammatory signaling (*CCL3L1–CCR5*). In contrast, TGF-β–associated signaling emerged as a dominant feature in NUC-HBV liver. *TGFB1* was expressed by multiple immune subsets—including NK cells—and predicted to signal through *TGFBR1* and *TGFBR2* on both

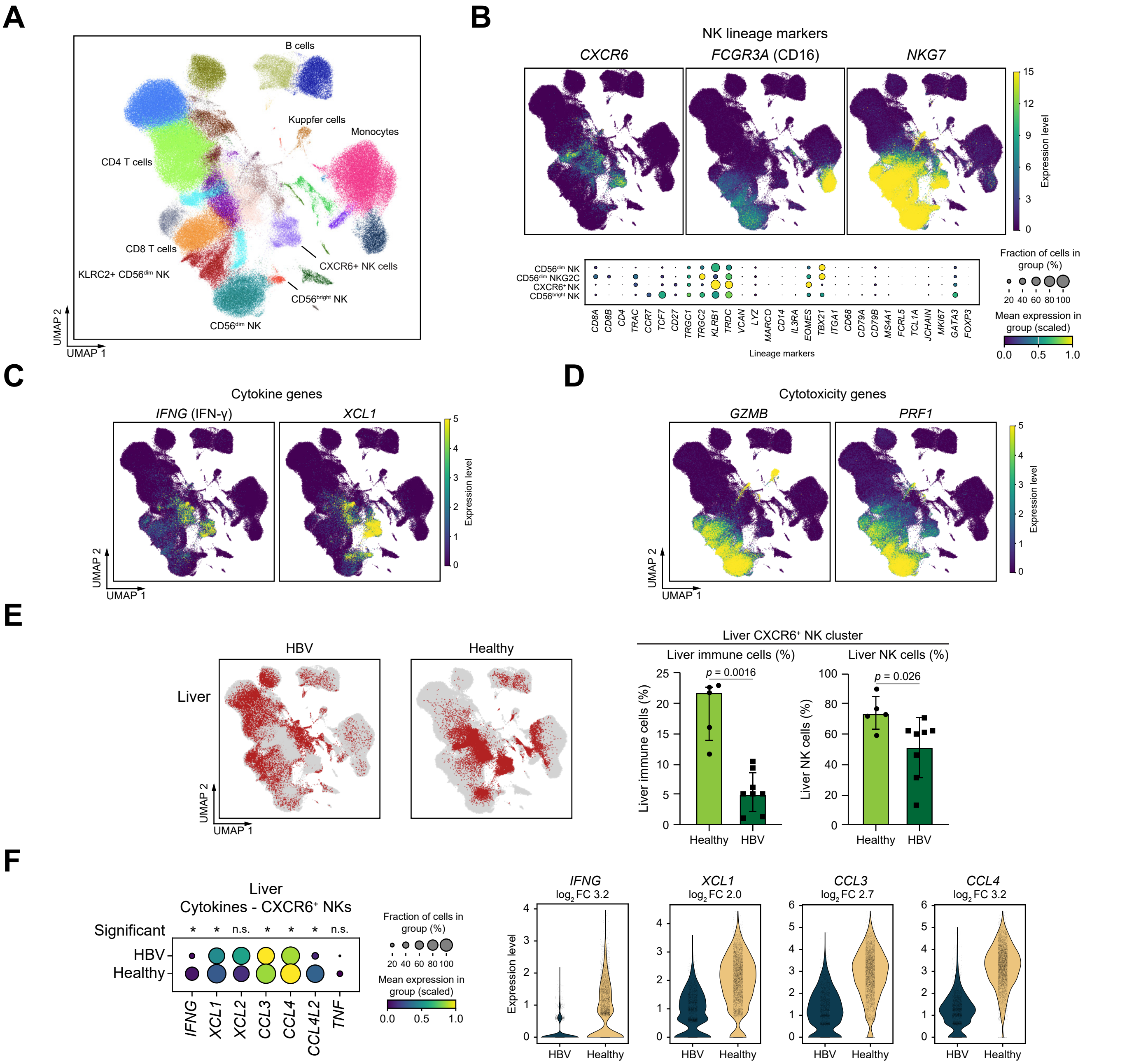

Fig. 3. scRNA-seq identifies a liver-restricted CXCR6+NK cell cluster with selective loss of cytokine gene expression in NUC-HBV (A) Uniform Manifold Approximation and Projection of immune subsets in liver and blood, with NK cell clusters highlighted. (B) Feature plots showing NK markers (*CXCR6, FCGR3A, NKG7*). (C, D) Cytokine (*IFNG, XCL1*) and cytotoxicity (*GZMB, PRF1*) genes in liver NK cells. (E) Left: UMAP plot depicting single-cell distributions from HBV and healthy liver tissues. Cells from HBV or healthy samples are highlighted in red, with all other cells shown in grey. The black circle outlines the cluster corresponding to CXCR6+NK cells; dots within this circle represent cells belonging specifically to this cluster. Right: Frequency of liver CXCR6+NK cells (cluster 13) in healthy and NUC-HBV, represented as a percentage of total liver immune cells and as total liver NK cells. (F) Left: Dotplot showing expression of differentially expressed cytokine genes in liver CXCR6+NK cells, scaled by mean expression for each group. Right: Violin plots highlighting the top significant cytokines, such as *IFNG* and *XCL1*. Frequencies are shown as median with interquartile range. Statistical comparisons between healthy and NUC-HBV liver CXCR6+NK cell frequency were performed using a two-tailed Mann-Whitney *U* test; p <0.05 was considered significant. Genes shown were identified as differentially expressed using a two-tailed Wilcoxon rank-sum test with Benjamini–Hochberg correction for multiple comparisons. Adjusted p <0.05 was considered significant. Abbreviations: CXCR6+NK cells, CXCR6-positive natural killer cells; HBV, hepatitis B virus; IFNG, interferon-gamma gene; XCL1, X-C motif chemokine ligand 1; GZMB, granzyme B; PRF1, perforin 1; NUC, nucleos(t)ide analog.

CD8 T cells and NK cells. Control liver samples exhibited more extensive pro-inflammatory crosstalk, largely driven by CXCR6+NK cell–derived chemokines such as *CCL3* and *CCL4*. In contrast, TGF-β–related interactions were not predicted in control liver. Together, these findings suggest that the immune environment in NUC-HBV liver is characterized by reduced cytokine network activity and a shift toward regulatory signaling, particularly involving TGF-β. This may contribute to the selective suppression of IFN-γ production in liver-resident NK cells.

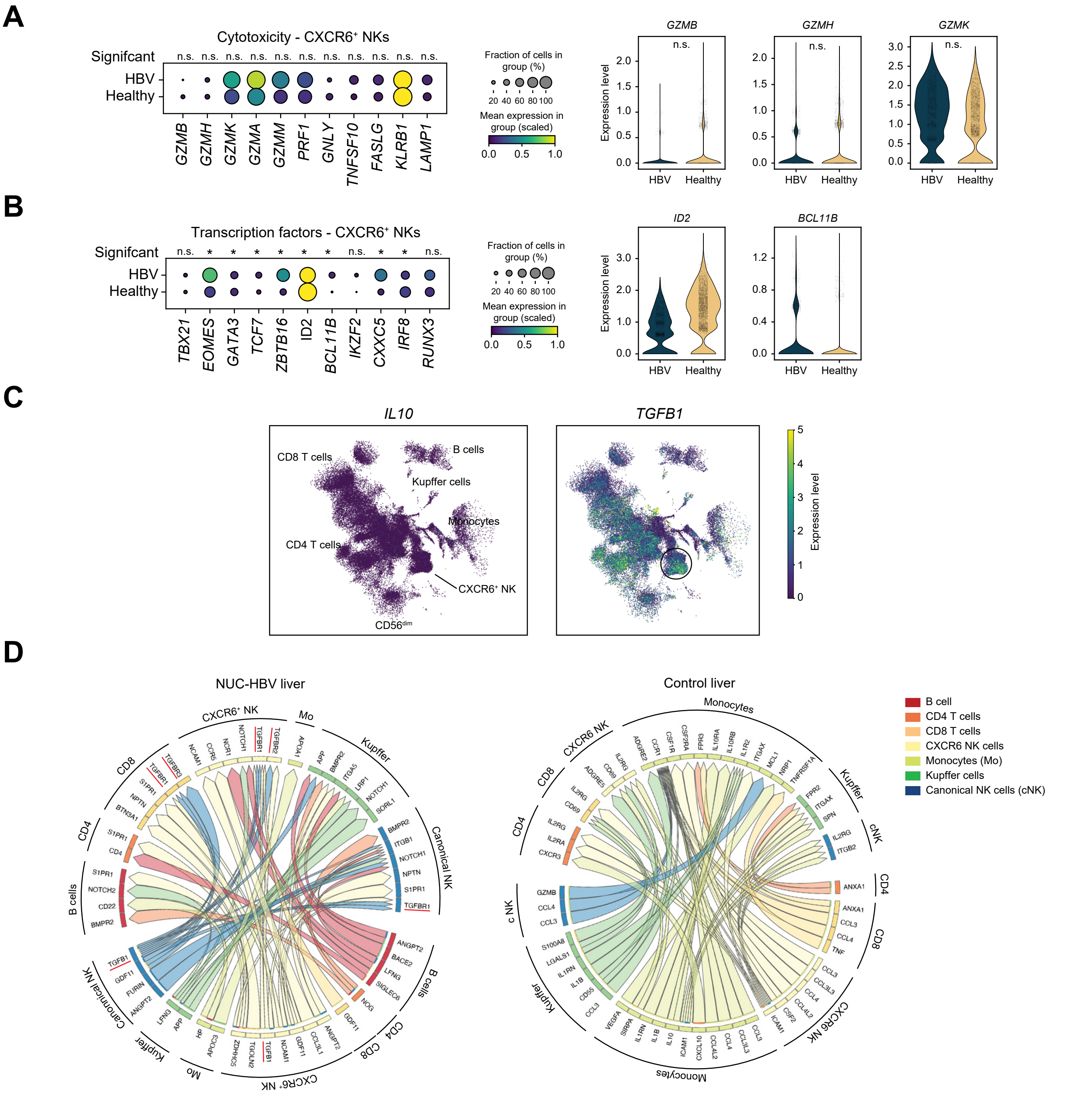

Fig. 4. Liver CXCR6+NK cells in NUC-HBV retain cytotoxic programs but show altered transcription factor and TGF-β–associated signaling (A) Left: Dotplot showing comparable expression of cytotoxicity-related genes (*e.g. GZMB*, *PRF1*) in CXCR6+NK cells across groups. Right: Violin plots displaying expression of key granzyme genes. (B) Left: Dotplot illustrating transcription factor expression (e.g., *ZBTB16, BCL11B*) in CXCR6+NK cells. Right: Violin plots showing significant transcription factors in HBV-affected NK cells. (C) Feature plots from liver *IL10* and *TGFB1* expression. (D) Top 100 receptor-ligand interactions within healthy and NUC-HBV liver with *TGFB1* underlined in red. Genes shown in panels (A and B) were identified as differentially expressed using a two-tailed Wilcoxon rank-sum test with Benjamini–Hochberg correction for multiple comparisons. Adjusted p <0.05 was considered significant. Color scale: yellow = increased expression; purple/blue = decreased expression.Abbreviations: CXCR6⁺NK cells, CXCR6-positive natural killer cells; HBV, hepatitis B virus; NUC, nucleos(t)ide analog; GZMB, granzyme B; PRF1, perforin 1; ZBTB16, zinc finger and BTB domain-containing protein 16; BCL11B, B-cell leukemia/lymphoma 11B; IL10, interleukin-10; TGFB1, transforming growth factor beta 1.

Liver-restricted loss of IFN-γ–producing CXCR6+NK cells is not observed in blood

To determine whether the reduction in CXCR6+NK cells and *IFNG* expression extends beyond the liver, we analyzed the scRNAseq dataset (Table S2) and an additional PBMC cohort (Table S3). In the scRNAseq dataset, no CXCR6+ liver-resident NK cell cluster was detected in blood (Fig. S5A). Circulating NK populations, including CD56dim and CD56bright clusters,

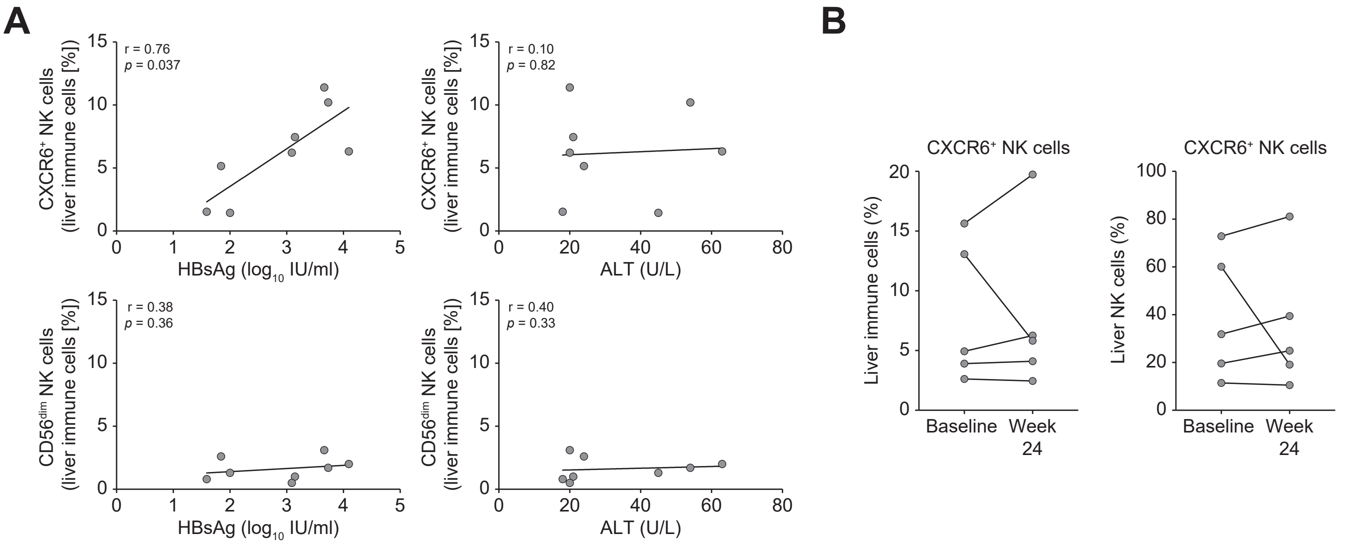

Fig. 5. Loss of CXCR6+NK cells correlates with HBsAg levels and is not restored during NUC therapy (A) Correlation plots showing the relationship between serum HBsAg levels (log_{10} IU/ml), ALT (U/L), and the frequency of CXCR6+NK cells (cluster 13) and CD56dim NK cells (cluster 4) as a percentage of total liver immune cells in FNAs from patients on long-term NUC therapy (n = 8). A full correlation matrix is provided in Table S4. Cohort details: Table S2. Spearman's rank correlation was used for correlation analysis, which does not assume normal distribution. Spearman R and two-tailed *p* values are reported; $p < 0.05$ was considered significant. (B) Frequency of CXCR6+NK cells in serial liver FNAs during 24 weeks of NUC therapy, displayed both as percentage of total liver immune cells and percentage of total liver NK cells (cohort details: Nkongolo *et al.*[24]). Statistical comparisons between baseline and week 24 were performed using a two-tailed Wilcoxon signed-rank test; $p < 0.05$ was considered significant. ALT, alanine aminotransferase; CXCR6+NK cells, CXCR6-positive natural killer cells; FNA, fine-needle aspiration; NUC, nucleos(t)ide analog.

showed no reduction in *IFNG* expression or consistent changes in cytokine gene expression between NUC-HBV (n = 18) and control blood (n = 9). Flow cytometry on the PBMC cohort (NUC-HBV, n = 44; healthy controls, n = 10; Table S3) confirmed that CXCR6+ cells were rare within the CD56brightNK compartment in blood and present at similar frequencies in both groups (Fig. S5B and C). These data indicate that the loss of IFN-γ–producing CXCR6+NK cells is confined to the liver and is not a systemic feature of NUC-treated chronic HBV.

Liver CXCR6+NK cell loss correlates with HBsAg levels and persists despite long-term NUC therapy

To explore potential clinical factors associated with reduced CXCR6+NK cell frequency, we analyzed the correlation between the NK cell clusters and clinical parameters within the scRNAseq NUC-HBV cohort (Table S2). No associations were observed with age, ALT, or treatment duration (Table S4). However, CXCR6+NK cell cluster frequency strongly correlated with serum HBsAg levels (Spearman R = 0.76, $p = 0.037$; Fig. 5A), suggesting a link between persistent HBsAg exposure and the intrahepatic loss of these cells.

Finally, we addressed whether the observed reduction in CXCR6+NK cells and their loss of *IFNG* expression reflects a general feature of chronic HBV infection or a specific consequence of NUC therapy. We reanalyzed a published longitudinal scRNAseq dataset of patients with HBV sampled before and after NUC initiation.[24] At baseline, these patients exhibited active liver disease with high HBV DNA and elevated ALT, both of which decreased upon treatment, while HBsAg levels remained stable. As shown in Fig. 5B, the single CXCR6+NK cell cluster present at baseline remained low in both frequency and IFNG expression over 24 weeks of NUC therapy. Fig. S6 provides detailed clustering, liver NK cell frequencies, and expression levels of key NK-cell cytokines and cytotoxic factors. Transcriptomic analysis confirmed stable expression of cytotoxicity genes and key transcription factors, with no recovery in cytokine genes such as *IFNG*, *XCL1*, *CCL3*, or *CCL4*. These findings support that the impaired cytokine profile of CXCR6+NK cells is not reversed by 24 weeks NUC therapy and may represent a feature of chronic HBV infection.

Discussion

Chronic HBV infection under NUC therapy is widely regarded as a state of sustained virological and biochemical control, yet our findings reveal a persistent deficit in intrahepatic immunity: the near-complete absence of *in situ* IFN-γ production by liver-resident CXCR6+NK cells obtained from patients with NUC-HBV. Using immunofluorescence on well-characterized archived liver biopsies, we identified CXCR6+NKs as the dominant source of IFN-γ in healthy liver. In contrast, NUC-treated HBV livers lacked not only IFN-γ–producing CXCR6+NK cells, but also any compensatory production by CD3+ T cells or other NK immune subsets. This establishes CXCR6+NK cells as a critical, non-redundant source of intrahepatic IFN-γ.

ScRNAseq confirmed and extended these findings. CXCR6+NK cells, exclusive to the liver, displayed the highest cytokine gene expression in healthy controls, yet were numerically and transcriptionally suppressed in NUC-HBV livers. Although cytotoxicity-related genes were preserved, the hallmark effector cytokines *IFNG* and chemokines (*e.g. XCL1* and *CCL4*) were profoundly downregulated. No similar changes were observed in circulating NK cells, underscoring that this cytokine defect is liver-restricted. In contrast to CD8+ T cells, which have been reported to show dynamic restoration of function during viral suppression,[24] CXCR6+NK cells remain in a low IFN-γ–

producing state. Analysis of untreated patients with HBV before and after NUC initiation revealed no recovery in CXCR6+NK cell frequency or cytokine expression, suggesting that this pattern is established early in infection and persists regardless of virological control or inflammation. These findings raise questions about the mechanisms sustaining this loss of cytokine production. A notable correlation between CXCR6+NK cell frequency and HBsAg levels, but not ALT or treatment duration, hints at persistent antigenemia (*e.g.* HBsAg) as a potential driver of *in situ* loss of cytokine production. Cell–cell interaction analyses further to a regulatory intrahepatic immune landscape in NUC-HBV, with loss of pro-inflammatory cytokine networks and TGF-β signaling—a known suppressor of NK cell function.[25]

Together, these data suggest that the loss of specific CXCR6+NK cell cytokines contributes to a broader attenuated immune state in HBV infection, and that the loss of cytokine producing CXCR6+NK cells may represent a bottleneck to immune restoration. Whether this reflects an irreversible epigenetic imprint, antigen-driven selection, or autocrine TGF-β feedback remains unclear. It is important to note that exhaustion-related gene programs (*e.g. PDCD1*, *HAVCR2*, *TOX*, *TOX2*, *CD274,* and *LAG3*), which have been suggested to be modulated during chronic viral stimulation[26] and shown to influence *IFNG* expression, were expressed at levels comparable with those in control liver (Fig. S7). Notably, murine models show that NK cells can release TGF-β1 in response to apoptotic cells, and that autocrine TGF-β is required for NK cell maintenance.[25,27] Similar pathways may operate in HBV-infected liver, but require further investigation. Our data align with recent observations in HBV-specific CD8+ T cells, where TGF-β dampens antiviral responses without classical exhaustion.[28] IFN-γ secretion by NK cells relies on cytokine priming and dendritic cell (DC) interactions, particularly through IL-12, IL-15, and IL-18, whereas cytotoxic function is maintained.[29] The observed loss of DC-attracting cytokines from CXCR6+NK cells suggests that their decline may further limit the recruitment and activation of DCs, potentially leading to a self-reinforcing loop of reduced immune activation in the HBV-infected liver. Restoring NK cell–derived IFN-γ may thus be a key strategy to reactivate intrahepatic immunity. Therapeutic avenues could include TLR8 agonists, IFN-α–based therapies, or novel vaccination platforms aimed at restoring IFN-γ cytokine production.

Our study has limitations. We performed FNA on patients with NUC-HBV, but comparison with healthy liver counterparts is notoriously difficult for obvious ethical reasons. Therefore, we first performed multiplex immunofluorescence on archived liver biopsies from healthy donors to establish baseline *in situ* IFN-γ expression, and then confirmed these findings using scRNAseq datasets generated from FNAs and biopsies. Although the control liver scRNAseq dataset we used is widely accepted as representing healthy liver, it was derived from patients undergoing resection for colorectal liver metastases.[15] Moreover, integrating datasets is essential to address the major challenge of obtaining truly healthy liver scRNAseq data, yet differences in sample origin, processing protocols, and 10 × Genomics versions may influence the expression of sensitive transcripts such as cytokines. To address this, we performed rigorous batch correction and confirmed robust dataset integration (Fig. S2). Nevertheless, we cannot fully exclude that these experimental differences have influenced the transcriptional profiles. Importantly, the scRNAseq data showed a selective loss of *IFNG* with preserved expression of other effector genes such as *TNF* and *XCL2*. This concordance across independent modalities strongly supports the validity of our conclusions.

In conclusion, our data support the longstanding hypothesis that intrahepatic IFN-γ production is impaired in NUC-treated chronic HBV infection. This deficit is specifically localized to CXCR6+ liver-resident NK cells, which are reduced in number and show consistently low protein and gene expression of IFN-γ, despite long-term viral suppression. Unlike CD8+ T cells, which may recover function during NUC, CXCR6+NK cells exhibit a stable reduction in IFN-γ and chemokine gene associated activity, even in the absence of inflammation. This intrahepatic loss of IFN-γ is not observed in peripheral NK cells and coincides with reduced pro-inflammatory cytokine interactions and increased TGF-β–associated signaling. These findings suggest a tissue-specific immune adaptation, potentially shaped by chronic antigen exposure (*e.g.* HBsAg) and local regulatory signals, that limits *in situ* IFN-γ production in CXCR6+NK cells. Targeting this bottleneck by restoring CXCR6+NK cell function or compensating for their reduced IFN-γ output, may be essential to achieving immune control and advancing HBV cure strategies.

Affiliations

[1]*Department of Gastroenterology and Hepatology, Erasmus Medical Center, Rotterdam, the Netherlands;* [2]*Internal Medicine IV (Gastroenterology, Hepatology, Infectious Diseases), University Hospital Heidelberg, Germany;* [3]*Molecular Virology, Center for Infectious Diseases, University Hospital Heidelberg, Germany;* [4]*Department of Pathology, Section Clinical Bioinformatics, Erasmus Medical Center, Rotterdam, The Netherlands;* [5]*Toronto Centre for Liver Disease, University Health Network, Toronto, Ontario, Canada;* [6]*Department of Pathology, Section Ophthalmic Pathology, Erasmus MC Cancer Institute, University Medical Center, Rotterdam, the Netherlands;* [7]*Department of Immunology, Erasmus University Medical Center, Rotterdam, the Netherlands*

Abbreviations

ALT, alanine aminotransferase; cccDNA, covalently closed circular DNA; CCL3, C–C motif chemokine ligand 3; CCL4, C–C motif chemokine ligand 4; CXCR6+NK cells, CXCR6-positive natural killer cells; FASLG, Fas ligand gene; FNA, fine-needle aspiration; GZMB, granzyme B; HCC, hepatocellular carcinoma; IFN-γ, interferon-gamma; *IFNG*, interferon-gamma gene; ISGs, interferon-stimulated genes; NK, natural killer; NUCs, nucleos(t)ide analogs; PBMCs, peripheral blood mononuclear cells; PLZF, promyelocytic leukemia zinc finger; PRF1, perforin 1 gene; scRNAseq, single-cell RNA sequencing; TBX21, T-box transcription factor 21; TGF-β, transforming growth factor-beta; TNFSF10, tumor necrosis factor superfamily member 10 gene; XCL1, chemokine (C-X-C motif) ligand 1.

Financial support

The Foundation for Liver and Gastrointestinal Research (SLO) sponsored the study. The funding source did not influence the study design, data collection, analysis and interpretation of the data, writing of the report, or the decision to submit for publication.

Conflicts of interest

The authors declare no conflicts of interest.

Please refer to the accompanying ICMJE disclosure forms for further details.

Authors' contributions

Conceptualized the study, wrote and performed analysis: BJBB. Performed bioinformatic analysis: DAK. Supervised the project: AB. Provided data: GWvO, ZMAG, KAL, AJG, TvdB, SN. Supervised bioinformatic analysis: HJGvdW. Clinical supervisor: RJdK.

Data availability

Data are publicly available and has been deposited in Gene Expression Omnibus (https://www.ncbi.nlm.nih.gov/geo/) with accession number: GSE247322. Multiplex immunofluorescence data are available from the corresponding author for academic, non-commercial purposes aimed at supporting transparency, reproducibility, or further research. Data will be shared in accordance with ethical approvals and institutional guidelines.

Supplementary data

Supplementary data to this article can be found online at https://doi.org/10.1016/j.jhepr.2026.101865.

References

[1] Hsu YC, Huang DQ, Nguyen MH. Global burden of hepatitis B virus: current status, missed opportunities and a call for action. Nat Rev Gastroenterol Hepatol 2023;20:524–537.
[2] Dusheiko G, Agarwal K, Maini MK. New approaches to chronic hepatitis B. N Engl J Med 2023;388:55–69.
[3] Yuen M-F, Heo J, Jang J-W, et al. Safety, tolerability and antiviral activity of the antisense oligonucleotide bepirovirsen in patients with chronic hepatitis B: a phase 2 randomized controlled trial. Nat Med 2021;27:1725–1734.
[4] Boeijen LL, Spaan M, Boonstra A. The effects of nucleoside/nucleotide analogues on host immune cells: the baseline for future immune therapy for HBV? Antivir Ther 2020;25:181–191.
[5] Genshaft AS, Subudhi S, Keo A, et al. Single-cell RNA sequencing of liver fine-needle aspirates captures immune diversity in the blood and liver in chronic hepatitis B patients. Hepatology 2023;78:1525–1541.
[6] Laouar Y, Sutterwala FS, Gorelik L, Flavell RA. Transforming growth factor-β controls T helper type 1 cell development through regulation of natural killer cell interferon-γ. Nat Immunol 2005;6:600–607.
[7] Phillips S, Chokshi S, Riva A, et al. CD8(+) T cell control of hepatitis B virus replication: direct comparison between cytolytic and noncytolytic functions. J Immunol 2010;184:287–295.
[8] Allweiss L, Volz T, Giersch K, et al. Proliferation of primary human hepatocytes and prevention of hepatitis B virus reinfection efficiently deplete nuclear cccDNA in vivo. Gut 2018;67:542–552.
[9] Guidotti LG, Rochford R, Chung J, et al. Viral clearance without destruction of infected cells during acute HBV infection. Science 1999;284:825–829.
[10] Lucifora J, Xia Y, Reisinger F, et al. Specific and nonhepatotoxic degradation of nuclear hepatitis B virus cccDNA. Science 2014;343:1221–1228.
[11] Hudspeth K, Donadon M, Cimino M, et al. Human liver-resident CD56bright/CD16neg NK cells are retained within hepatic sinusoids via the engagement of CCR5 and CXCR6 pathways. J Autoimmun 2016;66:40–50.
[12] Schuch A, Hoh A, Thimme R. The role of natural killer cells and CD8+ T cells in hepatitis B virus infection. Front Immunol 2014;5258.
[13] Tjwa ET, Zoutendijk R, van Oord GW, et al. Similar frequencies, phenotype and activation status of intrahepatic NK cells in chronic HBV patients after long-term treatment with tenofovir disoproxil fumarate (TDF). Antivir Res 2016;132:70–75.
[14] Oliviero B, Varchetta S, Paudice E, et al. Natural killer cell functional dichotomy in chronic hepatitis B and chronic hepatitis C virus infections. Gastroenterology 2009;137:1151. 60.e1157.
[15] Ramachandran P, Dobie R, Wilson-Kanamori JR, et al. Resolving the fibrotic niche of human liver cirrhosis at single-cell level. Nature 2019;575:512–518.
[16] Sinha S, Rosin NL, Arora R, et al. Dexamethasone modulates immature neutrophils and interferon programming in severe COVID-19. Nat Med 2022;28:201–211.
[17] Steele NG, Carpenter ES, Kemp SB, et al. Multimodal mapping of the tumor and peripheral blood immune landscape in human pancreatic cancer. Nat Cancer 2020;1:1097–1112.
[18] Uhlén M, Fagerberg L, Hallström BM, et al. Tissue-based map of the human proteome. Science 2015;347:1260419.
[19] Melsen JE, Lugthart G, Lankester AC, et al. Human circulating and tissue-resident CD56(bright) natural killer cell populations. Front Immunol 2016;7:262.
[20] Holmes TD, Pandey RV, Helm EY, et al. The transcription factor Bcl11b promotes both canonical and adaptive NK cell differentiation. Sci Immunol 2021:6eabc9801.
[21] Li ZY, Morman RE, Hegermiller E, et al. The transcriptional repressor ID2 supports natural killer cell maturation by controlling TCF1 amplitude. J Exp Med 2021;218:e20202032.
[22] Adams NM, Lau CM, Fan X, et al. Transcription factor IRF8 orchestrates the adaptive natural killer cell response. Immunity 2018;48:1172. 82.e1176.
[23] Samson SI, Richard O, Tavian M, et al. GATA-3 promotes maturation, IFN-gamma production, and liver-specific homing of NK cells. Immunity 2003;19:701–711.
[24] Nkongolo S, Mahamed D, Kuipery A, et al. Longitudinal liver sampling in patients with chronic hepatitis B starting antiviral therapy reveals hepatotoxic CD8+ T cells. J Clin Invest 2023;133:e158903.
[25] Chong WP, Zhou J, Law HKW, et al. Natural killer cells become tolerogenic after interaction with apoptotic cells. Eur J Immunol 2010;40:1718–1727.
[26] Marotel M, Villard M, Drouillard A, et al. Peripheral natural killer cells in chronic hepatitis B patients display multiple molecular features of T cell exhaustion. eLife 2021;10:e60095.
[27] Sparano C, Solís-Sayago D, Zangger NS, et al. Autocrine TGF-β1 drives tissue-specific differentiation and function of resident NK cells. J Exp Med 2024;222:e20240930.
[28] Heim K, Sagar Sogukpinar Ö, et al. Attenuated effector T cells are linked to control of chronic HBV infection. Nat Immunol 2024;25:1650–1662.
[29] Marçais A, Viel S, Grau M, et al. Regulation of mouse NK cell development and function by cytokines. Front Immunol 2013;4:450.

Keywords: Chronic hepatitis B; CXCR6 NK cells; NUC therapy; Liver immune cells; scRNAseq; TGF-β; Cytokine production; Viral suppression; Hepatitis B treatment.
Received 8 September 2025; received in revised form 9 April 2026; accepted 10 April 2026; Available online 18 April 2026

Journal of Hepatology, Volume *8*

Supplemental information

Persistent loss of intrahepatic IFN-γ in HBV is linked to selective impairment of liver-resident CXCR6+NK cells despite long-term NUC therapy

Boris J.B. Beudeker, Diren Arda Karaoglu, Shirin Nkongolo, Gertine W. van Oord, Zwier M.A. Groothuismink, Karishma A. Lila, Adam J. Gehring, Thierry van den Bosch, Robert J. de Knegt, Harmen J.G. van de Werken, and Andre Boonstra

Persistent loss of intrahepatic IFN-γ in HBV is linked to selective impairment of liver-resident CXCR6+NK cells despite long-term NUC therapy

Boris J B Beudeker, Diren Arda Karaoglu, Shirin Nkongolo, Gertine W van Oord, Zwier MA Groothuismink, Karishma A Lila, Adam J. Gehring, Thierry van den Bosch, Robert J de Knegt, Harmen JG van de Werken, Andre Boonstra

Table of contents

Supplementary methods..2

Supplementary figures..10

Supplementary tables..19

Supplementary methods

Study population

Chronic HBV patients visiting the outpatient clinic of Erasmus MC (Rotterdam, the Netherlands) were enrolled in this study. Included were HBeAg-negative adults (≥18 years) who had achieved stable viral control with entecavir or tenofovir (HBV DNA <80 IU/mL) for >3 years. Exclusion criteria were liver fibrosis (elastography >7.0 kPa or Metavir >F1), history of hepatic decompensation or hepatocellular carcinoma, co-infection with HCV, HDV, HEV, or HIV, autoimmune liver disease, severe steatosis or steatohepatitis, hemochromatosis, Wilson's disease, documented alcohol abuse, malignancy, treatment with vitamin K antagonists, or recent pregnancy. Clinical data were obtained from electronic medical records. Fibrosis was assessed by Fibroscan® or liver histology. Serum HBsAg levels (IU/mL) were measured with the Lumipulse G HBsAg assay (Fujirebio Europe) on a LUMIPULSE G1200 analyzer (Fujirebio Inc.).

Liver control samples were obtained from Ramachandran et al. (GSE136103), consisting of non-lesional tissue resected during surgery for solitary colorectal metastases. These individuals had no liver disease, normal biochemistry, and had not received neoadjuvant chemotherapy. Histological review confirmed absence of fibrosis or pathological alterations, making these samples widely accepted references for control liver tissue.

All participants provided written informed consent. The study was approved by the Erasmus MC Medical Ethics Committee.

Human peripheral blood mononuclear cells and fine-needle aspirates of the liver

At the outpatient clinic, heparinized peripheral blood and ultrasound-guided liver fine-needle aspirates (FNAs) were collected. PBMCs were isolated by Ficoll-Paque™ Plus (GE Healthcare Bio-Sciences AB), cryopreserved in FCS with 10% DMSO, and stored in vapor-phase liquid nitrogen at –150°C.

Intrahepatic leukocytes were obtained by FNA, a minimally invasive approach that has only been applied in a few single-cell studies to date. To minimize artifacts such as blood carryover or ischemia-related transcriptional changes, we implemented a validated rapid pipeline (reference 5) with immediate transport on ice and processing to single-cell droplet encapsulation in <1 hour. Compared with approaches that enrich CD45+ cells or use larger 20-gauge needles, our protocol

employs a finer 25-gauge Spinocan® needle, which yields cleaner aspirates enriched for intrahepatic leukocytes and minimizes red blood cell contamination.

For scRNA-seq, four passes were obtained per patient and collected in 500 µL colorless RPMI. Sample quality was assessed by optical density at 415–595 nm, with OD ≥0.19 indicating contamination by intrahepatic blood; such samples were excluded. Passes passing QC were pooled. Residual RBCs were depleted by incubation with Red Blood Cell Removal Solution (Stemcell) for 5 minutes, followed by two washes. Cells were counted, adjusted to 700–1200 cells/µL in RPMI + 5% FCS, and subjected immediately to single-cell encapsulation.

This validated workflow (5) ensures rapid and reproducible sampling of intrahepatic immune cells, providing high-quality material distinct from datasets generated by CD45+ sorting or large-bore biopsy approaches.

FFPE biopsies

Archived FFPE core liver biopsies archived at Erasmus MC were selected from HBV patients who had achieved successful long-term viral suppression on NUC therapy. These included biopsies obtained as study endpoints for NUC-related trials or at the initiation of NUC cessation. For comparison, liver biopsies from eligible altruistic healthy liver donors, collected to assess their eligibility as donors, were included. All tissue was processed in the same pathology facility to ensure consistent handling.

Phenotyping by flowcytometry

PBMC were thawed and washed with RPMI 1640 supplemented with 10% fetal calf serum (Lonza,). For flowcytometry, 250 000 viable PBMC were stained with antibodies against PD1-PerCP-eFluor710 (eBioJ105; eBioscience); TIM-3-PerCP (344823; R&D); CD56-APC vio770 (REA196; Miltenyi); TRAIL-Alexa Fluor488 (75402; R&D); CXCR6-APC (K041E5; Biolegend); TIGIT-BV786 (741182; BD Bioscience); KLRG1-PE (SA231A2; Biolegend); CD3-PEeFluor610 (UCHT1; eBioscience); HLA-DR-PE-Cy5 (LN3; eBioscience), CD38-PeCy7 (HB7; eBioscience), and viability-BV570 (Miltenyi); for 20 minutes at 4°C in the dark with the desired mixture of antibodies. Data were analyzed using FlowJo version 10.1 (Tree Star Inc.).

Sample preparation and scRNAseq on the 10x Genomics platform

Samples were prepared according to the 10x Genomics Single Cell 3' and 5' Reagent Kit user guide. Briefly, the maximum volume was loaded on a 10x Genomics Chromium to target capturing a maximum of 10,000 cells. After droplet generation, samples were transferred into a pre-chilled tube strip and cDNA was generated. The next day, cDNA was recovered using Recovery Agent provided by 10x Genomics and subsequently purified using a Silane DynaBead mix (Thermo Fisher) as outlined by the user guide. Purified cDNA was amplified for 13 cycles before being purified again using SPRIselect beads (Beckman Coulter). cDNA concentrations of the samples were determined on a Bioanalyzer (Agilent Technologies). Libraries were prepared as outlined by the 10x Genomics' Single Cell 3' and 5' Reagent Kit user guide, and sequenced (28-8-0-91 cycles) on a Novaseq6000 platform (Illumina).

Analysis of scRNAseq data

scRNAseq data from PBMC and liver samples were obtained as described above or obtained from publicly available datasets. For these public datasets, raw FASTQ files were used when available; otherwise, preprocessed H5AD files were utilized. For Steele et al. (1), data were retrieved from GEO (GSE157789), and metadata were obtained from the supplementary files of the manuscript. Similarly, for Sinha et al. (2), data were downloaded from GEO (GSE155698), with metadata sourced from the supplementary materials. For Ramachandran et al. (3), GEO (GSE136103) provided the raw data, while supplementary files from the manuscript and personal inquiry were used for metadata.

Raw FASTQ files were processed into count matrices using 10x Genomics Cell Ranger (v6.1.2) (4) with the human reference genome GRCh38 and default settings. To ensure a high-quality dataset, comprehensive quality control (QC) measures were applied. Cells were flagged as outliers if they deviated by more than 5 median absolute deviations (MADs) from the median for log-transformed total counts, log-transformed number of genes detected, or the percentage of counts in the top 20 most highly expressed genes. Cells with mitochondrial gene expression levels exceeding 3 MADs from the median or greater than 20% were also removed. These QC steps were performed following single-cell best practices (5). Ambient RNA contamination was corrected using SoupX (6). Cells expressing fewer than 200 genes and genes detected in fewer than three cells were filtered out using scanpy's filter_cells and filter_genes functions, respectively (7). After QC filtering, samples were integrated into a single dataset. Doublets were identified and flagged

using scDblFinder (8), ensuring a dataset with single-cell resolution. Out of 19 blood samples and 9 FNAs, one liver sample did not pass quality control and was excluded due to low-quality cells as described by the criteria above. Single-cell clustering was performed on 19 chronic hepatitis B infection PBMC samples, 9 healthy PBMC controls, 8 chronic hepatitis B infection fine-needle liver aspirates, and 5 healthy liver resections were included, retaining 221,383 cells for downstream analyses. Table S2 shows individual characteristics of our scRNAseq cohort.

Normalization was performed using scran (9), and highly variable genes were selected for subsequent steps. Dimensionality reduction using UMAP and batch effect correction were conducted with scVI (10). Clustering was performed using the Louvain algorithm at a resolution of 1.0, which identified 29 distinct clusters (Supplementary Data Table 2, Fig. S4A & H). This resolution was chosen to balance granularity and interpretability, allowing for the detection of biologically relevant immune subpopulations, including smaller subsets such as FOXP3+ CD4+ T cells and distinct NK cell subsets, without over-fragmenting known immune cell lineages (Fig. S4C).

To ensure robust immune cell classification, we performed reclustering (Fig. S4F), which did not result in the identification of additional NK cell subpopulations beyond those initially detected. Furthermore, we observed that all liver immune cell populations were present in both healthy and NUC-treated HBV samples, confirming a shared immune landscape across conditions (Fig. S4 D & H).

Marker genes for each cluster were determined using scanpy's rank_genes_groups function with Benjamini-Hochberg correction applied for multiple comparisons (gene list for annotation and cell counts in Fig. S4C and supplementary data table 1). Marker genes for each cluster were determined using scanpy's rank_genes_groups function with Benjamini-Hochberg correction applied for multiple comparisons.

Natural killer (NK) cells were identified based on essential markers and absence of T cell genes such as *CD3D*, *CD3G*, *CD4*, and *TRA*C, they included: CD56dim (Cluster 4): expressing transcription factor *TBX21* and chemokine receptor *CX3CR1+,* alongside ADCC and cytolytic factors (*FCGR3A+, GZMB+, PRF1+, GNLY, GZMA*), inhibitory markers (*HAVCR2* [TIM-3], KIRs). KLRC2+ CD56dim (Cluster 6): sharing largely overlapping differentially expressed genes with Cluster 4, but also expressing KLRC2, *TIGIT, KLRG1, LAG3,* and T-cell receptor-related genes (*TRGC2, CD3E* without other *CD3 genes*). CXCR6+ (Cluster 13): defined by the chemokine

receptor *CXCR6+,* activation marker *CD69,* transcription factor *EOMES,* abundant cytokine gene expression (*IFNG, TNF, XCL1, XCL2, CCL3, CCL4, CCL4L2, CCL5*), cytotoxic factors (*GZMK, GZMA, TNFSF10* [TRAIL]), and inhibitory factors (*KLRC1, TOX2, TIGIT*). CD56bright (Cluster 22): characterized by *NCAM1+, EOMES+, GZMK+, IL2RB+, KLRC1+, CD44+, SELL+*(Fig. S4, supplementary data table 1). This workflow provided a high-quality, well-annotated dataset for downstream analysis of. Differential gene expression analysis was performed to identify transcriptional shifts in NK clusters (Supplementary Data Table 2), focusing on biologically relevant changes (FC >2, adj. p <0.05). In liver, CXCR6+NK cells (Cluster 13) showed 83 DEGs, including downregulation of *IFNG*, *XCL1*, *CCL3*, *CCL4*, and upregulation of transcription factors (*TCF7, BCL11B, ZBTB16*). CD56dim NK cells (Cluster 4) displayed 74 DEGs, with overlap in *ID2* and cytotoxic genes (*GZMH, GZMK*), but not cytokines. In blood NK clusters, only minor shifts were observed (<20 DEGs per subset), with no loss of *IFNG*. Thus, major transcriptional alterations were liver-restricted. Cell-cell interaction analysis was conducted using MultiNicheNetR v2.0 (11). This type of analysis (Multinichenet) considers receptor-ligand pairs that are more strongly expressed in the condition of interest (HBV vs healthy), are cell-type specific, are present in most samples of the condition of interest, and for which predicted target genes are enriched in the receiver cell type. For the top 100 receptor-ligand interactions, cluster grouping was performed for greater statistical certainty and power. Groups included canonical NK cells (CD56dim and CD56bright NK cells), CXCR6+NK cells, monocytes, B cells, CD8+T cells, CD4+T cells, and Kuppfer cells.

Multiplex immunofluorescence

Multiplex immunofluorescence staining was performed on 4 µm FFPE liver biopsies from NUC-HBV patients and healthy controls in the ISO-certified pathology laboratory of Erasmus MC, ensuring diagnostic-grade reproducibility and quality control. Sections were processed on the automated Ventana Benchmark Discovery ULTRA system (Ventana Medical Systems Inc.). Following deparaffinization, antigen retrieval was performed with CC1 solution (Ventana, #950-224) for 32 minutes. To enable multiplex staining without cross-reactivity, sequential primary antibody incubations were interleaved with denaturation steps using CC2 solution (Ventana, #950-123) at 100 °C for 20 minutes.

The following antibodies were included in the multiplex panel: CXCR6 (rabbit polyclonal, Invitrogen, 1:100 dilution, 60 min at 37 °C), IFN-γ (rabbit polyclonal, Abcam, 1:100 dilution, 60 min at 37 °C), CD3 (rabbit monoclonal, clone 2GV6, Ventana, ready-to-use, 60 min at 37 °C), and CD56 (rabbit monoclonal, clone MRQ-42, Ventana, ready-to-use, 60 min at 37 °C). Detection was achieved with Omnimap anti-rabbit HRP (Ventana, #760-4311) in combination with appropriate chromogens: DCC (#760-244, Ventana) for CXCR6, Red610 (#760-245, Ventana) for IFN-γ, Cy5 (#760-238, Ventana) for CD3, and FAM (#760-243, Ventana) for CD56. All slides were counterstained with DAPI (Vector Laboratories) and mounted in Vectashield medium to preserve fluorescence.

Whole-slide imaging was performed using a Zeiss Axioscan 7 at high resolution, capturing both portal tracts and parenchymal regions in their entirety to avoid sampling bias. For quantitative analysis, images were processed using Visiopharm VIS (v2024.07.1.16912x64). Nuclear segmentation was carried out with a U-net deep learning model trained on hepatocyte and lymphocyte nuclei. Segmented nuclei were expanded radially to reconstruct cytoplasmic compartments, ensuring accurate classification of both membrane-associated and intracellular markers. This approach allowed unbiased quantification of up to 88,000 cells per slide, greatly exceeding the depth achievable by manual counting and reducing inter-operator variability.

To validate specificity, antibody performance was confirmed using diagnostic pathology–grade reference tissues (tonsil, placenta, lung), which were included on each staining run as internal positive and negative controls. In addition, hepatocytes within each liver biopsy served as an internal negative control for lymphocyte markers (CD3, CD56, CXCR6, IFN-γ), providing baseline autofluorescence thresholds. Positivity cut-offs were defined accordingly, ensuring that signals reflected true biological expression rather than background fluorescence.

In this analysis we focused specifically on T cells and NK cells, as they represent the principal lymphocyte sources of IFN-γ in the human liver. Within this compartment, the combination of CD56 and CXCR6 expression is uniquely restricted to NK cells and is not observed in other lymphocyte subsets. This biological constraint allowed us to confidently attribute IFN-γ signal to bona fide liver-resident NK cells, while signals from CD3+ cells were interpreted as T cell–

derived. By structuring the analysis in this way, we avoided misclassification of rare or noncanonical cell types and ensured that cytokine production was correctly assigned to the two dominant IFN-γ–producing lymphocyte lineages. The integration of automated high-quality staining, whole-slide scanning, and AI-based segmentation then enabled reproducible and unbiased quantification of IFN-γ production in situ, allowing direct comparison of NK- and T cell contributions between NUC-HBV and healthy liver tissue.

Statistical analyses

Analyses were performed with GraphPad Prism v8 and Python v3.11.3. Comparisons between groups used unpaired two-tailed Student's t-tests or nonparametric equivalents as appropriate. For multiple testing, Benjamini–Hochberg correction was applied. $P < 0.05$ was considered significant.

Reference:

1) https://pubmed.ncbi.nlm.nih.gov/34296197/
2) https://pubmed.ncbi.nlm.nih.gov/34782790/
3) https://pubmed.ncbi.nlm.nih.gov/31597160/
4) https://www.nature.com/articles/ncomms14049
5) https://www.nature.com/articles/s41576-023-00586-w
6) https://academic.oup.com/gigascience/article/9/12/giaa151/6049831?login=false
7) https://genomebiology.biomedcentral.com/articles/10.1186/s13059-017-1382-0
8) https://f1000research.com/articles/10-979/v2
9) https://f1000research.com/articles/5-2122/v2
10) https://www.nature.com/articles/s41587-021-01206-w
11) https://www.biorxiv.org/content/10.1101/2023.06.13.544751v1

Table with antibodies for IF

Antibody	Dilution	Species	Company	Clone	Ab incubation time at 37˚C
**CXCR6**	1:100	rabbit	Invitrogen	Polyclonal	60 minutes
**IFN-y**	1:100	rabbit	Abcam	Polyclonal	60 minutes
**CD3**	ready to use	rabbit	Ventana	2GV6	60 minutes
**CD56**	ready to use	rabbit	Ventana	MRQ-42	60 minutes

Supplementary figures

Fig. S1

A

Immunofluorescent staing with annotation of CD56+ cells in liver in healthy liver (HC IF 6)

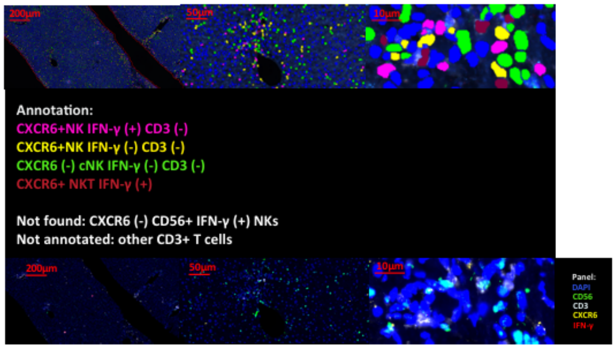

B

Absolute counts of CD56+ NK cells identified by immunofluorescent staining in liver tissue from healthy controls and NUC-treated HBV patients.

Group	cm2	CD56+ CD3- CXCR6- IFNy- cells	CD56+ CXCR6+ IFNy+/- CD3-	CD56+CXCR6+IFNy+ CD3-	CD3- CD56- CXCR6- IFNy-	Total cells	CXCR6NK/cm2
		CD56 cells	CXCR6+NK cells	IFNy+ CXCR6+NK cells	Negative cells	Total cells	
NUC-HBV 1	2.38	6297	458	1	32290	38957	192.22
NUC-HBV 2	1.71	555	765	6	27633	28929	448.14
NUC-HBV 4	0.81	311	1233	2	13217	14741	1518.47
NUC-HBV 5	1.76	269	234	0	27129	27626	133.22
NUC-HBV 6	0.96	181	999	0	13950	15102	1041.85
NUC-HBV 7	3.01	497	652	0	45726	46837	216.30
NUC-HBV 8	2.31	4302	689	14	34839	39773	297.06
NUC-HBV 9	0.65	319	258	18	11227	11795	395.45
NUC-HBV 10	2.07	2565	1114	7	26639	30276	537.14
HC IF 5	3.07	829	3641	2447	42063	46568	1184.23
HC IF 9	2.74	2981	2529	611	34949	40396	921.34
HC IF 6	6.26	9772	13374	5026	65538	88467	2134.79
HC IF 4	2.94	6215	7641	1269	34277	47926	2603.18
HC IF 3	3.67	12153	5514	781	42689	60131	1502.71
HC IF 7	4.03	1714	3982	3361	59795	65500	987.74

Supplementary Figure 1A & B. (A) Machine learning-based cell identification and color-coded annotation of IFN-γ+ immune cells in a healthy liver biopsy from the same donor shown in Figure 1. CD56+CXCR6+CD3− NK cells are consistently identified as the dominant IFN-γ–producing population. No IFN-γ+ CD56+ NK cells lacking CXCR6 expression (likely CD56dim and CD56bright subsets) were detected, (B) Table of CD56+ NK cell counts per sample, including absolute numbers in healthy and NUC-HBV liver samples

Abbreviations: HC IF; healthy control biospy for immunofluorescence, NUC-HBV; NUC-treated chronic HBV patient

Figure panel with individual markers in healthy liver tissue:
Multiplex immunofluorescence of DAPI, CD3, CD56, CXCR6, and IFN-γ, with merged image (lower panel) revealing CD56+ CXCR6+ IFN-γ+ CD3- NK cells in situ

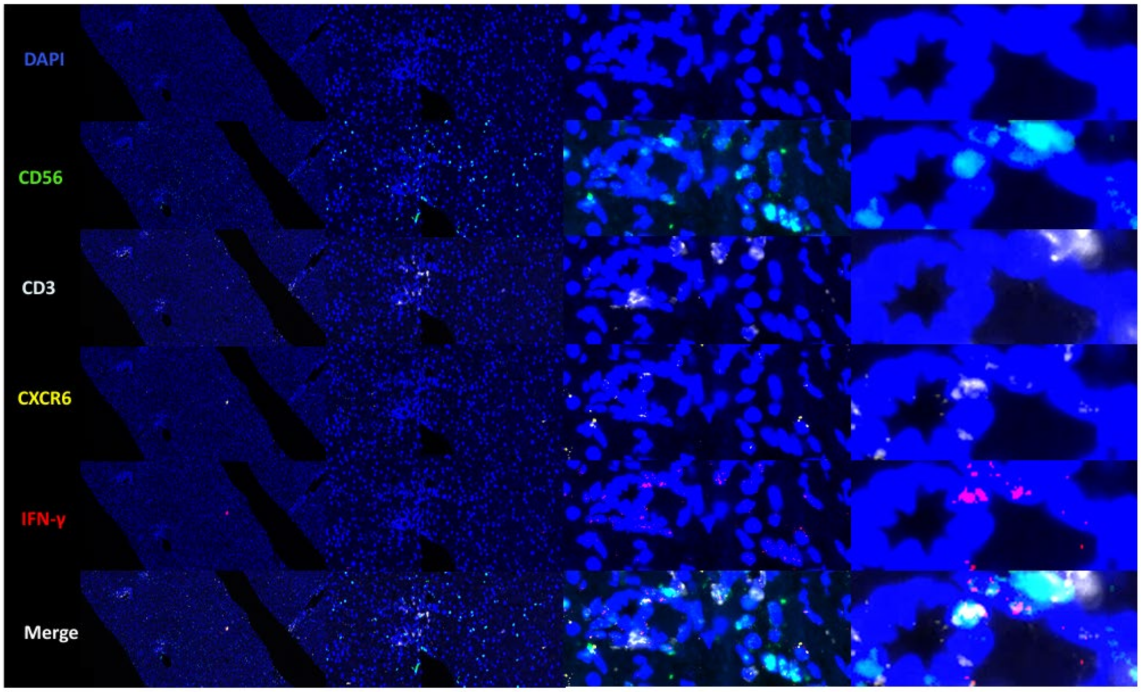

Supplementary figure 1C. Tissue section from the same healthy liver (Healthy 6 (HC IF 6)) donor shown in Figure 1. Each row represents a single stain: DAPI (nuclear), CD56 (NK cell marker), CD3 (T cell marker), CXCR6 (tissue residency), IFN-γ, and a final row showing the merged image. At higher magnification, IFN-γ signal is restricted to CD56+CXCR6+ cells, while neighboring CD56+CXCR6 (-) and CD56+ CXCR6 (+) NK cells remain negative, confirming high specificity of cytokine staining. This panel highlights the spatial colocalization of IFN-γ protein exclusively within CXCR6⊠ NK cells in healthy liver.

Fig. S2

Figure panel with individual markers in NUC HBV liver tissue:
Multiplex immunofluorescence of DAPI, CD3, CD56, CXCR6, and IFN-γ, with merged image (lower panel) revealing primarily CD3+ T cells in liver

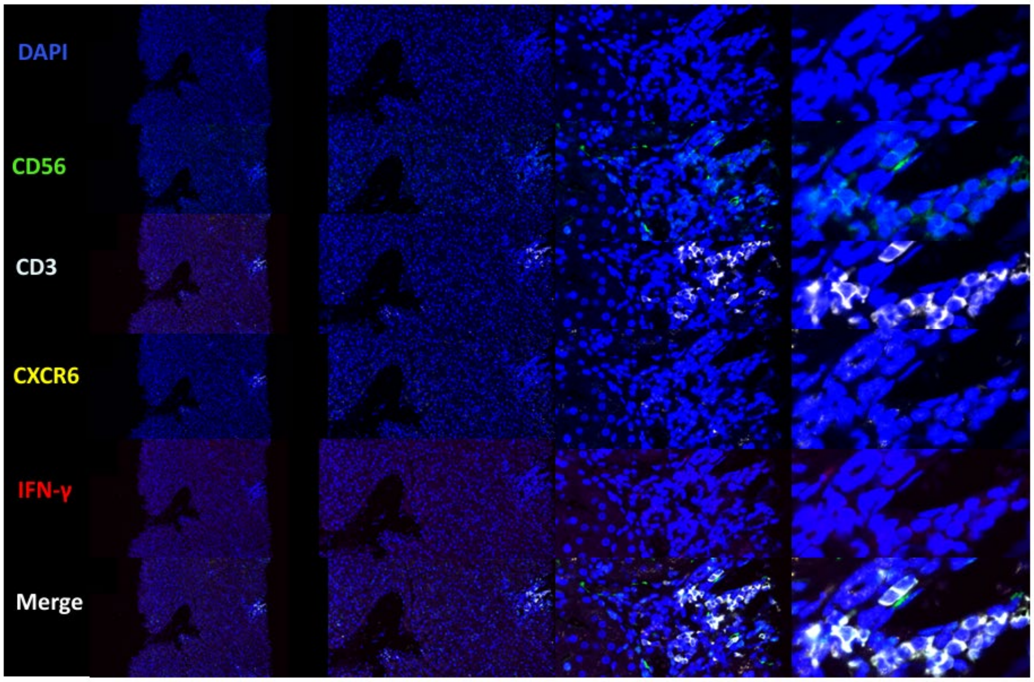

Supplementary figure 2. Tissue section from the same NUC-HBV liver sample shown in Figure 2. Each row displays a single-channel stain: DAPI (nuclear), CD56 (NK cell marker), CD3 (T cell marker), CXCR6 (tissue residency marker), IFN-γ, and a final row showing the merged image. At higher magnification, IFN-γ signal is nearly absent, and no CD56+CXCR6+ cells express IFN-γ. In contrast, a prominent accumulation of CD3+T cells is observed, particularly in periportal regions. This panel illustrates the striking loss of IFN-γ-producing CXCR6+NK cells in NUC-HBV liver, despite increased T cell presence.

Fig. S3

A

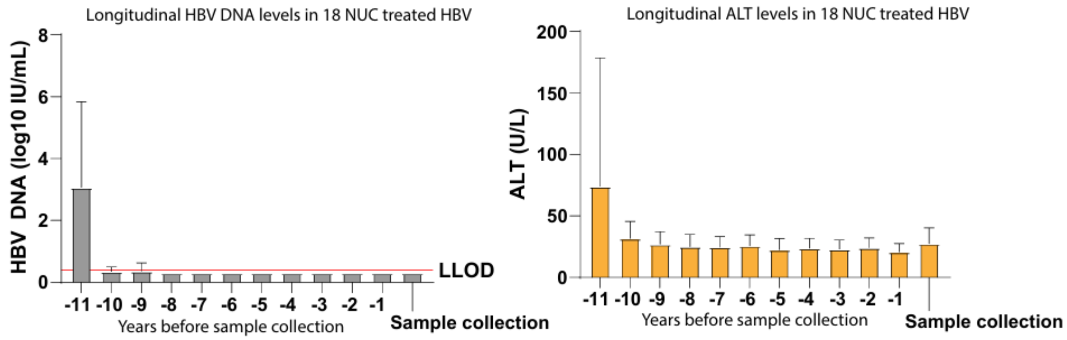

B

Sample ID	HBeAg (IU/ml)	Fibrosis score	Age	Sex	Type of antiviral	ALT (U/L)	HBsAg levels (IU/mL)	Genotype
**HBV1**	negative	F0-F1	36	male	Tenofovir	54	5385	ND
**HBV3**	negative	F0-F1	58	male	Tenofovir	26	74	C
**HBV4**	negative	F0-F1	43	male	Entecavir	63	12447	C
**HBV5**	negative	F0-F1	61	male	Tenofovir	20	4600	ND
**HBV6**	negative	F0-F1	52	male	Tenofovir	18	39	A
**HBV7**	negative	F0-F1	50	male	Tenofovir	45	100	ND
**HBV8**	negative	F0-F1	31	male	Tenofovir	21	1400	ND
**HBV9**	negative	F0-F1	67	male	Entecavir	24	70	B
**HBV10**	negative	F0-F1	59	male	Entecavir	20	1233	F
**HBV12**	negative	F0-F1	69	male	Tenofovir	9	4600	ND
**HBV13**	negative	F0-F1	62	male	Entecavir	36	68	B
**HBV14**	negative	F0-F1	47	male	Tenofovir	15	7195	A
**HBV15**	negative	F0-F1	58	male	Entecavir	34	2	C
**HBV16**	negative	F0-F1	48	male	Entecavir	28	79	B
**HBV17**	negative	F0-F1	59	male	Tenofovir	54	7573	A
**HBV18**	negative	F0-F1	48	male	Entecavir	21	920	ND
**HBV19**	negative	F0-F1	43	female	Tenofovir	24	1	C
**HBV20**	negative	F0-F1	44	male	Entecavir	23	99	C

Supplementary figure 3
(A)Trends in HBV DNA and ALT levels for 18 HBeAg-negative patients showing significant declines after entecavir or tenofovir treatment. (B)The lower panel shows clinical data for each patient.

Fig. S4

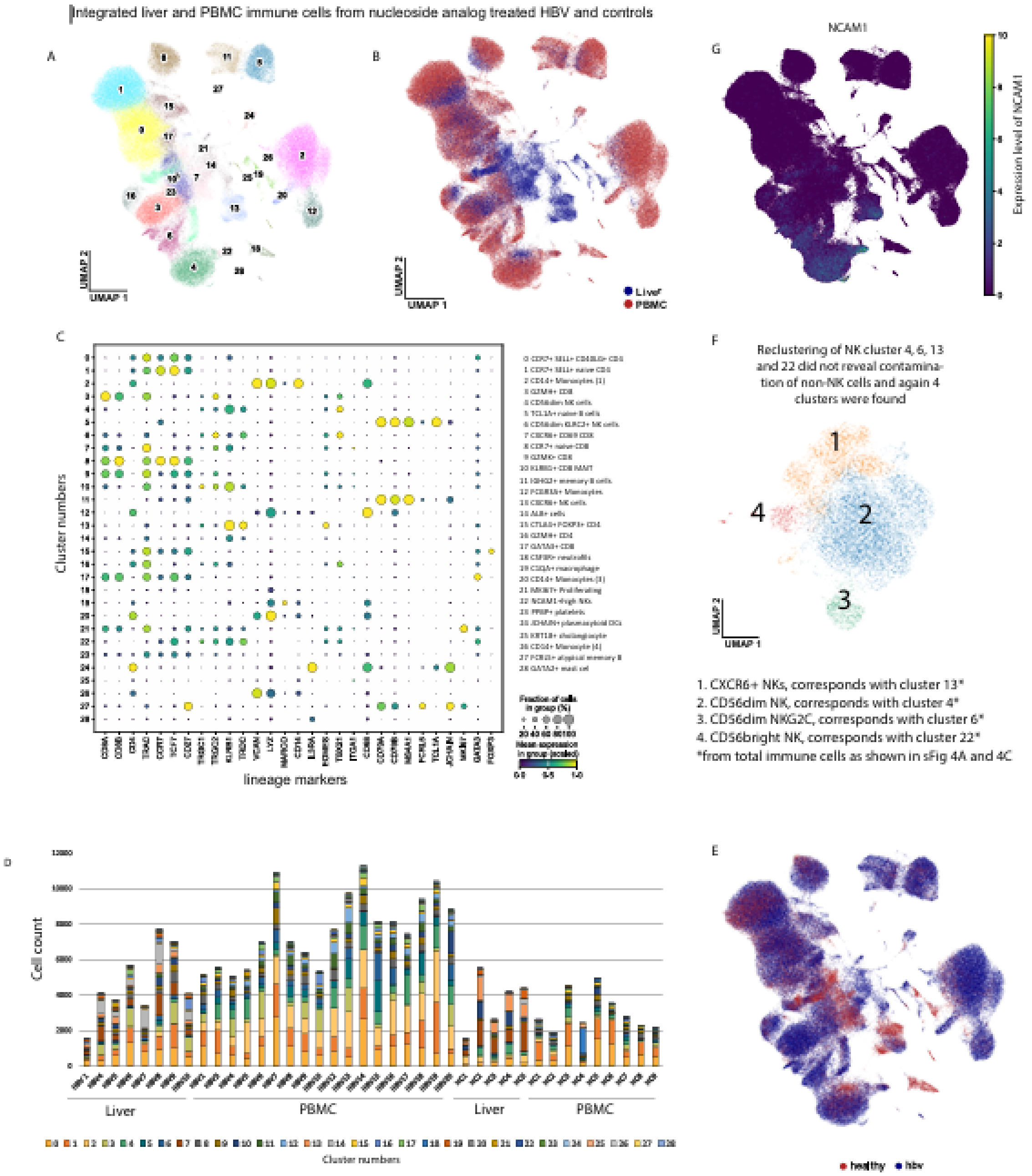

Supplementary Figure 4 A-G
Integration and Clustering of Stably Suppressed HBV Patients with Healthy Control Datasets
The effect of stably suppressed, NUC-treated HBV infection on liver NK cells remains unclear. To address this knowledge gap, we performed single-cell RNA sequencing (scRNAseq) to analyze the transcriptional profiles of liver immune cell populations in an integrated dataset. This dataset included ex vivo liver immune cells, ex vivo PBMCs, and liver and PBMC samples from healthy controls.
Our study compared paired PBMC and liver immune cells from HBV-infected individuals (N=9) and an additional 9 PBMC samples from NUC-treated HBV patients with those from healthy control liver (N=5) and PBMC samples, allowing us to elucidate HBV-specific liver changes. The dataset comprised samples from NUC-treated HBV subjects with stable suppression (median ALT: 26) (Supplementary Table 1), and high-quality healthy controls from Ramachandran et al. 2019 (GSE136103), Steele et al. 2020 (GSE155698), and Sinha et al. 2022 (GSE157789), all published by Nature.
Supplementary Figure 1 illustrates the successful integration of the PBMCs and liver immune cells from NUC-treated HBV and healthy control datasets and clustering of the immune populations from blood and liver (A and B). In the scRNA-seq data unsupervised clustering (Louvain set at 1.0) identified 28 distinct cell clusters representing both circulating and liver immune cells (C). These clusters included natural killer (NK) cells (NKG7, GNLY, SPON2, NCAM1 (G)), T cells (CD3D, TRAC), B cells (MS4A1, CD79A), plasma cells (IGHG1, IGHA1, JCHAIN), monocytes (CD14, FCN1, MNDA), and proliferating cells (MKI67, BIRC5).
Importantly, our analysis showed that the integrated PBMC data captured the full immunological diversity of both circulating and liver immune populations in all patients (D), demonstrating the robustness of our approach. We focused on clusters 4, 6, 13, and 22 for further analysis, specifically targeting NK cells. Clusters 4, 6, and 22 were found in both liver and blood (B) and were classified as conventional NK cells, while cluster 13 was only found in the liver and represented liver-resident CXCR6+ NK cells (A-C). These populations were selected for further NK cell analysis and underwent reclustering to obtain more detailed data on subtypes. However, reclustering did not reveal any unique NK cell phenotypes other than CD56dim, CD56bright, CD56dim NKG2C, or liver CXCR6 NK cells (F).

Batch correction figure

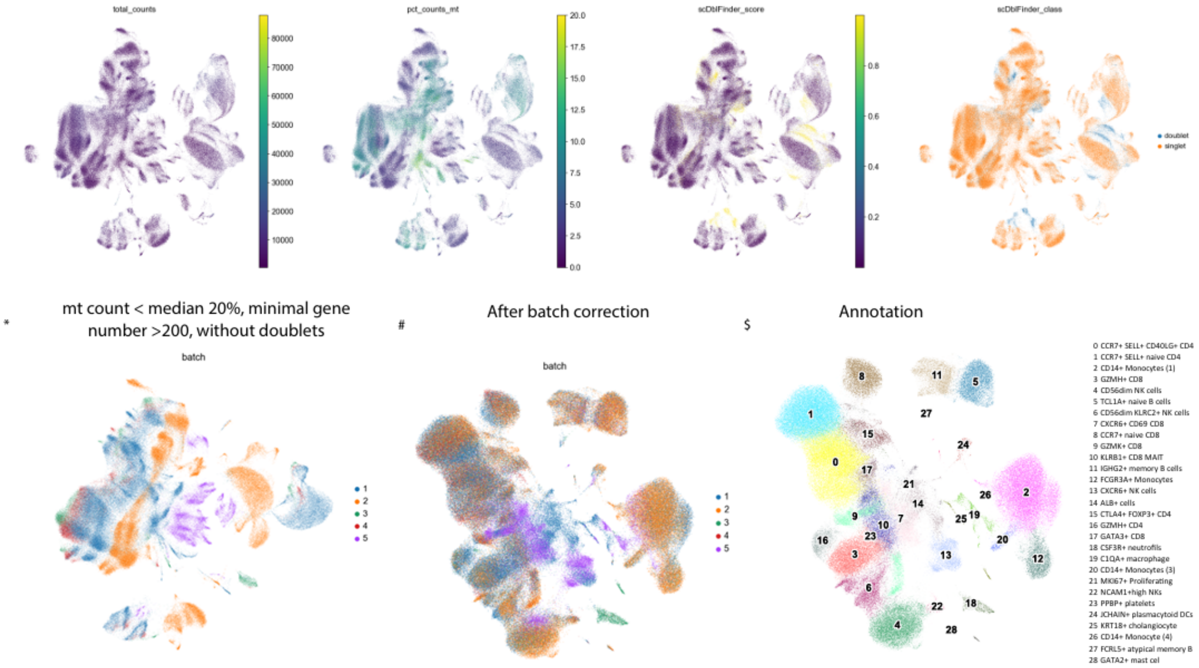

Batch number

1. HBV PBMC + Liver	(3' chemistry v3)	
2. HBV PBMC	(5' chemistry v2)	
3. Healthy PBMC	(3' chemistry v3)	(GSE155698)
4. Healthy PBMC	(3' chemistry v2)	(GSE157789)
5. Healthy liver	(3' chemistry v2)	(GSE136103)

Supplementary figure 4H. Batch correction and integration of scRNA-seq data from five distinct batches. Top panel: Overview of total counts (left), percentage of mitochondrial counts (middle), and scDbiFinder score and doublets (right) for all datasets. Bottom panel: (*) Before batch correction, showing distinct batch effects across the five datasets. (#) After successful integration of clusters, no chemistry-related batch effects are observed. Only cluster 8 (neutrophils) are specific to a PBMC dataset (3). Neutrophil capture are harder to detect with 10 × 3'v2 data because of their low RNA and high nuclease content. ($) Full annotation of clusters post-integration

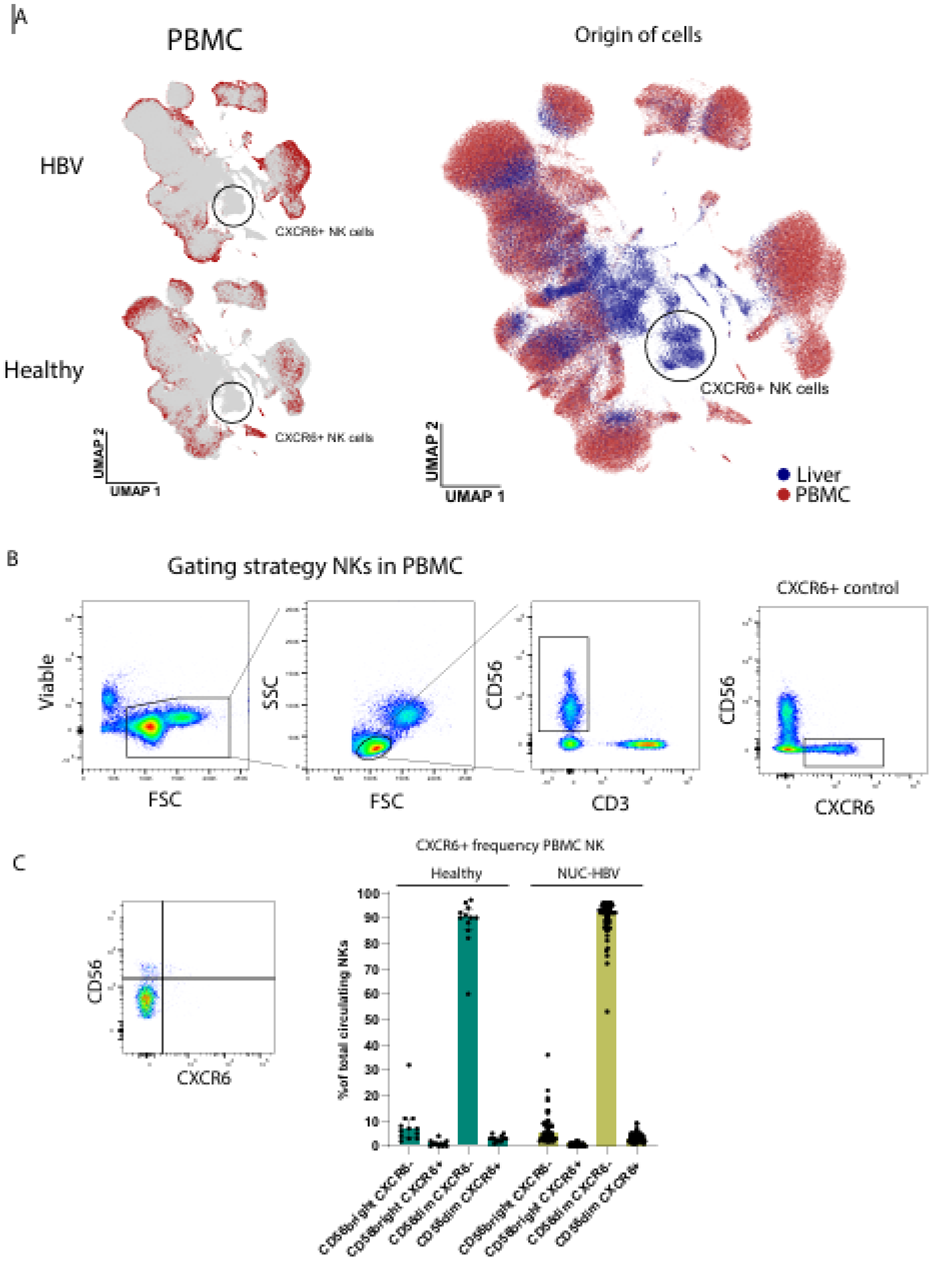

Supplementary Figure 5.
(A)Feature plot showing single-cell distribution in PBMCs from healthy and NUC-HBV patients, where red dots represent cells from the respective group, and grey dots indicate other cells. Right panel shows origin of cells, blue is liver and red is PBMC.
(B) Flow cytometry gating strategy for identifying circulating NK cells in 54 fresh frozen samples. The gating process includes the selection of viable lymphocytes, followed by the identification of CD56right and CD56dim CD3- NK cells. A sample with CXCR6high CD3+ cells is included as a positive control for the CXCR6 gate.
(C) CXCR6 expression is very low on circulating NK cells in both healthy individuals and HBV patients undergoing long-term NUC treatment. CXCR6+ CD56bright NK cells are rarely observed in blood, which is consistent with scRNAseq data indicating a distinct liver-specific cluster of CXCR6+ cells that is not present in PBMCs. Additionally, while low CXCR6 gene expression is observed in CD56dim NK cells in blood (flowcytometry and scRNAseq), these cells are distinct from the CXCR6+ EOMES+ IFNG+ liver NK cells (data not shown).

Fig. S5

Fig. S6

Post hoc analysis: 24-week NUC initiation in active HBV

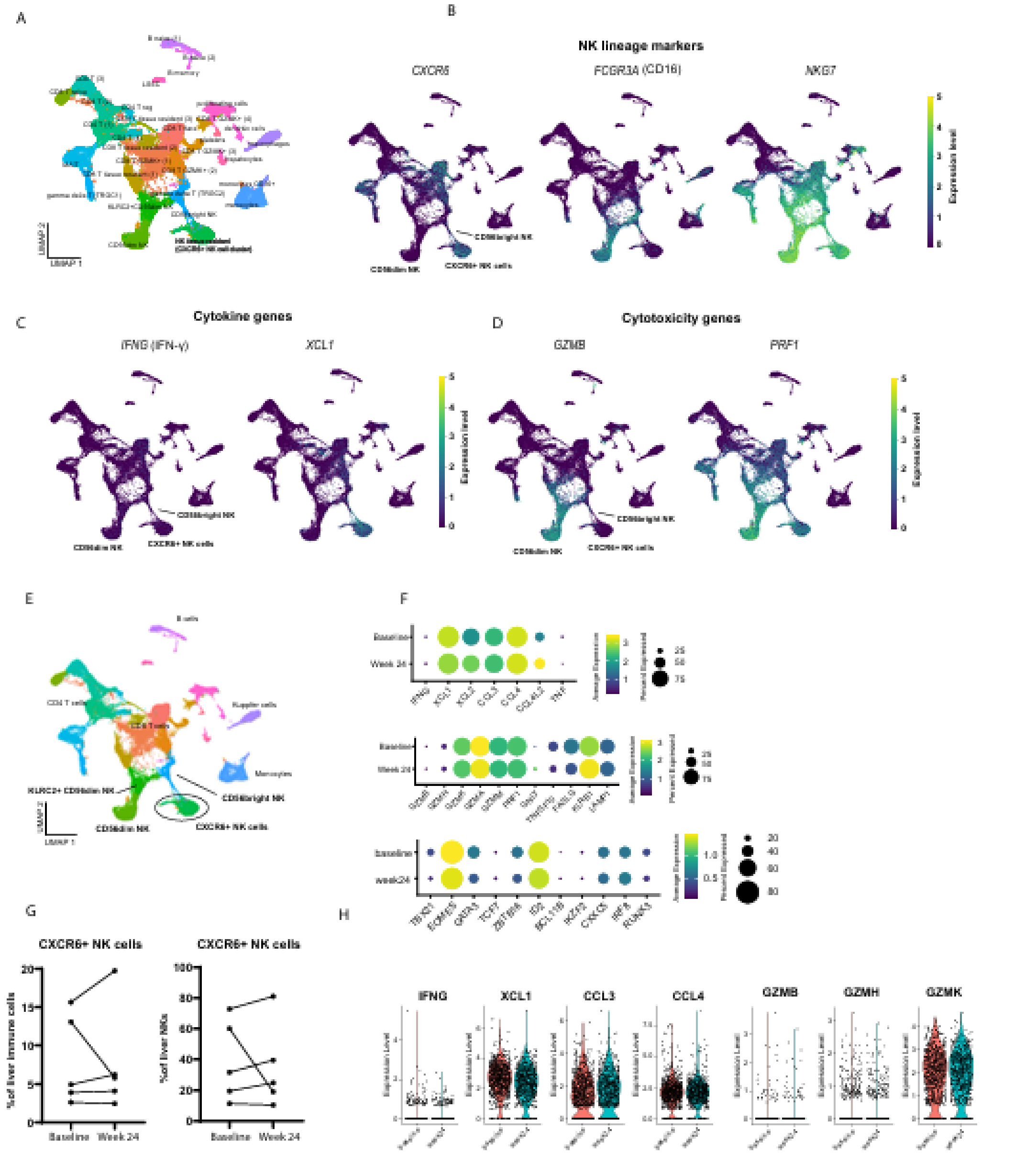

Supplementary figure 6
CXCR6+ NK cell annotation and cytokine profile in an independent scRNA-seq dataset
UMAP projections of liver immune cells from five chronic HBV patients sampled before and after NUC therapy (Nkongolo et al., J Clin Invest 2023;133(1):e158903) are shown, with annotation of major intrahepatic immune cell populations and NK cell subsets (A, E). CXCR6+ NK cells formed a distinct cluster alongside CD56dim NK cells, KLRC2+ CD56dim NK cells, and CD56bright NK cells—matching the subset distribution in our dataset.

Feature plots demonstrate comparable expression of NK lineage markers (B) and selected cytokine genes (C), with CXCR6+ NK cells showing low IFNG expression but preserved XCL1 expression (C, F, H), consistent with our findings. Cytotoxicity genes (PRF1, GZMB) were also low in CXCR6+ NK cells (D, F).

Panel F summarizes cytokine-, cytotoxicity-, and transcription factor–associated gene expression in dot plot format. Panel G shows stable CXCR6+ NK cluster frequencies over 24 weeks of NUC therapy. Panel H displays violin plots of selected gene expression, highlighting the absence of significant changes between baseline and week 24.

Fig. S7

A

Data origin: Liver (supplementary table 2 [scRNAseq of Liver FNA and control])

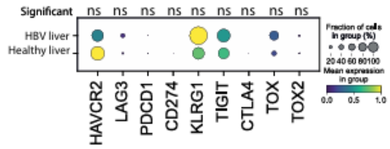

B

Data origin: Liver (Nkongolo et al., J Clin Invest 2023;133(1):e158903)

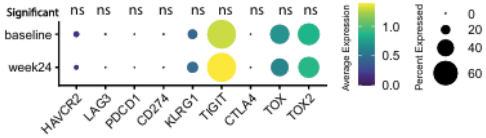

Supplementary Figure 7. Immune checkpoint markers in Liver and circulating NK cells
(A) Integrated PBMC scRNA-seq dataset (Supplementary Table 2) demonstrates comparable expression of immune checkpoint markers between NUC-HBV and controls in CXCR6+NK cells (clsuter 13). Color scale: Yellow indicates higher gene expression; blue indicates lower gene expression.
(B) Longitudinal PBMC scRNA-seq analysis during 24 weeks of NUC therapy (Nkongolo et al., J Clin Invest 2023) shows stable expression of immune checkpoint genes with no recovery or further decline over time.

Supplementary tables

Table S1

Group	ALT (U/L)	Ethnicity	Gender	Age	Metavir	Therapy
NUC-HBV 1	22	Caucasian	Male	39	F0-F1	Lamivudine
NUC-HBV 2	34	Asian	Male	40	F0-F1	Lamivudine
NUC-HBV 3	45	Asian	Male	50	F0-F1	Tenofovir
NUC-HBV 4	46	Caucasian	Male	38	F0-F1	Lamivudine
NUC-HBV 5	33	Caucasian	Male	46	F0-F1	Lamivudine
NUC-HBV 6	36	Asian	Male	28	F0-F1	Lamivudine
NUC-HBV 7	75	Caucasian	Male	35	F0-F1	Entecavir
NUC-HBV 8	29	Caucasian	Male	37	F0-F1	Adefovir
NUC-HBV 9	23	Asian	Female	45	F0-F1	Lamivudine
NUC-HBV 10	18	Caucasian	Male	48	F0-F1	Lamivudine
HC IF 1	NA	Asian	Male	38	F0	
HC IF 2	NA	Caucasian	Male	59	F0	
HC IF 3	21	Caucasian	Male	51	F0	
HC IF 4	61	Caucasian	Male	27	F0	
HC IF 5	24	Caucasian	Female	52	F0	
HC IF 6	28	Caucasian	Female	55	F0	
HC IF 7	NA	Caucasian	Female	63	F0	
HC IF 8	NA	Asian	Female	28	F0	
HC IF 9	24	Caucasian	Female	57	F0	

Supplementary table 1. Cohort of altruistic healthy liver donors (screening biopsies) and NUC-HBV patient biospies, collected as part of end of treatment biopsy or as screening for NUC-cessation. NA; not available, IF; immunofluorescence, HC; healthy control

Table S2

Sample ID	Type of patient	Database	Type of samples	Location of study	Sex	Type of antiviral	Age	ALT (U/L)	HBsAg levels (IU/mL)	Genotype
HBV1	NUC HBV	tbd	blood/liver	Rotterdam, Netherlands	male	TDF	36	54	5385	ND
HBV3	NUC HBV	tbd	blood	Rotterdam, Netherlands	male	TDF	58	26	74	C
HBV4	NUC HBV	tbd	blood/liver	Rotterdam, Netherlands	male	ETV	43	63	12447	C
HBV5	NUC HBV	tbd	blood/liver	Rotterdam, Netherlands	male	TDF	61	20	4600	ND
HBV6	NUC HBV	tbd	blood/liver	Rotterdam, Netherlands	male	TDF	52	18	39	A
HBV7	NUC HBV	tbd	blood/liver	Rotterdam, Netherlands	male	TDF	50	45	100	ND
HBV8	NUC HBV	tbd	blood/liver	Rotterdam, Netherlands	male	TDF	31	21	1400	ND
HBV9	NUC HBV	tbd	blood/liver	Rotterdam, Netherlands	male	ETV	67	24	70	B
HBV10	NUC HBV	tbd	blood/liver	Rotterdam, Netherlands	male	ETV	59	20	1233	F
HBV12	NUC HBV	tbd	blood	Rotterdam, Netherlands	male	TDF	69	9	4600	ND
HBV13	NUC HBV	tbd	blood	Rotterdam, Netherlands	male	ETV	62	36	68	B
HBV14	NUC HBV	tbd	blood	Rotterdam, Netherlands	male	TDF	47	15	7195	A
HBV15	NUC HBV	tbd	blood	Rotterdam, Netherlands	male	ETV	58	34	2	C
HBV16	NUC HBV	tbd	blood	Rotterdam, Netherlands	male	ETV	48	28	79	B
HBV17	NUC HBV	tbd	blood	Rotterdam, Netherlands	male	TDF	59	54	7573	A
HBV18	NUC HBV	tbd	blood	Rotterdam, Netherlands	male	ETV	48	21	920	ND
HBV19	NUC HBV	tbd	blood	Rotterdam, Netherlands	female	TDF	43	24	1	C
HBV20	NUC HBV	tbd	blood	Rotterdam, Netherlands	male	ETV	44	23	99	C
HC1	Healthy	GSE157789	blood	Calgary, Alberta, Canada	female		37			
HC2	Healthy	GSE157789	blood	Calgary, Alberta, Canada	male		64			
HC3	Healthy	GSE157789	blood	Calgary, Alberta, Canada	male		44			
HC4	Healthy	GSE157789	blood	Calgary, Alberta, Canada	male		44			
HC5	Healthy	GSE157789	blood	Calgary, Alberta, Canada	female		46			
HC1	Healthy	GSE136103	liver	Edinburgh, UK	male		59			
HC2	Healthy	GSE136103	liver	Edinburgh, UK	male		52			
HC3	Healthy	GSE136103	liver	Edinburgh, UK	male		70			
HC4	Healthy	GSE136103	liver	Edinburgh, UK	female		56			
HC5	Healthy	GSE136103	liver	Edinburgh, UK	male		50			
HC6	Healthy	GSE155698	blood	Michigan, USA	female		70			
HC7	Healthy	GSE155698	blood	Michigan, USA	male		71			
HC8	Healthy	GSE155698	blood	Michigan, USA	male		63			
HC9	Healthy	GSE155698	blood	Michigan, USA	male		60			

Supplementary Table 2: Clinical data of lon-term nucleos(t)ide analog-treated HBeAg-negative HBV subjects with scRNA-seq data from PBMCs and fine needle aspirate liver biopsies, including healthy subjects and their respective GSE accession numbers. Abbreviations: ALT (U/L) – Alanine Aminotransferase; HBsAg – Hepatitis B Surface Antigen; HBeAg – Hepatitis B e Antigen; TDF – Tenofovir; ETV – Entecavir

Table S3

		NUC-treatment	Healthy control	P-value
**N=**		**44**	**10**	
**Age (years)**	Median (IQR)	**47 (41-50)**	**49 (43-62)**	**0.2**
**Ethnicity**				**0.216**
Caucasian		**8 (19%)**	**5 (50%)**	
Asian		**13 (30%)**	**2 (20%)**	
Black		**7 (16%)**	**1 (10%)**	
Other		**16 (36%)**	**2 (20%)**	
**Sex**	M/F	**31/13**	**8/2**	**0.17**
**NA regime (ETV/TDF)**		**25/19**	**-**	**-**
**NA treatment duration (weeks)**	Mean (SD)	**384 (227)**	**-**	**-**
**HBeAg-negative**		**44 (100%)**	**10 (100%)**	**-**
**HBsAg baseline (IU/mL)**	Median (IQR)	**1450 (307-6050)**	**-**	
**HBV DNA (IU/mL)**	Median (IQR)	**Undetectable**	**-**	**-**
**ALT baseline (U/L)**	Median (IQR)	**23 (16-29)**	**30 (18-33)**	**0.44**
**Fibrosis F0-F1**		**44 (100%)**	**-**	**-**

Supplementary Table 3: Characteristics of the flow cytometry study cohort from Erasmus MC, Rotterdam, the Netherlands. Abbreviations: ALT (U/L) – Alanine Aminotransferase; HBsAg – Hepatitis B Surface Antigen; HBeAg – Hepatitis B e Antigen; TDF – Tenofovir; ETV – Entecavir

Table S4

		1	2	3	4	5	6	7	8
1	r=	—							
	p-value	—							
2	r=	-0.48	—						
	p-value	0.24	—						
3	r=	-0.44	0.60	—					
	p-value	0.27	0.12	—					
4	r=	0.57	-0.31	-0.29	—				
	p-value	0.15	0.46	0.49	—				
5	r=	0.31	0.38	0.40	0.45	—			
	p-value	0.46	0.36	0.33	0.27	—			
6	r=	-0.29	-0.14	0.24	-0.02	0.10	—		
	p-value	0.50	0.75	0.57	0.98	0.84	—		
7	r=	-0.21	**0.76**	0.10	-0.19	0.43	-0.07	—	
	p-value	0.62	**0.04**	0.82	0.66	0.30	0.88	—	
8	r=	0.05	-0.45	-0.42	0.02	0.07	0.38	0.07	—
	p-value	0.93	0.27	0.30	0.98	0.88	0.36	0.88	—

1. **Age**
2. **HBsAg** (IU/mL)
3. **ALT** (U/L)
4. **Treatment duration** (years)
5. **CD56dim NK cells (cluster 4)**
6. **CD56dim NKG2C^{+} NK cells (cluster 6)**
7. **CXCR6+ NK cells (cluster 13)**
8. **CD56bright NK cells (cluster 22)**

Supplementary table 4.
Correlation matrix of clinical parameters (age (years), serum HBsAg (IU/mL), ALT (U/L), and NUC treatment duration in months) with liver NK cell subset frequencies (CXCR6+ NK cells [cluster 13], CD56dim NK cells [cluster 4], CD56dim KLRC2+ NK cells, and CD56bright NK cells) in long-term NUC-treated HBV patients (cohort details supplementary table 2).
